# From Traps to Taxa: Building a Robust DNA Barcode Reference Library for the Early Detection of Invasive Cerambycidae

**DOI:** 10.1002/ece3.73626

**Published:** 2026-05-12

**Authors:** Loïs Veillat, Christian Cocquempot, Jean‐Claude Streito, Éric Pierre, Guénaëlle Genson, Jean‐Yves Rasplus, Astrid Cruaud, Julien Touroult, Alain Roques, Béatrice Courtial, Geraldine Roux, Carlos Lopez‐Vaamonde

**Affiliations:** ^1^ INRAE, UR633, Zoologie Forestière Orléans France; ^2^ Unité de Recherche en écologie des Forêts Méditerranéennes, INRAE Avignon France; ^3^ CBGP, INRAE, CIRAD, IRD, Institut Agro, University Montpellier Montpellier France; ^4^ UAR PatriNat (OFB, MNHN, CNRS, IRD) Paris Cedex 05 France; ^5^ Laboratoire Physiologie, Ecologie et Environnement P2E Université d'Orléans Orléans Cedex 2 France; ^6^ Institut de Recherche sur la Biologie de l'Insecte, CNRS UMR 7261, Université de Tours, UFR Sciences et Techniques Tours France

**Keywords:** biodiversity assessment, biosurveillance, COI, early‐detection, exotic species, multi‐pheromone blend, pest monitoring, pheromone trapping

## Abstract

Accurate and rapid identification of insect species is essential for effective biosurveillance, especially with increasing global trade and the spread of invasive pests. DNA barcoding has become a powerful tool for specimen identification, but its reliability depends heavily on the completeness and accuracy of reference databases. Many existing databases remain fragmentary and contain poorly annotated or misidentified records. Here, we present a curated DNA barcode reference library of 4097 DNA barcodes representing 169 species of longhorned beetles (Coleoptera: Cerambycidae), a group frequently intercepted in European biosurveillance programmes using multi‐pheromone traps. By prioritizing taxonomic validation and data quality, this library provides a robust resource for accurate specimen identification. Beyond biosurveillance, it also supports ecological research, as cerambycids are valuable bioindicators of forest health. Our results highlight the critical role of expert curation in ensuring the accuracy of barcode libraries used for both pest monitoring and biodiversity assessment.

## Graphical abstract

This work presents a curated DNA barcode reference library covering 147 species of longhorned beetles commonly intercepted in European biosurveillance programmes. By focusing on taxonomic validation and data quality, this resource improves the accuracy of specimen identification despite the limitations of existing databases. It also supports ecological research and highlights the key role of expert curation in both pest monitoring and biodiversity assessment.

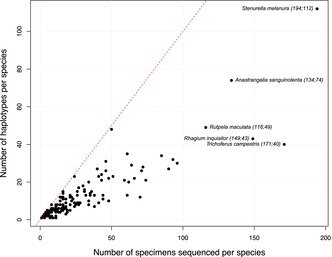

## 1 Introduction

Over the past few decades, intercontinental trade has expanded dramatically, resulting in a substantial rise in the introduction of non‐native insects beyond their natural ranges (Seebens et al. 2021). In Europe, this rise is mainly due to phytophagous insects, particularly those associated with woody plants, accounting for 76.5% of all new phytophagous species recorded between 2000 and 2014 (Roques et al. 2016). This trend is largely driven by the international trade of ornamental plants and the widespread use of wooden packaging materials, such as pallets and crates (Roques et al. 2020; Lovett et al. 2016).

Cerambycid beetles are among the exotic phytophagous insects most frequently intercepted in international quarantine (Haack 2006). Globally, the family Cerambycidae comprises an estimated 34,000 to 38,000 described species (Tavakilian and Chevillotte 2024), with an estimated number of European species ranging between 730 and 750 (including the Azores, Canary Islands, Cyprus, and up to the Urals) (Löbl and Smetana 2010; Sama 2023; Danilevsky 2025) and 250 species in mainland France, including Corsica (Touroult et al. 2019). The larvae of many cerambycid species develop under the bark of woody plants, feeding on the wood and forming galleries, making them difficult to detect. Their concealed and often prolonged development greatly facilitates their inadvertent spread via wooden packaging and the trade of plants with sufficiently large stems (Eyre and Haack 2017). As a result, an increasing number of cerambycid species have become invasive in forest and orchard pests (Venette and Hutchison 2021), causing tree mortality and damages of timber resources (Solomon 1995).

One promising approach for the early detection of exotic cerambycids is the use of traps baited with a combination of host volatiles (ethanol, alpha‐pinene) and cerambycid multi‐pheromone blends designed to ensure broad attractiveness, thereby enabling the collection of a wide range of cerambycid species (Fan et al. 2019; Roques et al. 2023). Such baited traps deployed all over the world attracted a broad spectrum of species across Cerambycidae subfamilies, capturing large numbers of individuals for some of them, thereby increasing the likelihood of detecting their arrival in a non‐native region (Roques et al. 2023). However, processing and identifying such large specimen numbers presents a major challenge. Traditional biosurveillance mostly relies on morphological identification, which is time‐consuming and dependent on specialized taxonomic expertise (Poland and Rassati 2019) but see Bonants et al. (2010). DNA barcoding has emerged as a powerful complementary tool, enabling faster and more standardized identification (Hebert et al. 2003), including of otherwise intractable life stages such as larvae and eggs (Hodgetts et al. 2016; Wu et al. 2017; Sire et al. 2025). A barcoding database of cerambycid species included in the European quarantine list has already been developed in the late 2000s (Bonants et al. 2010). However, during the recent decades most species of insects which established in non‐native regions were so‐called “emerging species”, that is, have never invaded regions other than the native one, and thus mostly absent from the quarantine lists on which the phytosanitary inspectors rely for control at ports of entry (Seebens et al. 2018).

The success of DNA barcoding depends critically on the availability of high‐quality reference libraries supported by expert‐based species identifications (Doorenweerd et al. 2024). These libraries typically include the standard COI barcode fragment (658 base pairs), taxonomic identifications, and associated metadata such as collection data and photographs. The main global repository, the Barcode of Life Data Systems (BOLD; www.boldsystems.org), facilitates the submission, analysis, and sharing of barcode data (Ratnasingham and Hebert 2007). Its “Barcode Index Number” (BIN) system clusters sequences into operational taxonomic units (Ratnasingham and Hebert 2013). Despite its utility, the system still faces important limitations, including incomplete coverage for many taxa and regions (Wu et al. 2017), misidentifications or poor annotations (Boykin et al. 2012; Bengtsson‐Palme et al. 2016), and biological complications such as incomplete lineage sorting, introgression, heteroplasmy, pseudogenes, and hybridization (Ashfaq and Hebert 2016; Porter and Hajibabaei 2018; Phillips et al. 2019).

Although interest in applying DNA barcoding for biosurveillance has grown (Floyd et al. 2010; Ashfaq and Hebert 2016; Wu et al. 2017; Madden et al. 2019; Lyal and Miller 2020; Javal et al. 2021; Doorenweerd et al. 2023; Doorenweerd and Barr 2025), no dedicated and comprehensive barcode reference library currently exists for European cerambycids species captured using multi‐funnel traps baited with a multi‐lure blend of longhorn beetle pheromones and host volatiles, an increasingly important tool for the early detection of non‐native species (Dodds et al. 2024; Santoiemma et al. 2024). Here, we address this gap by assembling a curated DNA barcode reference library for Cerambycidae species collected across Europe using standardized multi‐pheromone trapping protocols, and by evaluating specimen identification performance using both distance‐based metrics and monophyly‐based approaches. This dataset is designed to enhance the speed, accuracy, and scalability of cerambycid identifications in a biosurveillance context. The reference library is built from the list of Cerambycidae species captured during a large‐scale European trapping survey, meaning that species inclusion is based on detection in that study rather than on their native or overall geographic distribution. Consequently, the database includes taxa that may occur outside Europe but are relevant to European biosurveillance because they have already been recorded in trapping campaigns conducted within Europe. By emphasizing taxonomic validation and data quality, our library provides a robust and reliable tool for identifying Cerambycidae specimens collected in baited traps with a multi‐lure blend of longhorn beetle pheromones and host volatiles in European generic surveillance programs (Santoiemma et al. 2024).

## 2 Material and Methods

### 2.1 Taxa Sampling

We focused on the 185 species of Cerambycidae recorded by Roques et al. (2023) as being captured in Europe using multi‐pheromone traps (Table 1).

**TABLE 1:** Taxonomy, origin, and number of barcodes (4097 in total) in our local database for the 185 target species included in this study.

Subfamily	Tribe	Species	Origin	Nbarcodes
Cerambycinae	Anaglyptini	*Anaglyptus gibbosus*	Europe	1
Cerambycinae	Anaglyptini	*Anaglyptus mysticus*	Europe	26
Cerambycinae	Callichromatini	*Aromia moschata*	Europe	27
Cerambycinae	Graciliini	*Axinopalpis gracilis*	Europe	3
Cerambycinae	Callidiini	*Callidium aeneum*	Holarctic	10
Cerambycinae	Callidiini	*Callidium violaceum*	Europe	61
Cerambycinae	Stenopterini	*Callimus abdominalis*	Europe	1
Cerambycinae	Stenopterini	*Callimus angulatus*	Europe	8
Cerambycinae	Cerambycini	*Cerambyx cerdo*	Europe	18
Cerambycinae	Cerambycini	*Cerambyx miles*	Europe	4
Cerambycinae	Cerambycini	*Cerambyx scopolii*	Europe	38
Cerambycinae	Cerambycini	*Cerambyx welensii*	Europe	9
Cerambycinae	Certallini	*Certallum ebulinum*	Europe	3
Cerambycinae	Clytini	*Chlorophorus figuratus*	Europe	38
Cerambycinae	Clytini	*Chlorophorus glabromaculatus*	Europe	10
Cerambycinae	Clytini	*Chlorophorus glaucus*	Europe	1
Cerambycinae	Clytini	*Chlorophorus herbstii*	Europe	3
Cerambycinae	Clytini	*Chlorophorus ruficornis*	Europe	12
Cerambycinae	Clytini	*Chlorophorus sartor*	Europe	18
Cerambycinae	Clytini	*Chlorophorus trifasciatus*	Europe	5
Cerambycinae	Clytini	*Chlorophorus varius*	Europe	6
Cerambycinae	Clytini	*Clytus arietis*	Europe	96
Cerambycinae	Clytini	*Clytus lama*	Europe	24
Cerambycinae	Clytini	*Clytus rhamni*	Europe	43
Cerambycinae	Clytini	*Clytus tropicus*	Europe	3
Cerambycinae	Phoracanthini	*Cordylomera spinicornis*	Africa	1
Cerambycinae	Deilini	*Deilus fugax*	Europe	16
Cerambycinae	Molorchini	*Dolocerus reichii*	Europe	0
Cerambycinae	Graciliini	*Gracilia minuta*	Europe	6
Cerambycinae	Hesperophanini	*Hesperophanes sericeus*	Europe	3
Cerambycinae	Hylotrupini	*Hylotrupes bajulus*	Europe	26
Cerambycinae	Clytini	*Isotomus speciosus*	Europe	0
Cerambycinae	Callidiini	*Lioderina linearis*	Europe	0
Cerambycinae	Molorchini	*Molorchus minor*	Europe	70
Cerambycinae	Molorchini	*Molorchus umbellatarum*	Europe	28
Cerambycinae	Nathriini	*Nathrius brevipennis*	Europe	14
Cerambycinae	Obriini	*Obrium brunneum*	Europe	21
Cerambycinae	Obriini	*Obrium cantharinum*	Europe	2
Cerambycinae	Graciliini	*Penichroa fasciata*	Europe	9
Cerambycinae	Phoracanthini	*Phoracantha recurva*	Australasia	13
Cerambycinae	Phoracanthini	*Phoracantha semipunctata*	Australasia	14
Cerambycinae	Callidiini	*Phymatodes alni*	Europe	38
Cerambycinae	Callidiini	*Phymatodes fasciatus*	Europe	4
Cerambycinae	Callidiini	*Phymatodes glabratus*	Europe	8
Cerambycinae	Callidiini	*Phymatodes lividus*	Europe	1
Cerambycinae	Callidiini	*Phymatodes pusillus*	Europe	7
Cerambycinae	Callidiini	*Phymatodes rufipes*	Europe	11
Cerambycinae	Callidiini	*Phymatodes testaceus*	Europe	93
Cerambycinae	Clytini	*Plagionotus arcuatus*	Europe	21
Cerambycinae	Clytini	*Plagionotus detritus*	Europe	6
Cerambycinae	Clytini	*Pseudosphegesthes cinerea*	Europe	3
Cerambycinae	Trachyderini	*Purpuricenus budensis*	Europe	14
Cerambycinae	Trachyderini	*Purpuricenus globulicollis*	Europe	2
Cerambycinae	Trachyderini	*Purpuricenus kaehleri*	Europe	31
Cerambycinae	Callidiini	*Pyrrhidium sanguineum*	Europe	14
Cerambycinae	Callidiini	*Ropalopus clavipes*	Europe	6
Cerambycinae	Callidiini	*Ropalopus femoratus*	Europe	4
Cerambycinae	Callidiini	*Ropalopus macropus*	Europe	1
Cerambycinae	Callidiini	*Ropalopus varini*	Europe	4
Cerambycinae	Stenopterini	*Stenopterus ater*	Europe	13
Cerambycinae	Stenopterini	*Stenopterus rufus*	Europe	66
Cerambycinae	Hesperophanini	*Stromatium auratum*	Europe	0
Cerambycinae	Hesperophanini	*Trichoferus campestris*	Asia	171
Cerambycinae	Hesperophanini	*Trichoferus fasciculatus*	Europe	7
Cerambycinae	Hesperophanini	*Trichoferus holosericeus*	Europe	8
Cerambycinae	Hesperophanini	*Trichoferus pallidus*	Europe	5
Cerambycinae	Clytini	*Xylotrechus antilope*	Europe	74
Cerambycinae	Clytini	*Xylotrechus arvicola*	Europe	12
Cerambycinae	Clytini	*Xylotrechus chinensis*	Asia	6
Cerambycinae	Clytini	*Xylotrechus pantherinus*	Europe	3
Cerambycinae	Clytini	*Xylotrechus rusticus*	Europe/Asia	31
Cerambycinae	Clytini	*Xylotrechus stebbingi*	Asia	38
Lamiinae	Acanthocinini	*Acanthocinus aedilis*	Europe/Asia	30
Lamiinae	Acanthocinini	*Acanthocinus griseus*	Europe/Asia	16
Lamiinae	Acanthoderini	*Aegomorphus clavipes*	Europe	15
Lamiinae	Acanthoderini	*Aegomorphus francottei*	Europe	1
Lamiinae	Acanthoderini	*Aegomorphus krueperi*	Europe	8
Lamiinae	Agapanthiini	*Agapanthia cardui*	Europe	46
Lamiinae	Agapanthiini	*Agapanthia villosoviridescens*	Europe	41
Lamiinae	Apodasyini	*Anaesthetis testacea*	Europe	16
Lamiinae	Apodasyini	*Deroplia genei*	Europe	2
Lamiinae	Apodasyini	*Deroplia troberti*	Europe	2
Lamiinae	Exocentrini	*Exocentrus adspersus*	Europe	48
Lamiinae	Exocentrini	*Exocentrus lusitanus*	Europe	21
Lamiinae	Exocentrini	*Exocentrus punctipennis*	Europe	11
Lamiinae	Acanthocinini	*Leiopus femoratus*	Europe	20
Lamiinae	Acanthocinini	*Leiopus linnei*	Europe	8
Lamiinae	Acanthocinini	*Leiopus nebulosus*	Europe	19
Lamiinae	Saperdini	*Menesia bipunctata*	Europe	6
Lamiinae	Mesosini	*Mesosa curculionoides*	Europe	6
Lamiinae	Mesosini	*Mesosa nebulosa*	Europe	18
Lamiinae	Monochamini	*Monochamus galloprovincialis*	Europe/Asia	44
Lamiinae	Monochamini	*Monochamus saltuarius*	Asia/Europe	9
Lamiinae	Monochamini	*Monochamus sartor*	Europe	16
Lamiinae	Monochamini	*Monochamus sutor*	Europe/Asia	34
Lamiinae	Pteropliini	*Niphona picticornis*	Europe	4
Lamiinae	Phytoeciini	*Oberea linearis*	Europe	11
Lamiinae	Acanthoderini	*Oplosia cinerea*	Europe	5
Lamiinae	Parmenini	*Parmena balteus*	Europe	4
Lamiinae	Parmenini	*Parmena unifasciata*	Europe	0
Lamiinae	Phytoeciini	*Phytoecia nigricornis*	Europe	15
Lamiinae	Phytoeciini	*Phytoecia pustulata*	Europe	19
Lamiinae	Pogonocherini	*Pogonocherus caroli*	Europe	5
Lamiinae	Pogonocherini	*Pogonocherus decoratus*	Europe	13
Lamiinae	Pogonocherini	*Pogonocherus fasciculatus*	Europe	15
Lamiinae	Pogonocherini	*Pogonocherus hispidulus*	Europe	7
Lamiinae	Pogonocherini	*Pogonocherus hispidus*	Europe	35
Lamiinae	Pogonocherini	*Pogonocherus ovatus*	Europe	1
Lamiinae	Pogonocherini	*Pogonocherus perroudi*	Europe	5
Lamiinae	Saperdini	*Saperda octopunctata*	Europe	3
Lamiinae	Saperdini	*Saperda perforata*	Europe	11
Lamiinae	Saperdini	*Saperda populnea*	Europe	13
Lamiinae	Saperdini	*Saperda scalaris*	Europe	32
Lamiinae	Saperdini	*Stenostola dubia*	Europe	18
Lamiinae	Saperdini	*Stenostola ferrea*	Europe	4
Lepturinae	Lepturini	*Alosterna tabacicolor*	Europe	71
Lepturinae	Rhagiini	*Acmaeops marginatus*	Europe/Asia	0
Lepturinae	Rhagiini	*Acmaeops pratensis*	Europe	0
Lepturinae	Rhagiini	*Acmaeops septentrionis*	Europe/Asia	0
Lepturinae	Rhagiini	*Acmaeops smaragdulus*	Europe	0
Lepturinae	Lepturini	*Anastrangalia dubia*	Europe	64
Lepturinae	Lepturini	*Anastrangalia reyi*	Europe	23
Lepturinae	Lepturini	*Anastrangalia sanguinolenta*	Europe	134
Lepturinae	Rhagiini	*Anisorus quercus*	Europe	0
Lepturinae	Lepturini	*Anoplodera rufipes*	Europe	8
Lepturinae	Lepturini	*Anoplodera sexguttata*	Europe	26
Lepturinae	Rhagiini	*Brachyta interrogationis*	Europe	25
Lepturinae	Rhagiini	*Carilia virginea*	Europe	0
Lepturinae	Rhagiini	*Cortodera femorata*	Europe	14
Lepturinae	Rhagiini	*Cortodera flavimana*	Europe	0
Lepturinae	Rhagiini	*Cortodera humeralis*	Europe	18
Lepturinae	Rhagiini	*Dinoptera collaris*	Europe	59
Lepturinae	Rhagiini	*Grammoptera abdominalis*	Europe	11
Lepturinae	Rhagiini	*Grammoptera ruficornis*	Europe	62
Lepturinae	Rhagiini	*Grammoptera ustulata*	Europe	27
Lepturinae	Lepturini	*Leptura aethiops*	Europe	3
Lepturinae	Lepturini	*Leptura aurulenta*	Europe	12
Lepturinae	Lepturini	*Leptura quadrifasciata*	Europe	36
Lepturinae	Oxymirini	*Oxymirus cursor*	Europe	11
Lepturinae	Lepturini	*Pachytodes erraticus*	Europe	34
Lepturinae	Lepturini	*Pedostrangalia revestita*	Europe	5
Lepturinae	Rhagiini	*Pidonia lurida*	Europe	24
Lepturinae	Lepturini	*Pseudovadonia livida*	Europe	90
Lepturinae	Rhagiini	*Rhagium bifasciatum*	Europe	19
Lepturinae	Rhagiini	*Rhagium inquisitor*	Holarctic	149
Lepturinae	Rhagiini	*Rhagium mordax*	Europe	44
Lepturinae	Rhagiini	*Rhagium sycophanta*	Europe	34
Lepturinae	Lepturini	*Rutpela maculata*	Europe	116
Lepturinae	Rhagiini	*Stenocorus meridianus*	Europe	18
Lepturinae	Lepturini	*Stenurella bifasciata*	Europe	40
Lepturinae	Lepturini	*Stenurella melanura*	Europe	194
Lepturinae	Lepturini	*Stenurella nigra*	Europe	48
Lepturinae	Lepturini	*Stenurella septempunctata*	Europe	8
Lepturinae	Lepturini	*Stictoleptura cordigera*	Europe	35
Lepturinae	Lepturini	*Stictoleptura erythroptera*	Europe	1
Lepturinae	Lepturini	*Stictoleptura fontenayi*	Europe	1
Lepturinae	Lepturini	*Stictoleptura fulva*	Europe	0
Lepturinae	Lepturini	*Stictoleptura hybrida*	Europe	0
Lepturinae	Lepturini	*Stictoleptura maculicornis*	Europe	43
Lepturinae	Lepturini	*Stictoleptura rubra*	Europe/Asia	72
Lepturinae	Lepturini	*Stictoleptura scutellata*	Europe	22
Lepturinae	Lepturini	*Stictoleptura trisignata*	Europe	7
Lepturinae	Lepturini	*Strangalia attenuata*	Europe	6
Lepturinae	Lepturini	*Vadonia unipunctata*	Europe	51
Necydalinae	Necydalini	*Necydalis major*	Europe	6
Necydalinae	Necydalini	*Necydalis ulmi*	Europe	3
Prioninae	Aegosomatini	*Aegosoma scabricorne*	Europe	5
Prioninae	Prionini	*Mesoprionus besikanus*	Europe	3
Prioninae	Macrotomini	*Prinobius myardi*	Europe	26
Prioninae	Prionini	*Prionus coriarius*	Europe	16
Spondylidinae	Anisarthrini	*Alocerus moesiacus*	Europe	1
Spondylidinae	Anisarthrini	*Anisarthron barbipes*	Europe	0
Spondylidinae	Asemini	*Arhopalus ferus*	Europe	24
Spondylidinae	Asemini	*Arhopalus rusticus*	Europe/Asia	52
Spondylidinae	Asemini	*Asemum striatum*	Holarctic	46
Spondylidinae	Asemini	*Asemum tenuicorne*	Europe	0
Spondylidinae	Asemini	*Cephalocrius syriacus*	Europe	14
Spondylidinae	Nothorhinini	*Nothorhina punctata*	Europe	1
Spondylidinae	Saphanini	*Oxypleurus nodieri*	Europe	2
Spondylidinae	Spondylidini	*Spondylis buprestoides*	Europe	61
Spondylidinae	Asemini	*Tetropium castaneum*	Europe	21
Spondylidinae	Asemini	*Tetropium fuscum*	Europe	13
Spondylidinae	Asemini	*Tetropium gabrieli*	Europe	2
Spondylidinae	Tetropiini	*Tetrops praeustus*	Europe	72
Spondylidinae	Tetropiini	*Tetrops starkii*	Europe	14

*Note:* These species correspond to cerambycids captured (excluding Vesperidae and Disteniidae) with multi‐pheromone traps across European territory (Roques et al. 2023).

Barcode data were compiled from four sources: (1) specimens collected across Europe using traps with a multi‐pheromone blend, designed to attract simultaneously a broad range of cerambycid species from different subfamilies and tribes (Fan et al. 2019; Hanks et al. 2012; Roques et al. 2023) (BOLD projects PHDLV, BARBC, CERAC). Specimens were first identified using morphological criteria prior to DNA barcoding, with identifications performed by Alain Roques and Guilhem Parmain. (2) specimens stored at INRAe‐CBGP, Continental Arthropod Collection (Centre de Biologie pour la Gestion des Populations, Montpellier, France; https://doi.org/10.15454/D6XAKL) (BOLD projects CBGP, CICRP). Specimens were primarily collected and morphologically identified by Cerambycidae taxonomists Christian Cocquempot and Hervé Brustel, using complementary methods including diurnal netting on flowers, deadwood surveys, and light trapping; and (3) specimens deposited at the Museum national d'Histoire naturelle, Paris, collected and identified by Cerambycidae taxonomic expert Julien Touroult (BOLD project CLPFR), and (4) publicly available barcodes retrieved from over 70 different projects (see Table S1—Taxonomy sheet) in the BOLD Systems database, which were used to complete the intraspecific sampling. Details on the identifiers of each record and the methods used for identification are provided in Table S1 (taxonomy sheet).

### 2.2 Laboratory Procedures and Sequencing

Individuals barcoded as part of the Roques et al. (2023) study were stored at −20°C in 95% ethanol before undergoing species‐level identification. Once morphologically identified, the individuals were photographed, and the associated metadata was entered into the BOLD database. A tissue sample was then taken from each specimen (one or two legs, depending on the size of the individuals), and DNA was extracted using the DNeasy Blood & Tissue Kit (Qiagen) or Genomic DNA from Tissue Kit (Macherey‐Nagel) following the manufacturer's recommendations. DNA amplification targeted the mitochondrial COI barcode region using the primers LCO1490 and HCO2198 (Folmer et al. 1994). Specimens were either barcoded through Sanger sequencing on an ABI 3500 Genetic Analyzer (Applied Biosystems) using the BigDye Terminator v3.1 Cycle Sequencing Kit (Applied Biosystems) at URZF in Orléans, or were sent to the Canadian Centre for Biodiversity Genomics (CCBG) at the Biodiversity Institute of Ontario (http://ibol.org) at the University of Guelph, Canada. For the latter, DNA extraction, PCR amplification, and sequencing were carried out using single molecule real‐time (SMRT) processing on the PacBio Sequel sequencing platform (Pacific Biosciences, Menlo Parc—California, United States of America) at the Canadian Centre for DNA Barcoding (CCDB), University of Guelph, following their standard high‐throughput DNA barcoding pipeline for arthropods (Ivanova et al. 2006; deWaard et al. 2008; Ratnasingham and Hebert 2007).

For the individuals barcoded at CBGP, specimens were identified morphologically and preserved in 80%–95% EtOH until DNA extraction. DNA was extracted from legs using the Qiagen DNeasy kit (Hilden, Germany) according to the manufacturer's protocol. COI was amplified using a cocktail of M13‐tailed LCO1490 and HCO2198 primers. Primer sequences, as well as details on the reaction mix and PCR conditions, are available in Germain et al. (2013). Unpurified PCR products were submitted to Eurofins MWG Operon (Ebersberg, Germany) for sequencing.

### 2.3 Dataset Construction and Quality Filtering

In addition to our newly generated barcodes, we retrieved from BOLD publicly available sequences of the COI‐5P marker (5′ end of the COI gene which corresponds to the barcode fragment) by mining the database using the taxonomic filter (species: list of the 185 species of Cerambycidae (Roques et al. 2023)). These sequences originated from over 70 different projects in BOLD (see Table S1, lab sheet, project code). We only retained sequences whose length was comprised between 500 and 658 bp. The amino acid sequences containing stop codons were excluded. The remaining sequences were aligned with the BOLD Aligner, the alignment algorithm integrated within BOLD. All DNA barcode records are publicly available in the BOLD data set “DS‐EUROCERA”.

### 2.4 Analyses of DNA Barcodes

To assess intraspecific variation, we used the Python script aln_hapcounter.py from the PyCOIstats package to calculate the number of distinct haplotypes for each species in the final database. Haplotypes containing ambiguous bases (Y, W, N, etc.), missing data, or gaps were excluded to improve downstream computation times. Haplotype calculations were performed on the alignment of all sequences in our local BOLD database using the “BOLD aligner” option in BOLD v4, based on an Amino Acid Hidden Markov Model. Additionally, pairwise distances were calculated from the same alignment file using the aln_pdistancer.py script from the PyCOIstats v1.3 package, which enabled the calculation of maximum intraspecific distances (intra_*d*max) and the minimum distance to the nearest neighbor (*D*min_NN) (Lopez‐Vaamonde et al. 2021).

To detect potential cases of non‐monophyly, we generated a maximum likelihood tree using IQ‐Tree v2.1.4‐beta (Minh et al. 2020) under the GTR model. Statistical support of nodes was evaluated with 100 standard bootstrap replications. Phylogenetic analyses were run on the Genotoul bioinformatics platform (INRAE, Toulouse, France). The resulting tree was then analyzed using the script Tree_monophyly.py from the PyCOIstats package (Doorenweerd 2021), which uses the monophyly() function from Biopython (Cock et al. 2009). The script takes a newick‐formatted tree along with a CSV of taxa names to be tested for monophyly. All cases of non‐monophyly were manually reviewed to assess the likely cause (e.g., misidentification, barcode sharing, introgression, operational error, heteroplasmy, sequencing of coding pseudogenes). Sequences identified as operational errors, arising from sample processing, sequence generation, or database entry (e.g., misidentification, contamination, sequencing, or data entry errors) based on phylogenetic tree analysis were subsequently removed. A second round of analyses was performed using IQ‐Tree and Monophylizer (https://github.com/naturalis/monophylizer) to ensure persistent non‐monophyly cases due to operational errors were all resolved. The final phylogenetic tree is provided in Figure S1 and can be downloaded in Newick format.

## 3 Results

### 3.1 Taxonomic Coverage

The final dataset contains 4097 COI‐5P sequences longer than 500 bp, of which 3153 sequences (76.96%) cover the full barcode fragment of 658 bp. These sequences correspond to 169 species of Cerambycids out of the 185 target species captured in Europe using multi‐pheromonal traps (Roques et al. 2023), representing 91.35%. (Table 1). Of the total, 2359 public sequences were retrieved from the BOLD database, 1586 were generated at CBGP, and 152 came from individuals collected in the study by Roques et al. (2023). The dataset spans 37 different localities, with France contributing the largest share (1689 barcodes), followed by Germany (872 sequences) and Finland (203 sequences). On average, each species is represented by 24 individuals (SD = 30.73), ranging from singletons (12 species) to a maximum of 194 individuals for *Stenurella melanura*. Sixteen species (8.65%) remain absent in the dataset because the species are not yet barcoded or the existing barcodes did not pass the quality filters applied in this study.

### 3.2 Haplotype Analysis

**FIGURE 1:**
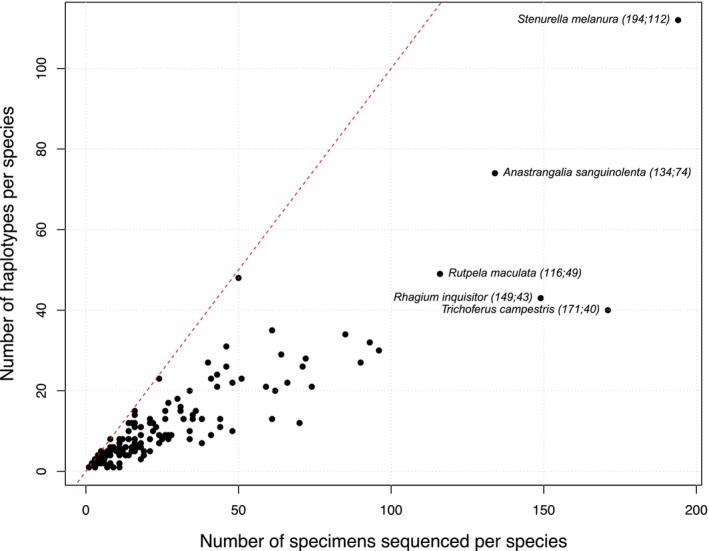
Relationships between haplotype richness and sampling effort. Total Number of distinct haplotypes plotted against the number of specimens sequenced by species. In total 1786 haplotypes were identified from 4097 sequences. The five most represented cerambycids are highlighted in the graph with species name species followed by the number of specimens/sequences and corresponding haplotype counts.

The dataset contains 1786 distinct haplotypes, with up to 112 haplotypes identified among the 194 individuals of *Stenurella melanura*. On average, each species harbors 10.6 haplotypes (SD = 13.33) (Table S2). Haplotype diversity is strongly correlated with sampling effort (Pearson correlation: *r* = 0.8937, *N* = 169, *p* < 0.05) (Figure 1).

### 3.3 DNA Barcoding Performance

#### 3.3.1 Phylogeny

Monophyly assessment using Monophylizer identified 28 species with non‐monophyletic sequences. After morphological examination by Christian Cocquempot to confirm species identity of voucher specimens, 87 problematic sequences were removed as likely contaminations or misidentifications. A subsequent analysis revealed only 13 non‐monophyletic species out of 169 (7.69%): (*Purpuricenus kaehleri, Purpuricenus budensis, Chlorophorus ruficornis, Chlorophorus sartor*, *Phoracantha semipunctata* , *Monochamus sutor, Monochamus galloprovincialis, Monochamus sartor, Leiopus linnei, Leiopus nebulosus, Anastrangalia dubia, Anastrangalia reyi, Pachytodes erraticus*).

#### 3.3.2 Bin Discordance Tool

In total, 273 distinct BINS were retrieved (Table S3). Of these, 222 (81.32%) were concordant with operational taxonomic units and morphological species delimitations established through traditional taxonomic keys (Berger 2012; Sama 2002, 2023; Villiers 1978) and confirmed by a Cerambycidae taxonomist expert Christian Cocquempot. Seven BINS (2.56%) were discordant, including: BOLD:AAC3388: *Monochamus sutor*/*Monochamus sartor*; BOLD:AAX4737: *Monochamus sutor*/*Monochamus sartor*; BOLD:AEJ2342: *Purpuricenus kaehleri*/*Purpuricenus budensis*; BOLD:ABX1273: *Chlorophorus ruficornis*/*Chlorophorus sartor*; BOLD:AEI9961: *Purpuricenus kaehleri*/*Purpuricenus budensis*; BOLD:AAJ2081: *Anastrangalia reyi*/*Anastrangalia dubia*; BOLD:AAJ1830: *Leiopus nebulosus*/*Leiopus linnei*. Additionally, 44 singleton BINS (16.11%) contained only a single individual, preventing assessment of concordance or discordance (Table S3). Across species, 74 (43.79%) were associated with more than one BIN, ranging from two up to eight BINs in *Rhagium inquisitor* , including 13 species with at least one unidentified BIN (Table S3). By contrast, 106 species (62.72%) were assigned to a single BIN, and only one species, *Cordylomera spinicornis*, lacked any BIN assignment.

#### 3.3.3 Distance Based Approach

**FIGURE 2:**
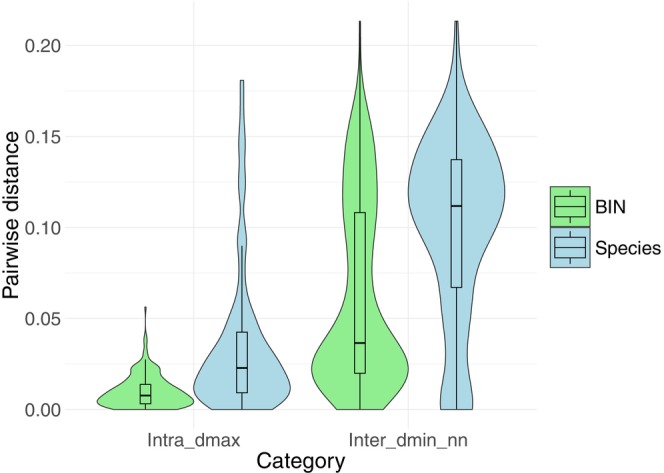
Violin plot of pairwise distances within and between species and BINs. For each violin, the central boxplot shows the interquartile range (first and third quartiles) with median indicated by central dot. “*D*max” represents the maximum infraspecific distance, while “*D*min_NN” represents the minimum distance to the nearest neighbor. Violin width is normalized to the same maximum width across categories, allowing direct comparison of the distributions regardless of density differences.

We assessed the reliability of DNA barcodes by comparing maximum intraspecific distances (*D*max) with the minimum interspecific distances to the nearest neighbor (*D*min_NN) (Table S4). In both species‐level and BIN‐level analyses, *D*max was consistently lower than *D*min_NN, supporting effective species discrimination (Figure 2). However, species defined by traditional taxonomy showed higher maximum intraspecific distances (up to 17.96%) compared with BIN‐defined groups (up to 5.62%). On average, interspecific distances were 10.59% for species and 6.15% for BINs.

### 3.4 Distance Anomalies

We identified 19 species (11.24%) with potential identification challenges due to low interspecific divergence (Table 2): These include one species triplet (*Monochamus* spp.), several species pairs (*Anastrangalia* spp., *Chlorophorus* spp., *Leiopus* spp., *Purpuricenus* spp.) and a set of singletons: *Agapanthia cardui, Cortodera femorata, Pachytodes erraticus*, *Phoracantha semipunctata* , *Phytoecia nigricornis*, *Rhagium inquisitor* , *Tetropium castaneum* , *Tetrops praeustus*. Among them, 11 species showed a *D*min_NN of less than 2%. The remaining eight species had *D*min_NN values greater than 2% but still lower than their respective *D*max, suggesting possible barcode overlap or cryptic speciation (Table 2).

**TABLE 2:** List of species within our database with the nearest neighbor is less than 2% divergent or when the distance to the nearest neighbor is less than the maximum intra‐specific distance, along with their monophyly status.

Species	Max intra‐Sp	Nearest neighbor	Distance to NN	Monophyly status
*Agapanthia cardui*	14.22	*Agapanthia villosoviridescens*	14.02	Monophyletic
*Anastrangalia dubia*	1.55	*Anastrangalia reyi*	0	Non‐monophyletic
*Anastrangalia reyi*	1.87	*Anastrangalia dubia*	0	Non‐monophyletic
*Chlorophorus ruficornis*	1.24	*Chlorophorus sartor*	0	Non‐monophyletic
*Chlorophorus sartor*	2.03	*Chlorophorus ruficornis*	0	Non‐monophyletic
*Cortodera femorata*	10.17	*Cortodera humeralis*	9.21	Monophyletic
*Leiopus linnei*	2.83	*Leiopus nebulosus*	0	Non‐monophyletic
*Leiopus nebulosus*	13.05	*Leiopus linnei*	0	Non‐monophyletic
*Monochamus galloprovincialis*	1.97	*Monochamus sartor*	1.73	Non‐monophyletic
*Monochamus sartor*	4.95	*Monochamus sutor*	0	Non‐monophyletic
*Monochamus sutor*	5.46	*Monochamus sartor*	0	Non‐monophyletic
*Pachytodes erraticus*	20.91	*Dinoptera collaris*	14.43	Non‐monophyletic
*Phoracantha semipunctata*	16.31	*Phoracantha recurva*	13.26	Non‐monophyletic
*Phytoecia nigricornis*	6.08	*Phytoecia pustulata*	4.58	Monophyletic
*Purpuricenus budensis*	3	*Purpuricenus kaehleri*	0	Non‐monophyletic
*Purpuricenus kaehleri*	2.83	*Purpuricenus budensis*	0	Non‐monophyletic
*Rhagium inquisitor*	12.91	*Rhagium mordax*	9.05	Monophyletic
*Tetropium castaneum*	8.75	*Tetropium gabrieli*	8.54	Monophyletic
*Tetrops praeustus*	13.89	*Monochamus galloprovincialis*	11.7	Monophyletic

## 4 Discussion

Across the 169 sequenced species analyzed, we found a high rate of monophyly (~92% excluding singletons), confirming that the COI barcode fragment is generally effective for species‐level identification in Cerambycidae. Importantly, only biologically meaningful cases of non‐monophyly were retained, as 87 sequences (60%) suspected of operational errors were excluded during data curation. The proportion of non‐monophyly cases attributable to such errors in our dataset mirrors that reported in Lepidoptera by Mutanen et al. (2016), where they accounted for 58.6% of cases. These errors, common in public repositories, often stem from morphological misidentifications, sample contamination, or unresolved synonymy. Some of the non‐monophylies observed here have also been previously documented, such as the case of *Anastrangalia dubia* and *A. reyi* , known to share a BIN (Hendrich et al. 2015; Rougerie et al. 2015).

Beyond operational issues, true biological processes can also contribute to barcode ambiguity. NUMTs (nuclear mitochondrial pseudogenes) are an unavoidable challenge in COI‐based barcoding. In our study, we applied a two‐step approach to minimize their influence: first, sequences containing stop codons were excluded during filtering, resulting in the removal of 11 sequences; second, the phylogenetic tree generated for all sequences (Figure S1) was systematically inspected during manual curation, as NUMT‐derived sequences typically produce distinctive topological signatures such as unexpectedly long branches or aberrant clustering with distantly related taxa. When such patterns were observed, the sequences concerned were flagged and removed. We acknowledge, however, that this approach does not guarantee complete NUMT elimination (Haran et al. 2015; Bertheau et al. 2011), and that some NUMTs may still be present in the final dataset. For future studies, we recommend that sequences suspected of being NUMTs be subjected to dedicated phylogenetic analysis, as tree topology provides a more objective and reliable basis for this judgment than sequence composition alone (Song et al. 2008).

Another potential source of non‐monophyly is introgression, where hybridization between closely related species results in mitochondrial transfer across species boundaries (Suvorov et al. 2020). Mitochondrial introgression, estimated to affect ca 10% of animal species, has been shown to obscure species boundaries in barcode datasets (Flouri et al. 2020). In Cerambycidae, examples of introgression were documented between *Cerambyx cerdo* and *Cerambyx welensii*, (Torres‐Vila and Bonal 2019) and between flightless *Dorcadion* species, which exhibit extensive mito‐nuclear discordance (Dascălu et al. 2022; Caba and Dascălu 2024). They were also presumed between *Anoplophora glabripennis* and *Anoplophora chinensis* (Wang and Keena 2021). It is important to note, however, that introgression is not limited to cases exhibiting discordance or non‐monophyly. Mitochondrial introgression can occur silently, leaving no detectable signature in COI‐based analyses when introgressed haplotypes happen to cluster within species boundaries defined by morphology (Funk and Omland 2003; Yuan et al. 2023). Consequently, species appearing monophyletic in our dataset cannot be assumed to be free of introgression. Resolving this ambiguity would require the integration of nuclear markers, as mito‐nuclear discordance can only be detected through comparison of mitochondrial and nuclear genealogies (Toews and Brelsford 2012).

Species delimitation in certain groups of Cerambycidae remains challenging due to high morphological similarity and cryptic diversity. In our study, 19 species showed potential identification challenges when using distance‐based approaches: 11 with low interspecific COI divergence (< 2%) and eight with divergences above 2% but still lower than their maximum intraspecific distance (Table 2). Our comparative analysis between distance‐based and topology‐based methods revealed that while topological approaches examining monophyly identified 13 non‐monophyletic species, all of these were encompassed within the 19 problematic species detected through distance‐based methods. This complete overlap suggests that distance‐based methods may sometimes detect additional cases not flagged by monophyly assessment alone, although this could also reflect an inappropriate distance threshold for certain species. Such cases highlight a well‐known limitation of COI barcoding: low divergence can lead to shared BINs or poorly delimited sequence clusters in BOLD, with sequences from different species grouped in the same BIN, or a single BIN containing multiple species names. The additional ambiguous cases detected only through distance methods require further investigation to clarify whether they represent true cryptic species, intraspecific geographic structure, or methodological artifacts. Resolving these taxonomic ambiguities will require integrative approaches combining both distance‐ and topology‐based analyses with nuclear markers, multi‐locus datasets, species delimitation methods, and complementary morphological or ecological evidence, providing the most robust framework for accurate species delimitation in morphologically challenging groups such as some longhorn beetles.

A key question raised by these cases is whether species that cannot be reliably separated using COI truly represent distinct biological entities. For taxa showing near‐zero interspecific divergence, such as *Anastrangalia dubia/reyi*, *Chlorophorus ruficornis/sartor*, *Leiopus linnei/nebulosus*, and *Purpuricenus kaehleri/budensis*, two non‐exclusive hypotheses can be put forward. First, these taxa may represent genuinely distinct species that have diverged too recently for COI to accumulate sufficient diagnostic variation; a phenomenon known as incomplete lineage sorting (Funk and Omland 2003). Second, they may represent cases of taxonomic over‐splitting, where morphologically defined species boundaries do not correspond to independently evolving lineages. In our study, we did not apply formal species delimitation methods such as ASAP (Puillandre et al. 2021) or GMYC (Pons et al. 2006), as our approach is based on voucher specimens examined morphologically, allowing us to ground species hypotheses in established taxonomic practice. We adopted an integrative approach in which specimens were first identified using morphology, and these initial hypotheses were then re‐evaluated using DNA barcoding through both distance‐based and topology‐based frameworks. Resolving the aforementioned hypotheses would require going beyond this approach, through the application of such formal species delimitation methods, combined with nuclear markers and complementary morphological or ecological evidence. For taxa with *D*min_NN above 2% but still lower than their maximum intraspecific distance, such as *Phytoecia nigricornis*, *Tetropium castaneum* , or *Tetrops praeustus*, the situation is different: the observed pattern may reflect deep intraspecific geographic structure rather than species boundary ambiguity, though cryptic speciation cannot be excluded without further investigation. In a biosurveillance context, these uncertainties have direct practical consequences, as the inability to distinguish between closely related species using COI alone could lead to misidentification of intercepted specimens and inappropriate management responses.

Beyond species delimitation challenges, high haplotype diversity was also observed in several species, most notably in *Stenurella melanura* with 112 haplotypes identified among 194 individuals (Figure 1). Specimens of *Stenu*r*ella melanura* were collected from 11 countries (Austria, Germany, Spain, France, Belgium, Norway, Poland, United Kingdom, Croatia, Italy, and Finland) and assigned to six distinct BINs (Table S3), indicating substantial genetic differentiation within the taxon. This level of intraspecific variability far exceeds the dataset average of 10.57 haplotypes per species, suggesting that *S. melanura* may not represent a single homogeneous species. Possible explanations include pronounced population structuring or the existence of a complex of closely related, cryptic species. Indeed, several synonymized species within *S. melanura* have been noted and require reassessment (Zamoroka et al. 2022). Comparable patterns of high haplotype diversity have been reported in other taxa where morphologically defined species encompass genetically divergent lineages, underscoring that apparent intraspecific variation may often reflect hidden taxonomic complexity rather than simple population‐level diversity (Lopez‐Vaamonde et al. 2021). Nevertheless, abnormally high numbers of haplotypes can also result from the amplification of nuclear mitochondrial pseudogenes (NUMTs), as documented in *M. galloprovincialis* (Koutroumpa et al. 2009; Haran et al. 2015) and numerous other species, highlighting the need for careful verification of sequence authenticity when interpreting patterns of genetic diversity.

A broader limitation inherent to COI‐based barcoding deserves explicit discussion here. The COI gene was originally selected as a universal barcode marker precisely because of its relatively fast evolutionary rate, making it effective at capturing lineage differentiation at and below the species level (Hebert et al. 2003). However, this same property makes it inherently prone to oversplitting: COI can detect intraspecific genetic structure (driven by geographic isolation, demographic history, or drift) that does not necessarily correspond to true species boundaries (Moritz and Cicero 2004). In other words, COI is optimized for resolving lineages within species, and this sensitivity can lead to the artificial inflation of species diversity when applied to species delimitation. In our dataset, this limitation is particularly apparent in taxa such as *Stenurella melanura*, assigned to six distinct BINs across eleven countries, or *Rhagium inquisitor* , associated with up to eight BINs, where the observed genetic partitioning may reflect deep intraspecific geographic structure rather than genuine cryptic speciation. More generally, the 74 species (43.79%) in our dataset associated with more than one BIN should be interpreted with caution, as BIN multiplicity does not necessarily imply the existence of multiple biological species. These patterns highlight a well‐known paradox of COI barcoding: while the marker is powerful for distinguishing well‐differentiated species, it may simultaneously obscure the distinction between intraspecific variation and interspecific divergence in recently diverged or geographically structured taxa. Integrating additional markers, whether nuclear loci or whole‐genome approaches, remains the most reliable way to disentangle these confounded signals (Dupuis et al. 2012).

Beyond these conceptual limitations, practical gaps in taxonomic coverage also remain to be addressed. Sixteen cerambycid species (8.65%) are not yet represented in the database compiled in our study (Table 1). Their absence largely reflects limitations in specimen availability and the efficiency of pheromone trapping, particularly for rare taxa or those with poorly documented behaviors. Addressing these gaps will require targeted sampling of the missing species, as well as the integration of data from natural history collections (Levesque‐Beaudin et al. 2023) and/or dedicated field surveys. Expanding coverage of the database will ultimately increase the robustness of the reference library and enhance its value for ecological analyses and the early detection of non‐native species.

## 5 Conclusion

We developed a curated, high‐quality COI reference library for 169 Cerambycidae species captured with multi‐pheromone traps in Europe, combining newly generated sequences with carefully curated records from BOLD. Rigorous filtering and expert morphological species validation minimized taxonomic errors, resulting in high species discrimination based on paiwise distance and a monophyly rate of 92%.

This study highlights the essential role of manual curation in producing reference libraries for barcoding and metabarcoding. While labor‐intensive, such efforts are critical for accurate species identification, biosurveillance, and ecological research. Our dataset provides a robust foundation for the detection of both native and potentially invasive Cerambycidae species across Europe using metabarcoding approach (Veillat et al. 2024). The library sets benchmark for future high‐quality barcode libraries for the whole European fauna, with direct application in biosurveillance, pest monitoring, early detection of invasive species, and biodiversity assessment.

## Author Contributions

**Loïs Veillat:** conceptualization (supporting), data curation (equal), formal analysis (lead), investigation (lead), resources (equal), software (lead), validation (lead), visualization (lead), writing – original draft (lead), writing – review and editing (equal). **Christian Cocquempot:** data curation (equal), investigation (equal), resources (equal), writing – review and editing (equal). **Jean‐Claude Streito:** data curation (equal), investigation (equal), resources (equal), writing – review and editing (equal). **Éric Pierre:** data curation (equal), investigation (equal), resources (equal), writing – review and editing (equal). **Guénaëlle Genson:** data curation (equal), investigation (equal), resources (equal), writing – review and editing (equal). **Jean‐Yves Rasplus:** funding acquisition (equal), project administration (equal), resources (equal), writing – review and editing (equal). **Astrid Cruaud:** data curation (equal), resources (equal), writing – review and editing (equal). **Julien Touroult:** data curation (equal), investigation (equal), resources (equal), writing – review and editing (equal). **Alain Roques:** data curation (equal), funding acquisition (equal), investigation (equal), resources (equal), writing – review and editing (equal). **Béatrice Courtial:** data curation (equal), investigation (equal), resources (equal), writing – review and editing (equal). **Geraldine Roux:** conceptualization (equal), funding acquisition (equal), project administration (equal), supervision (equal), writing – review and editing (equal). **Carlos Lopez‐Vaamonde:** conceptualization (lead), data curation (lead), formal analysis (equal), funding acquisition (equal), investigation (equal), project administration (equal), resources (equal), supervision (lead), writing – review and editing (equal).

## Funding

This work was funded by the European Union's Horizon 2020 research and innovation programme under grant agreement No 771271 (project HOMED—HOlistic Management of Emerging forest pests and Diseases). This work was carried out as part of the Massif project and the PEPR FORESTT program, and received funding from the French State managed by the Agence Nationale de la Recherche under France 2030, bearing the reference ANR‐25‐PEFO‐0006.

## Conflicts of Interest

The authors declare no conflicts of interest.

## Supporting information


**Figure S1:** Phylogenetic tree based on 4097 COI gene fragments in our dataset. Node support for Maximum Likelihood (ML) inference is indicated by bootstrap values. The analysis was conducted with IQ‐TREE v2.1.4‐beta. Branches and labels in red indicate taxa that do not form monophyletic groups, as determined by topological assessment. Tip labels are formatted as Process_ID.Taxon_name.

**Data S1:** ece373626‐sup‐0002‐DataS1.xlsx.

**Data S2:** ece373626‐sup‐0003‐Supinfo.nwk.

## Data Availability

All data not reported in the manuscript are available in the Supporting Information. Final sequences, along with voucher metadata, images, and trace files, were deposited in BOLD (dataset ID: DS‐EUROCERA, DOI: http://doi.org/10.5883/DS‐EUROCERA) and subsequently in GenBank. All metadata associated with the 4097 sequences from our study are also available in Table S1.
