# Supplementary material for: From Traps to Taxa: Building a Robust DNA Barcode Reference Library for the Early Detection of Invasive Cerambycidae
Source: Ecol Evol. 2026 May 12;16(5):e73626. doi: 10.1002/ece3.73626 (PMC13167225; doi:10.1002/ece3.73626)
Supplement: Supplementary file 1 — Figure S1: Phylogenetic tree based on 4097 COI gene fragments in our dataset. Node support for Maximum Likelihood (ML) inference is indicated by bootstrap values. The analysis was conducted with IQ‐TREE v2.1.4‐beta. Branches and labels in red indicate taxa that do not form monophyletic groups, as determined by topological assessment. Tip labels are formatted as Process_ID.Taxon_name. [file ECE3-16-e73626-s003.pdf]

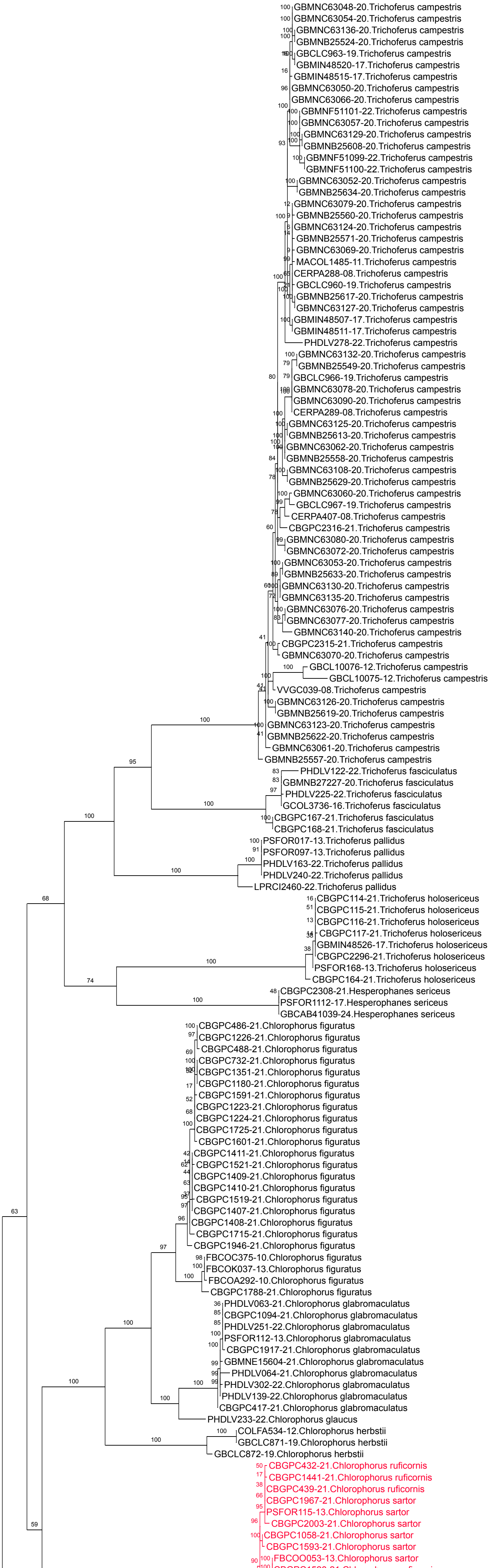
GBMNC63048-20.Trichoferus campestris
GBMNC63054-20.Trichoferus campestris
GBMNC63136-20.Trichoferus campestris
GBMNB25524-20.Trichoferus campestris
GBCLC963-19.Trichoferus campestris
GBMIN48520-17.Trichoferus campestris
GBMIN48515-17.Trichoferus campestris
GBMNC63050-20.Trichoferus campestris
GBMNC63066-20.Trichoferus campestris
GBMNF51101-22.Trichoferus campestris
GBMNC63057-20.Trichoferus campestris
GBMNC63129-20.Trichoferus campestris
GBMNB25608-20.Trichoferus campestris
GBMNF51099-22.Trichoferus campestris
GBMNF51100-22.Trichoferus campestris
GBMNC63052-20.Trichoferus campestris
GBMNB25634-20.Trichoferus campestris
GBMNC63079-20.Trichoferus campestris
GBMNB25560-20.Trichoferus campestris
GBMNC63124-20.Trichoferus campestris
GBMNB25571-20.Trichoferus campestris
GBMNC63069-20.Trichoferus campestris
MACOL1485-11.Trichoferus campestris
CERPA288-08.Trichoferus campestris
GBCLC960-19.Trichoferus campestris
GBMNB25617-20.Trichoferus campestris
GBMNC63127-20.Trichoferus campestris
GBMIN48507-17.Trichoferus campestris
GBMIN48511-17.Trichoferus campestris
PHDLV278-22.Trichoferus campestris
GBMNC63132-20.Trichoferus campestris
GBMNB25549-20.Trichoferus campestris
GBCLC966-19.Trichoferus campestris
GBMNC63078-20.Trichoferus campestris
GBMNC63090-20.Trichoferus campestris
CERPA289-08.Trichoferus campestris
GBMNC63125-20.Trichoferus campestris
GBMNB25613-20.Trichoferus campestris
GBMNC63062-20.Trichoferus campestris
GBMNB25558-20.Trichoferus campestris
GBMNC63108-20.Trichoferus campestris
GBMNB25629-20.Trichoferus campestris
GBMNC63060-20.Trichoferus campestris
GBCLC967-19.Trichoferus campestris
CERPA407-08.Trichoferus campestris
CBGPC2316-21.Trichoferus campestris
GBMNC63080-20.Trichoferus campestris
GBMNC63072-20.Trichoferus campestris
GBMNC63053-20.Trichoferus campestris
GBMNB25633-20.Trichoferus campestris
GBMNC63130-20.Trichoferus campestris
GBMNC63135-20.Trichoferus campestris
GBMNC63076-20.Trichoferus campestris
GBMNC63077-20.Trichoferus campestris
GBMNC63140-20.Trichoferus campestris
CBGPC2315-21.Trichoferus campestris
GBMNC63070-20.Trichoferus campestris
GBCL10076-12.Trichoferus campestris
GBCL10075-12.Trichoferus campestris
VVGC039-08.Trichoferus campestris
GBMNC63126-20.Trichoferus campestris
GBMNB25619-20.Trichoferus campestris
GBMNC63123-20.Trichoferus campestris
GBMNB25622-20.Trichoferus campestris
GBMNC63061-20.Trichoferus campestris
GBMNB25557-20.Trichoferus campestris
PHDLV122-22.Trichoferus fasciculatus
GBMNB27227-20.Trichoferus fasciculatus
PHDLV225-22.Trichoferus fasciculatus
GCOL3736-16.Trichoferus fasciculatus
CBGPC167-21.Trichoferus fasciculatus
CBGPC168-21.Trichoferus fasciculatus
PSFOR017-13.Trichoferus pallidus
PSFOR097-13.Trichoferus pallidus
PHDLV163-22.Trichoferus pallidus
PHDLV240-22.Trichoferus pallidus
LPRCI2460-22.Trichoferus pallidus
CBGPC114-21.Trichoferus holosericeus
CBGPC115-21.Trichoferus holosericeus
CBGPC116-21.Trichoferus holosericeus
CBGPC117-21.Trichoferus holosericeus
GBMIN48526-17.Trichoferus holosericeus
CBGPC2296-21.Trichoferus holosericeus
PSFOR168-13.Trichoferus holosericeus
CBGPC164-21.Trichoferus holosericeus
CBGPC2308-21.Hesperophanes sericeus
PSFOR1112-17.Hesperophanes sericeus
GBCAB41039-24.Hesperophanes sericeus
CBGPC486-21.Chlorophorus figuratus
CBGPC1226-21.Chlorophorus figuratus
CBGPC488-21.Chlorophorus figuratus
CBGPC732-21.Chlorophorus figuratus
CBGPC1351-21.Chlorophorus figuratus
CBGPC1180-21.Chlorophorus figuratus
CBGPC1591-21.Chlorophorus figuratus
CBGPC1223-21.Chlorophorus figuratus
CBGPC1224-21.Chlorophorus figuratus
CBGPC1725-21.Chlorophorus figuratus
CBGPC1601-21.Chlorophorus figuratus
CBGPC1411-21.Chlorophorus figuratus
CBGPC1521-21.Chlorophorus figuratus
CBGPC1409-21.Chlorophorus figuratus
CBGPC1410-21.Chlorophorus figuratus
CBGPC1519-21.Chlorophorus figuratus
CBGPC1407-21.Chlorophorus figuratus
CBGPC1408-21.Chlorophorus figuratus
CBGPC1715-21.Chlorophorus figuratus
CBGPC1946-21.Chlorophorus figuratus
FBCOC375-10.Chlorophorus figuratus
FBCOK037-13.Chlorophorus figuratus
FBCOA292-10.Chlorophorus figuratus
CBGPC1788-21.Chlorophorus figuratus
PHDLV063-21.Chlorophorus glabromaculatus
CBGPC1094-21.Chlorophorus glabromaculatus
PHDLV251-22.Chlorophorus glabromaculatus
PSFOR112-13.Chlorophorus glabromaculatus
CBGPC1917-21.Chlorophorus glabromaculatus
GBMNE15604-21.Chlorophorus glabromaculatus
PHDLV064-21.Chlorophorus glabromaculatus
PHDLV302-22.Chlorophorus glabromaculatus
PHDLV139-22.Chlorophorus glabromaculatus
CBGPC417-21.Chlorophorus glabromaculatus
PHDLV233-22.Chlorophorus glaucus
COLFA534-12.Chlorophorus herbstii
GBCLC871-19.Chlorophorus herbstii
GBCLC872-19.Chlorophorus herbstii
CBGPC432-21.Chlorophorus ruficornis
CBGPC1441-21.Chlorophorus ruficornis
CBGPC439-21.Chlorophorus ruficornis
CBGPC1967-21.Chlorophorus sartor
PSFOR115-13.Chlorophorus sartor
CBGPC2003-21.Chlorophorus sartor
CBGPC1058-21.Chlorophorus sartor
CBGPC1593-21.Chlorophorus sartor
FBCOO053-13.Chlorophorus sartor

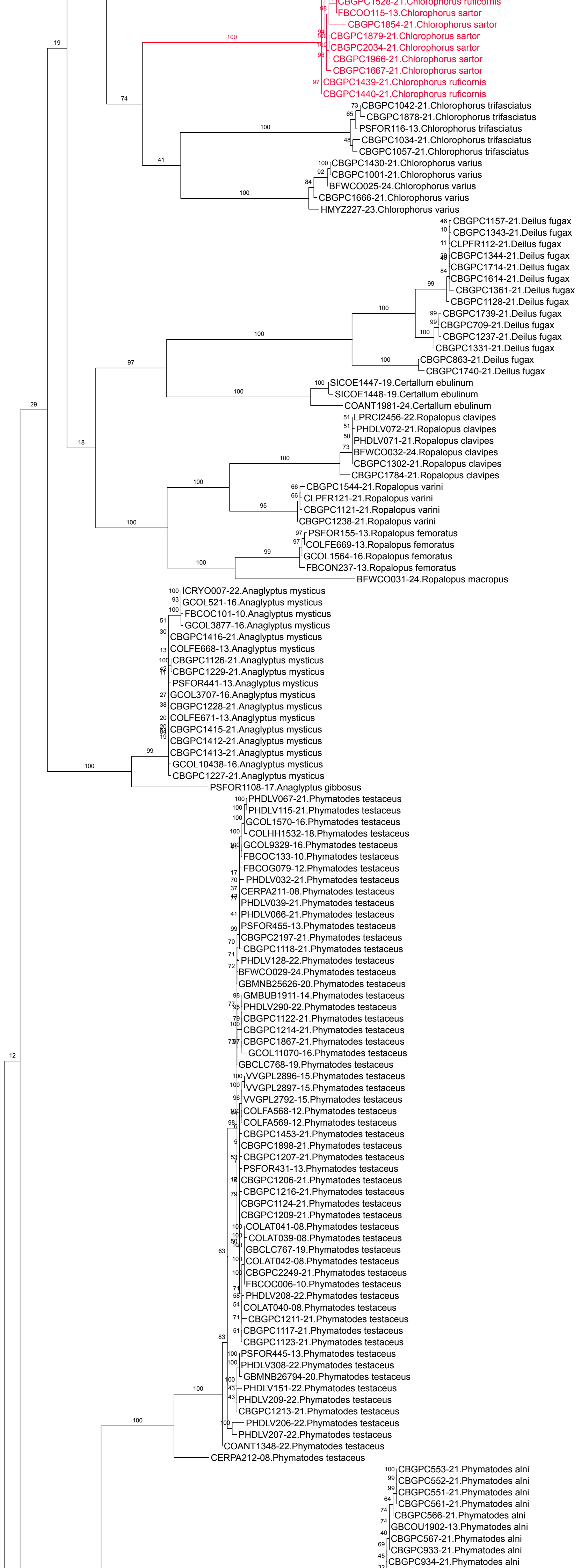
CBGPC1528-21.Chlorophorus ruficornis
FBCOO115-13.Chlorophorus sartor
CBGPC1854-21.Chlorophorus sartor
CBGPC1879-21.Chlorophorus sartor
CBGPC2034-21.Chlorophorus sartor
CBGPC1966-21.Chlorophorus sartor
CBGPC1667-21.Chlorophorus sartor
CBGPC1439-21.Chlorophorus ruficornis
CBGPC1440-21.Chlorophorus ruficornis
CBGPC1042-21.Chlorophorus trifasciatus
CBGPC1878-21.Chlorophorus trifasciatus
PSFOR116-13.Chlorophorus trifasciatus
CBGPC1034-21.Chlorophorus trifasciatus
CBGPC1057-21.Chlorophorus trifasciatus
CBGPC1430-21.Chlorophorus varius
CBGPC1001-21.Chlorophorus varius
BFWCO025-24.Chlorophorus varius
CBGPC1666-21.Chlorophorus varius
HMYZ227-23.Chlorophorus varius
CBGPC1157-21.Deilus fugax
CBGPC1343-21.Deilus fugax
CLPFR112-21.Deilus fugax
CBGPC1344-21.Deilus fugax
CBGPC1714-21.Deilus fugax
CBGPC1614-21.Deilus fugax
CBGPC1361-21.Deilus fugax
CBGPC1128-21.Deilus fugax
CBGPC1739-21.Deilus fugax
CBGPC709-21.Deilus fugax
CBGPC1237-21.Deilus fugax
CBGPC1331-21.Deilus fugax
CBGPC863-21.Deilus fugax
CBGPC1740-21.Deilus fugax
SICOE1447-19.Certallum ebulinum
SICOE1448-19.Certallum ebulinum
COANT1981-24.Certallum ebulinum
LPRCI2456-22.Ropalopus clavipes
PHDLV072-21.Ropalopus clavipes
PHDLV071-21.Ropalopus clavipes
BFWCO032-24.Ropalopus clavipes
CBGPC1302-21.Ropalopus clavipes
CBGPC1784-21.Ropalopus clavipes
CBGPC1544-21.Ropalopus varini
CLPFR121-21.Ropalopus varini
CBGPC1121-21.Ropalopus varini
CBGPC1238-21.Ropalopus varini
PSFOR155-13.Ropalopus femoratus
COLFE669-13.Ropalopus femoratus
GCOL1564-16.Ropalopus femoratus
FBCON237-13.Ropalopus femoratus
BFWCO031-24.Ropalopus macropus
ICRYO007-22.Anaglyptus mysticus
GCOL521-16.Anaglyptus mysticus
FBCOC101-10.Anaglyptus mysticus
GCOL3877-16.Anaglyptus mysticus
CBGPC1416-21.Anaglyptus mysticus
COLFE668-13.Anaglyptus mysticus
CBGPC1126-21.Anaglyptus mysticus
CBGPC1229-21.Anaglyptus mysticus
PSFOR441-13.Anaglyptus mysticus
GCOL3707-16.Anaglyptus mysticus
CBGPC1228-21.Anaglyptus mysticus
COLFE671-13.Anaglyptus mysticus
CBGPC1415-21.Anaglyptus mysticus
CBGPC1412-21.Anaglyptus mysticus
CBGPC1413-21.Anaglyptus mysticus
GCOL10438-16.Anaglyptus mysticus
CBGPC1227-21.Anaglyptus mysticus
PSFOR1108-17.Anaglyptus gibbosus
PHDLV067-21.Phymatodes testaceus
PHDLV115-21.Phymatodes testaceus
GCOL1570-16.Phymatodes testaceus
COLHH1532-18.Phymatodes testaceus
GCOL9329-16.Phymatodes testaceus
FBCOC133-10.Phymatodes testaceus
FBCOG079-12.Phymatodes testaceus
PHDLV032-21.Phymatodes testaceus
CERPA211-08.Phymatodes testaceus
PHDLV039-21.Phymatodes testaceus
PHDLV066-21.Phymatodes testaceus
PSFOR455-13.Phymatodes testaceus
CBGPC2197-21.Phymatodes testaceus
CBGPC1118-21.Phymatodes testaceus
PHDLV128-22.Phymatodes testaceus
BFWCO029-24.Phymatodes testaceus
GBMNB25626-20.Phymatodes testaceus
GMBUB1911-14.Phymatodes testaceus
PHDLV290-22.Phymatodes testaceus
CBGPC1122-21.Phymatodes testaceus
CBGPC1214-21.Phymatodes testaceus
CBGPC1867-21.Phymatodes testaceus
GCOL11070-16.Phymatodes testaceus
GBCLC768-19.Phymatodes testaceus
VVGPL2896-15.Phymatodes testaceus
VVGPL2897-15.Phymatodes testaceus
VVGPL2792-15.Phymatodes testaceus
COLFA568-12.Phymatodes testaceus
COLFA569-12.Phymatodes testaceus
CBGPC1453-21.Phymatodes testaceus
CBGPC1898-21.Phymatodes testaceus
CBGPC1207-21.Phymatodes testaceus
PSFOR431-13.Phymatodes testaceus
CBGPC1206-21.Phymatodes testaceus
CBGPC1216-21.Phymatodes testaceus
CBGPC1124-21.Phymatodes testaceus
CBGPC1209-21.Phymatodes testaceus
COLAT041-08.Phymatodes testaceus
COLAT039-08.Phymatodes testaceus
GBCLC767-19.Phymatodes testaceus
COLAT042-08.Phymatodes testaceus
CBGPC2249-21.Phymatodes testaceus
FBCOC006-10.Phymatodes testaceus
PHDLV208-22.Phymatodes testaceus
COLAT040-08.Phymatodes testaceus
CBGPC1211-21.Phymatodes testaceus
CBGPC1117-21.Phymatodes testaceus
CBGPC1123-21.Phymatodes testaceus
PSFOR445-13.Phymatodes testaceus
PHDLV308-22.Phymatodes testaceus
GBMNB26794-20.Phymatodes testaceus
PHDLV151-22.Phymatodes testaceus
PHDLV209-22.Phymatodes testaceus
CBGPC1213-21.Phymatodes testaceus
PHDLV206-22.Phymatodes testaceus
PHDLV207-22.Phymatodes testaceus
COANT1348-22.Phymatodes testaceus
CERPA212-08.Phymatodes testaceus
CBGPC553-21.Phymatodes alni
CBGPC552-21.Phymatodes alni
CBGPC551-21.Phymatodes alni
CBGPC561-21.Phymatodes alni
CBGPC566-21.Phymatodes alni
GBCOU1902-13.Phymatodes alni
CBGPC567-21.Phymatodes alni
CBGPC933-21.Phymatodes alni
CBGPC934-21.Phymatodes alni

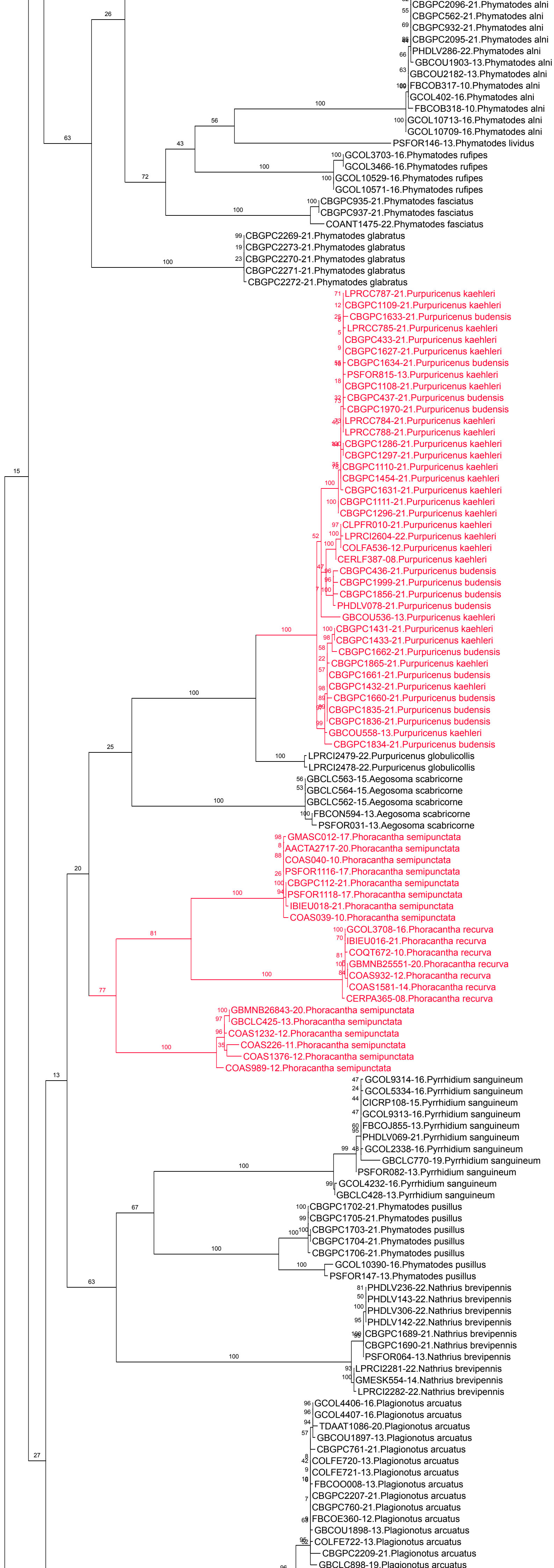
CBGPC2096-21.Phymatodes alni
CBGPC562-21.Phymatodes alni
CBGPC932-21.Phymatodes alni
CBGPC2095-21.Phymatodes alni
PHDLV286-22.Phymatodes alni
GBCOU1903-13.Phymatodes alni
GBCOU2182-13.Phymatodes alni
FBCOB317-10.Phymatodes alni
GCOL402-16.Phymatodes alni
FBCOB318-10.Phymatodes alni
GCOL10713-16.Phymatodes alni
GCOL10709-16.Phymatodes alni
PSFOR146-13.Phymatodes lividus
GCOL3703-16.Phymatodes rufipes
GCOL3466-16.Phymatodes rufipes
GCOL10529-16.Phymatodes rufipes
GCOL10571-16.Phymatodes rufipes
CBGPC935-21.Phymatodes fasciatus
CBGPC937-21.Phymatodes fasciatus
COANT1475-22.Phymatodes fasciatus
CBGPC2269-21.Phymatodes glabratus
CBGPC2273-21.Phymatodes glabratus
CBGPC2270-21.Phymatodes glabratus
CBGPC2271-21.Phymatodes glabratus
CBGPC2272-21.Phymatodes glabratus
LPRCC787-21.Purpuricenus kaehleri
CBGPC1109-21.Purpuricenus kaehleri
CBGPC1633-21.Purpuricenus budensis
LPRCC785-21.Purpuricenus kaehleri
CBGPC433-21.Purpuricenus kaehleri
CBGPC1627-21.Purpuricenus kaehleri
CBGPC1634-21.Purpuricenus budensis
PSFOR815-13.Purpuricenus kaehleri
CBGPC1108-21.Purpuricenus kaehleri
CBGPC437-21.Purpuricenus budensis
CBGPC1970-21.Purpuricenus budensis
LPRCC784-21.Purpuricenus kaehleri
LPRCC788-21.Purpuricenus kaehleri
CBGPC1286-21.Purpuricenus kaehleri
CBGPC1297-21.Purpuricenus kaehleri
CBGPC1110-21.Purpuricenus kaehleri
CBGPC1454-21.Purpuricenus kaehleri
CBGPC1631-21.Purpuricenus kaehleri
CBGPC1111-21.Purpuricenus kaehleri
CBGPC1296-21.Purpuricenus kaehleri
CLPFR010-21.Purpuricenus kaehleri
LPRCI2604-22.Purpuricenus kaehleri
COLFA536-12.Purpuricenus kaehleri
CERLF387-08.Purpuricenus kaehleri
CBGPC436-21.Purpuricenus budensis
CBGPC1999-21.Purpuricenus budensis
CBGPC1856-21.Purpuricenus budensis
PHDLV078-21.Purpuricenus budensis
GBCOU536-13.Purpuricenus kaehleri
CBGPC1431-21.Purpuricenus kaehleri
CBGPC1433-21.Purpuricenus kaehleri
CBGPC1662-21.Purpuricenus budensis
CBGPC1865-21.Purpuricenus kaehleri
CBGPC1661-21.Purpuricenus budensis
CBGPC1432-21.Purpuricenus kaehleri
CBGPC1660-21.Purpuricenus budensis
CBGPC1835-21.Purpuricenus budensis
CBGPC1836-21.Purpuricenus budensis
GBCOU558-13.Purpuricenus kaehleri
CBGPC1834-21.Purpuricenus budensis
LPRCI2479-22.Purpuricenus globulicollis
LPRCI2478-22.Purpuricenus globulicollis
GBCLC563-15.Aegosoma scabricorne
GBCLC564-15.Aegosoma scabricorne
GBCLC562-15.Aegosoma scabricorne
FBCON594-13.Aegosoma scabricorne
PSFOR031-13.Aegosoma scabricorne
GMASC012-17.Phoracantha semipunctata
AACTA2717-20.Phoracantha semipunctata
COAS040-10.Phoracantha semipunctata
PSFOR1116-17.Phoracantha semipunctata
CBGPC112-21.Phoracantha semipunctata
PSFOR1118-17.Phoracantha semipunctata
IBIEU018-21.Phoracantha semipunctata
COAS039-10.Phoracantha semipunctata
GCOL3708-16.Phoracantha recurva
IBIEU016-21.Phoracantha recurva
COQT672-10.Phoracantha recurva
GBMNB25551-20.Phoracantha recurva
COAS932-12.Phoracantha recurva
COAS1581-14.Phoracantha recurva
CERPA365-08.Phoracantha recurva
GBMNB26843-20.Phoracantha semipunctata
GBCLC425-13.Phoracantha semipunctata
COAS1232-12.Phoracantha semipunctata
COAS226-11.Phoracantha semipunctata
COAS1376-12.Phoracantha semipunctata
COAS989-12.Phoracantha semipunctata
GCOL9314-16.Pyrrhidium sanguineum
GCOL5334-16.Pyrrhidium sanguineum
CICRP108-15.Pyrrhidium sanguineum
GCOL9313-16.Pyrrhidium sanguineum
FBCOJ855-13.Pyrrhidium sanguineum
PHDLV069-21.Pyrrhidium sanguineum
GCOL2338-16.Pyrrhidium sanguineum
GBCLC770-19.Pyrrhidium sanguineum
PSFOR082-13.Pyrrhidium sanguineum
GCOL4232-16.Pyrrhidium sanguineum
GBCLC428-13.Pyrrhidium sanguineum
CBGPC1702-21.Phymatodes pusillus
CBGPC1705-21.Phymatodes pusillus
CBGPC1703-21.Phymatodes pusillus
CBGPC1704-21.Phymatodes pusillus
CBGPC1706-21.Phymatodes pusillus
GCOL10390-16.Phymatodes pusillus
PSFOR147-13.Phymatodes pusillus
PHDLV236-22.Nathrius brevipennis
PHDLV143-22.Nathrius brevipennis
PHDLV306-22.Nathrius brevipennis
PHDLV142-22.Nathrius brevipennis
CBGPC1689-21.Nathrius brevipennis
CBGPC1690-21.Nathrius brevipennis
PSFOR064-13.Nathrius brevipennis
LPRCI2281-22.Nathrius brevipennis
GMESK554-14.Nathrius brevipennis
LPRCI2282-22.Nathrius brevipennis
GCOL4406-16.Plagionotus arcuatus
GCOL4407-16.Plagionotus arcuatus
TDAAT1086-20.Plagionotus arcuatus
GBCOU1897-13.Plagionotus arcuatus
CBGPC761-21.Plagionotus arcuatus
COLFE720-13.Plagionotus arcuatus
COLFE721-13.Plagionotus arcuatus
FBCOO008-13.Plagionotus arcuatus
CBGPC2207-21.Plagionotus arcuatus
CBGPC760-21.Plagionotus arcuatus
FBCOE360-12.Plagionotus arcuatus
GBCOU1898-13.Plagionotus arcuatus
COLFE722-13.Plagionotus arcuatus
CBGPC2209-21.Plagionotus arcuatus
GBCLC898-19.Plagionotus arcuatus

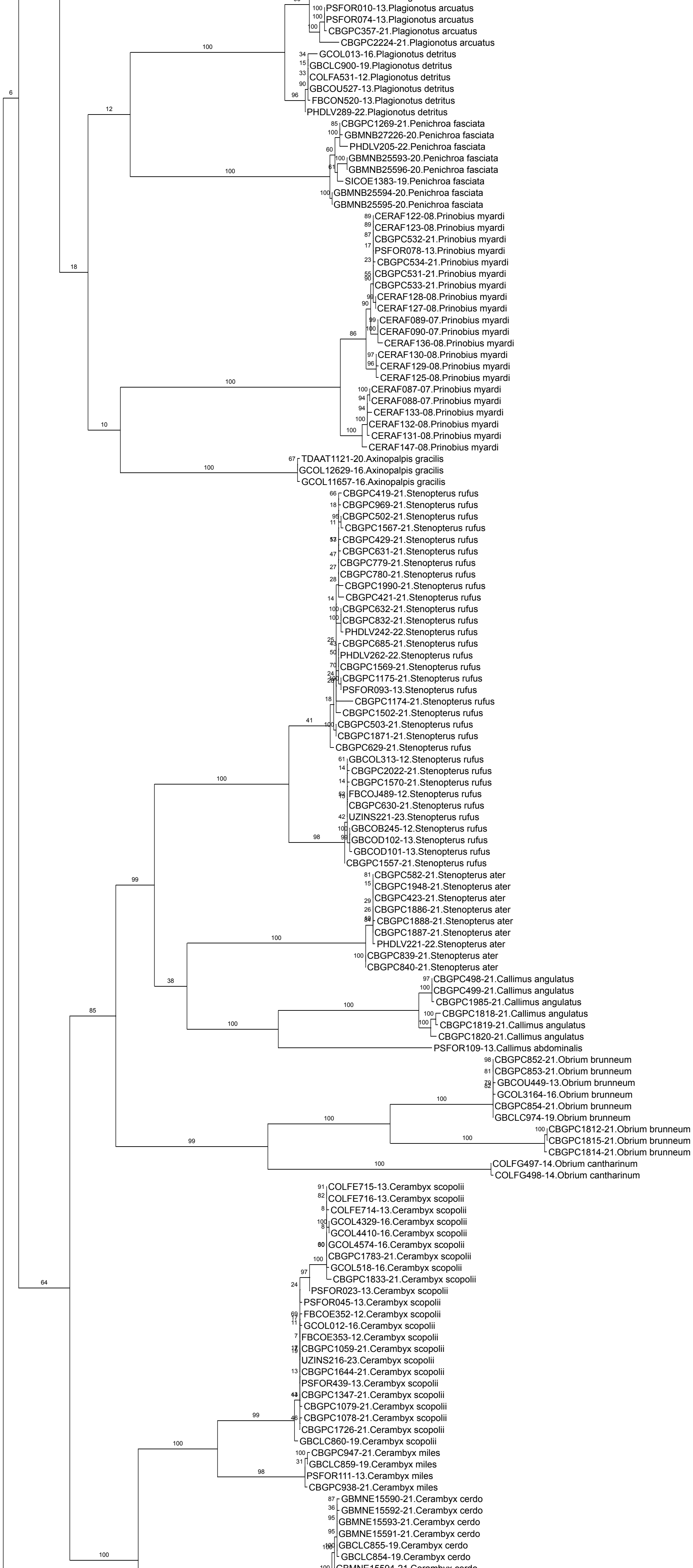
PSFOR010-13.Plagionotus arcuatus
PSFOR074-13.Plagionotus arcuatus
CBGPC357-21.Plagionotus arcuatus
CBGPC2224-21.Plagionotus arcuatus
GCOL013-16.Plagionotus detritus
GBCLC900-19.Plagionotus detritus
COLFA531-12.Plagionotus detritus
GBCOU527-13.Plagionotus detritus
FBCON520-13.Plagionotus detritus
PHDLV289-22.Plagionotus detritus
CBGPC1269-21.Penichroa fasciata
GBMNB27226-20.Penichroa fasciata
PHDLV205-22.Penichroa fasciata
GBMNB25593-20.Penichroa fasciata
GBMNB25596-20.Penichroa fasciata
SICOE1383-19.Penichroa fasciata
GBMNB25594-20.Penichroa fasciata
GBMNB25595-20.Penichroa fasciata
CERAF122-08.Prinobius myardi
CERAF123-08.Prinobius myardi
CBGPC532-21.Prinobius myardi
PSFOR078-13.Prinobius myardi
CBGPC534-21.Prinobius myardi
CBGPC531-21.Prinobius myardi
CBGPC533-21.Prinobius myardi
CERAF128-08.Prinobius myardi
CERAF127-08.Prinobius myardi
CERAF089-07.Prinobius myardi
CERAF090-07.Prinobius myardi
CERAF136-08.Prinobius myardi
CERAF130-08.Prinobius myardi
CERAF129-08.Prinobius myardi
CERAF125-08.Prinobius myardi
CERAF087-07.Prinobius myardi
CERAF088-07.Prinobius myardi
CERAF133-08.Prinobius myardi
CERAF132-08.Prinobius myardi
CERAF131-08.Prinobius myardi
CERAF147-08.Prinobius myardi
TDAAT1121-20.Axinopalpis gracilis
GCOL12629-16.Axinopalpis gracilis
GCOL11657-16.Axinopalpis gracilis
CBGPC419-21.Stenopterus rufus
CBGPC969-21.Stenopterus rufus
CBGPC502-21.Stenopterus rufus
CBGPC1567-21.Stenopterus rufus
CBGPC429-21.Stenopterus rufus
CBGPC631-21.Stenopterus rufus
CBGPC779-21.Stenopterus rufus
CBGPC780-21.Stenopterus rufus
CBGPC1990-21.Stenopterus rufus
CBGPC421-21.Stenopterus rufus
CBGPC632-21.Stenopterus rufus
CBGPC832-21.Stenopterus rufus
PHDLV242-22.Stenopterus rufus
CBGPC685-21.Stenopterus rufus
PHDLV262-22.Stenopterus rufus
CBGPC1569-21.Stenopterus rufus
CBGPC1175-21.Stenopterus rufus
PSFOR093-13.Stenopterus rufus
CBGPC1174-21.Stenopterus rufus
CBGPC1502-21.Stenopterus rufus
CBGPC503-21.Stenopterus rufus
CBGPC1871-21.Stenopterus rufus
CBGPC629-21.Stenopterus rufus
GBCOL313-12.Stenopterus rufus
CBGPC2022-21.Stenopterus rufus
CBGPC1570-21.Stenopterus rufus
FBCOJ489-12.Stenopterus rufus
CBGPC630-21.Stenopterus rufus
UZINS221-23.Stenopterus rufus
GBCOB245-12.Stenopterus rufus
GBCOD102-13.Stenopterus rufus
GBCOD101-13.Stenopterus rufus
CBGPC1557-21.Stenopterus rufus
CBGPC582-21.Stenopterus ater
CBGPC1948-21.Stenopterus ater
CBGPC423-21.Stenopterus ater
CBGPC1886-21.Stenopterus ater
CBGPC1888-21.Stenopterus ater
CBGPC1887-21.Stenopterus ater
PHDLV221-22.Stenopterus ater
CBGPC839-21.Stenopterus ater
CBGPC840-21.Stenopterus ater
CBGPC498-21.Callimus angulatus
CBGPC499-21.Callimus angulatus
CBGPC1985-21.Callimus angulatus
CBGPC1818-21.Callimus angulatus
CBGPC1819-21.Callimus angulatus
CBGPC1820-21.Callimus angulatus
PSFOR109-13.Callimus abdominalis
CBGPC852-21.Obrium brunneum
CBGPC853-21.Obrium brunneum
GBCOU449-13.Obrium brunneum
GCOL3164-16.Obrium brunneum
CBGPC854-21.Obrium brunneum
GBCLC974-19.Obrium brunneum
CBGPC1812-21.Obrium brunneum
CBGPC1815-21.Obrium brunneum
CBGPC1814-21.Obrium brunneum
COLFG497-14.Obrium cantharinum
COLFG498-14.Obrium cantharinum
COLFE715-13.Cerambyx scopolii
COLFE716-13.Cerambyx scopolii
COLFE714-13.Cerambyx scopolii
GCOL4329-16.Cerambyx scopolii
GCOL4410-16.Cerambyx scopolii
GCOL4574-16.Cerambyx scopolii
CBGPC1783-21.Cerambyx scopolii
GCOL518-16.Cerambyx scopolii
CBGPC1833-21.Cerambyx scopolii
PSFOR023-13.Cerambyx scopolii
PSFOR045-13.Cerambyx scopolii
FBCOE352-12.Cerambyx scopolii
GCOL012-16.Cerambyx scopolii
FBCOE353-12.Cerambyx scopolii
CBGPC1059-21.Cerambyx scopolii
UZINS216-23.Cerambyx scopolii
CBGPC1644-21.Cerambyx scopolii
PSFOR439-13.Cerambyx scopolii
CBGPC1347-21.Cerambyx scopolii
CBGPC1079-21.Cerambyx scopolii
CBGPC1078-21.Cerambyx scopolii
CBGPC1726-21.Cerambyx scopolii
GBCLC860-19.Cerambyx scopolii
CBGPC947-21.Cerambyx miles
GBCLC859-19.Cerambyx miles
PSFOR111-13.Cerambyx miles
CBGPC938-21.Cerambyx miles
GBMNE15590-21.Cerambyx cerdo
GBMNE15592-21.Cerambyx cerdo
GBMNE15593-21.Cerambyx cerdo
GBMNE15591-21.Cerambyx cerdo
GBCLC855-19.Cerambyx cerdo
GBCLC854-19.Cerambyx cerdo

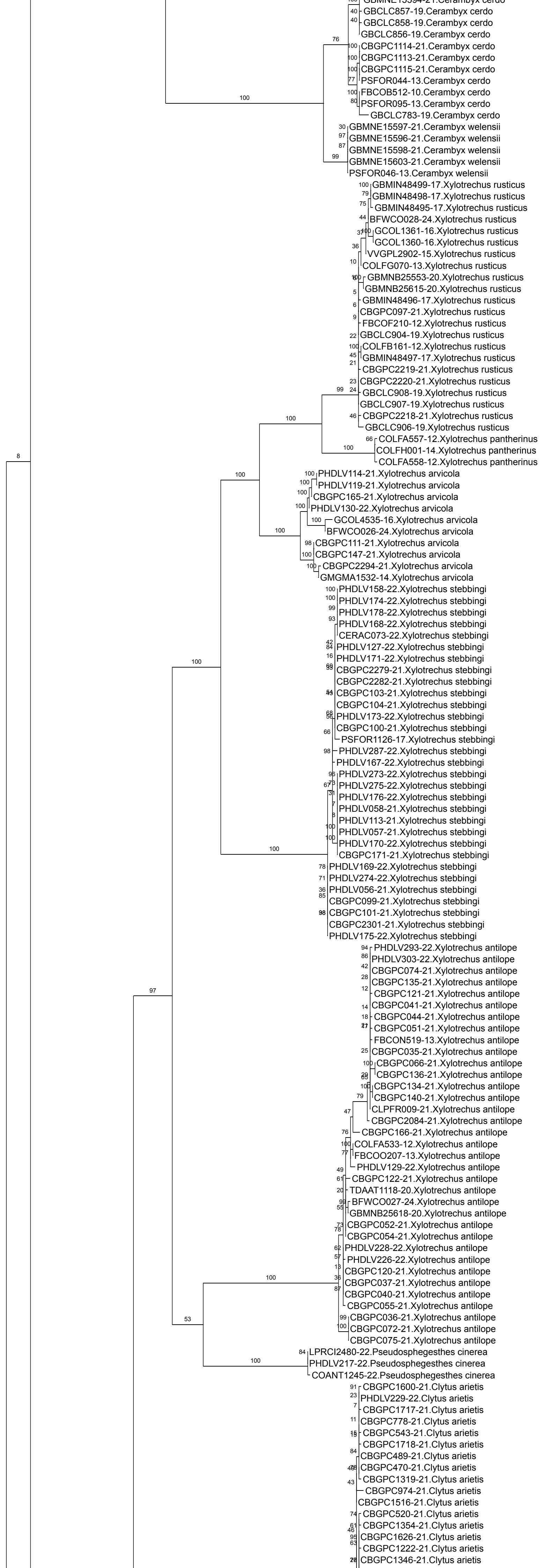
GBCLC857-19.Cerambyx cerdo
GBCLC858-19.Cerambyx cerdo
GBCLC856-19.Cerambyx cerdo
CBGPC1114-21.Cerambyx cerdo
CBGPC1113-21.Cerambyx cerdo
CBGPC1115-21.Cerambyx cerdo
PSFOR044-13.Cerambyx cerdo
FBCOB512-10.Cerambyx cerdo
PSFOR095-13.Cerambyx cerdo
GBCLC783-19.Cerambyx cerdo
GBMNE15597-21.Cerambyx welensii
GBMNE15596-21.Cerambyx welensii
GBMNE15598-21.Cerambyx welensii
GBMNE15603-21.Cerambyx welensii
PSFOR046-13.Cerambyx welensii
GBMIN48499-17.Xylotrechus rusticus
GBMIN48498-17.Xylotrechus rusticus
GBMIN48495-17.Xylotrechus rusticus
BFWCO028-24.Xylotrechus rusticus
GCOL1361-16.Xylotrechus rusticus
GCOL1360-16.Xylotrechus rusticus
VVGPL2902-15.Xylotrechus rusticus
COLFG070-13.Xylotrechus rusticus
GBMNB25553-20.Xylotrechus rusticus
GBMNB25615-20.Xylotrechus rusticus
GBMIN48496-17.Xylotrechus rusticus
CBGPC097-21.Xylotrechus rusticus
FBCOF210-12.Xylotrechus rusticus
GBCLC904-19.Xylotrechus rusticus
COLFB161-12.Xylotrechus rusticus
GBMIN48497-17.Xylotrechus rusticus
CBGPC2219-21.Xylotrechus rusticus
CBGPC2220-21.Xylotrechus rusticus
GBCLC908-19.Xylotrechus rusticus
GBCLC907-19.Xylotrechus rusticus
CBGPC2218-21.Xylotrechus rusticus
GBCLC906-19.Xylotrechus rusticus
COLFA557-12.Xylotrechus pantherinus
COLFH001-14.Xylotrechus pantherinus
COLFA558-12.Xylotrechus pantherinus
PHDLV114-21.Xylotrechus arvicola
PHDLV119-21.Xylotrechus arvicola
CBGPC165-21.Xylotrechus arvicola
PHDLV130-22.Xylotrechus arvicola
GCOL4535-16.Xylotrechus arvicola
BFWCO026-24.Xylotrechus arvicola
CBGPC111-21.Xylotrechus arvicola
CBGPC147-21.Xylotrechus arvicola
CBGPC2294-21.Xylotrechus arvicola
GMGMA1532-14.Xylotrechus arvicola
PHDLV158-22.Xylotrechus stebbingi
PHDLV174-22.Xylotrechus stebbingi
PHDLV178-22.Xylotrechus stebbingi
PHDLV168-22.Xylotrechus stebbingi
CERAC073-22.Xylotrechus stebbingi
PHDLV127-22.Xylotrechus stebbingi
PHDLV171-22.Xylotrechus stebbingi
CBGPC2279-21.Xylotrechus stebbingi
CBGPC2282-21.Xylotrechus stebbingi
CBGPC103-21.Xylotrechus stebbingi
CBGPC104-21.Xylotrechus stebbingi
PHDLV173-22.Xylotrechus stebbingi
CBGPC100-21.Xylotrechus stebbingi
PSFOR1126-17.Xylotrechus stebbingi
PHDLV287-22.Xylotrechus stebbingi
PHDLV167-22.Xylotrechus stebbingi
PHDLV273-22.Xylotrechus stebbingi
PHDLV275-22.Xylotrechus stebbingi
PHDLV176-22.Xylotrechus stebbingi
PHDLV058-21.Xylotrechus stebbingi
PHDLV113-21.Xylotrechus stebbingi
PHDLV057-21.Xylotrechus stebbingi
PHDLV170-22.Xylotrechus stebbingi
CBGPC171-21.Xylotrechus stebbingi
PHDLV169-22.Xylotrechus stebbingi
PHDLV274-22.Xylotrechus stebbingi
PHDLV056-21.Xylotrechus stebbingi
CBGPC099-21.Xylotrechus stebbingi
CBGPC101-21.Xylotrechus stebbingi
CBGPC2301-21.Xylotrechus stebbingi
PHDLV175-22.Xylotrechus stebbingi
PHDLV293-22.Xylotrechus antilope
PHDLV303-22.Xylotrechus antilope
CBGPC074-21.Xylotrechus antilope
CBGPC135-21.Xylotrechus antilope
CBGPC121-21.Xylotrechus antilope
CBGPC041-21.Xylotrechus antilope
CBGPC044-21.Xylotrechus antilope
CBGPC051-21.Xylotrechus antilope
FBCON519-13.Xylotrechus antilope
CBGPC035-21.Xylotrechus antilope
CBGPC066-21.Xylotrechus antilope
CBGPC136-21.Xylotrechus antilope
CBGPC134-21.Xylotrechus antilope
CBGPC140-21.Xylotrechus antilope
CLPFR009-21.Xylotrechus antilope
CBGPC2084-21.Xylotrechus antilope
CBGPC166-21.Xylotrechus antilope
COLFA533-12.Xylotrechus antilope
FBCOO207-13.Xylotrechus antilope
PHDLV129-22.Xylotrechus antilope
CBGPC122-21.Xylotrechus antilope
TDAAT1118-20.Xylotrechus antilope
BFWCO027-24.Xylotrechus antilope
GBMNB25618-20.Xylotrechus antilope
CBGPC052-21.Xylotrechus antilope
CBGPC054-21.Xylotrechus antilope
PHDLV228-22.Xylotrechus antilope
PHDLV226-22.Xylotrechus antilope
CBGPC120-21.Xylotrechus antilope
CBGPC037-21.Xylotrechus antilope
CBGPC040-21.Xylotrechus antilope
CBGPC055-21.Xylotrechus antilope
CBGPC036-21.Xylotrechus antilope
CBGPC072-21.Xylotrechus antilope
CBGPC075-21.Xylotrechus antilope
LPRCI2480-22.Pseudosphegesthes cinerea
PHDLV217-22.Pseudosphegesthes cinerea
COANT1245-22.Pseudosphegesthes cinerea
CBGPC1600-21.Clytus arietis
PHDLV229-22.Clytus arietis
CBGPC1717-21.Clytus arietis
CBGPC778-21.Clytus arietis
CBGPC543-21.Clytus arietis
CBGPC1718-21.Clytus arietis
CBGPC489-21.Clytus arietis
CBGPC470-21.Clytus arietis
CBGPC1319-21.Clytus arietis
CBGPC974-21.Clytus arietis
CBGPC1516-21.Clytus arietis
CBGPC520-21.Clytus arietis
CBGPC1354-21.Clytus arietis
CBGPC1626-21.Clytus arietis
CBGPC1222-21.Clytus arietis
CBGPC1346-21.Clytus arietis

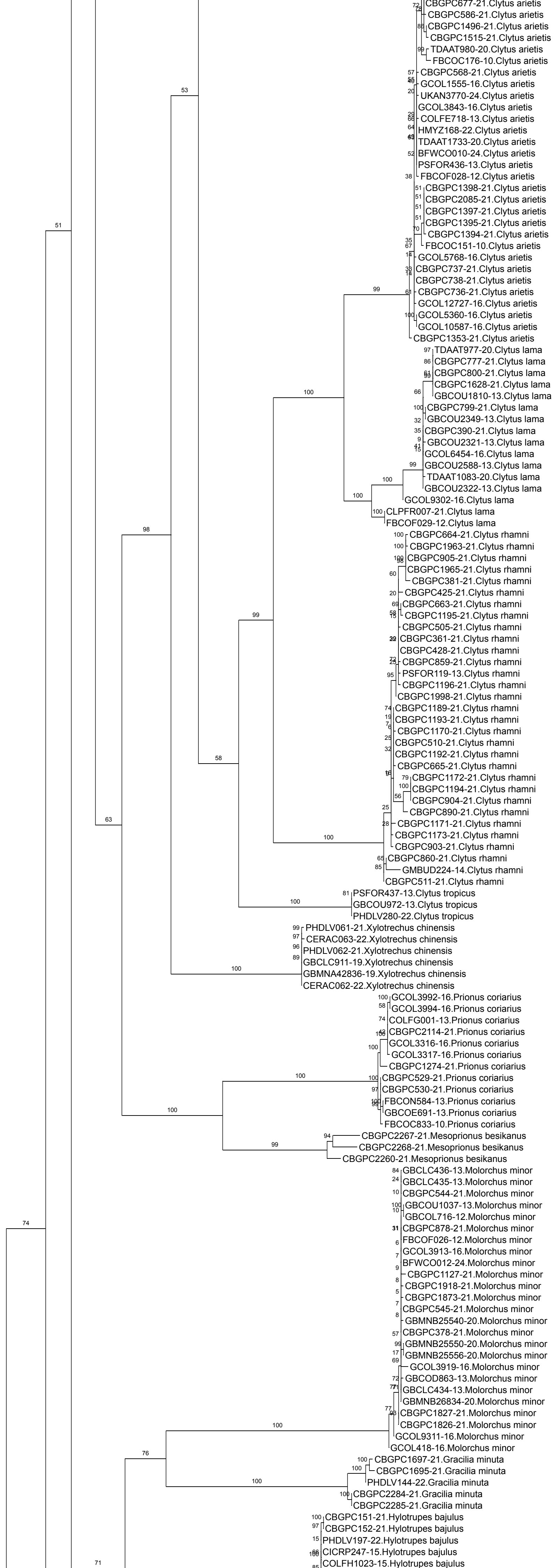
CBGPC677-21.Clytus arietis
CBGPC586-21.Clytus arietis
CBGPC1496-21.Clytus arietis
CBGPC1515-21.Clytus arietis
TDAAT980-20.Clytus arietis
FBCOC176-10.Clytus arietis
CBGPC568-21.Clytus arietis
GCOL1555-16.Clytus arietis
UKAN3770-24.Clytus arietis
GCOL3843-16.Clytus arietis
COLFE718-13.Clytus arietis
HMYZ168-22.Clytus arietis
TDAAT1733-20.Clytus arietis
BFWCO010-24.Clytus arietis
PSFOR436-13.Clytus arietis
FBCOF028-12.Clytus arietis
CBGPC1398-21.Clytus arietis
CBGPC2085-21.Clytus arietis
CBGPC1397-21.Clytus arietis
CBGPC1395-21.Clytus arietis
CBGPC1394-21.Clytus arietis
FBCOC151-10.Clytus arietis
GCOL5768-16.Clytus arietis
CBGPC737-21.Clytus arietis
CBGPC738-21.Clytus arietis
CBGPC736-21.Clytus arietis
GCOL12727-16.Clytus arietis
GCOL5360-16.Clytus arietis
GCOL10587-16.Clytus arietis
CBGPC1353-21.Clytus arietis
TDAAT977-20.Clytus lama
CBGPC777-21.Clytus lama
CBGPC800-21.Clytus lama
CBGPC1628-21.Clytus lama
GBCOU1810-13.Clytus lama
CBGPC799-21.Clytus lama
GBCOU2349-13.Clytus lama
CBGPC390-21.Clytus lama
GBCOU2321-13.Clytus lama
GCOL6454-16.Clytus lama
GBCOU2588-13.Clytus lama
TDAAT1083-20.Clytus lama
GBCOU2322-13.Clytus lama
GCOL9302-16.Clytus lama
CLPFR007-21.Clytus lama
FBCOF029-12.Clytus lama
CBGPC664-21.Clytus rhamni
CBGPC1963-21.Clytus rhamni
CBGPC905-21.Clytus rhamni
CBGPC1965-21.Clytus rhamni
CBGPC381-21.Clytus rhamni
CBGPC425-21.Clytus rhamni
CBGPC663-21.Clytus rhamni
CBGPC1195-21.Clytus rhamni
CBGPC505-21.Clytus rhamni
CBGPC361-21.Clytus rhamni
CBGPC428-21.Clytus rhamni
CBGPC859-21.Clytus rhamni
PSFOR119-13.Clytus rhamni
CBGPC1196-21.Clytus rhamni
CBGPC1998-21.Clytus rhamni
CBGPC1189-21.Clytus rhamni
CBGPC1193-21.Clytus rhamni
CBGPC1170-21.Clytus rhamni
CBGPC510-21.Clytus rhamni
CBGPC1192-21.Clytus rhamni
CBGPC665-21.Clytus rhamni
CBGPC1172-21.Clytus rhamni
CBGPC1194-21.Clytus rhamni
CBGPC904-21.Clytus rhamni
CBGPC890-21.Clytus rhamni
CBGPC1171-21.Clytus rhamni
CBGPC1173-21.Clytus rhamni
CBGPC903-21.Clytus rhamni
CBGPC860-21.Clytus rhamni
GMBUD224-14.Clytus rhamni
CBGPC511-21.Clytus rhamni
PSFOR437-13.Clytus tropicus
GBCOU972-13.Clytus tropicus
PHDLV280-22.Clytus tropicus
PHDLV061-21.Xylotrechus chinensis
CERAC063-22.Xylotrechus chinensis
PHDLV062-21.Xylotrechus chinensis
GBCLC911-19.Xylotrechus chinensis
GBMNA42836-19.Xylotrechus chinensis
CERAC062-22.Xylotrechus chinensis
GCOL3992-16.Prionus coriarius
GCOL3994-16.Prionus coriarius
COLFG001-13.Prionus coriarius
CBGPC2114-21.Prionus coriarius
GCOL3316-16.Prionus coriarius
GCOL3317-16.Prionus coriarius
CBGPC1274-21.Prionus coriarius
CBGPC529-21.Prionus coriarius
CBGPC530-21.Prionus coriarius
FBCON584-13.Prionus coriarius
GBCOE691-13.Prionus coriarius
FBCOC833-10.Prionus coriarius
CBGPC2267-21.Mesoprionus besikanus
CBGPC2268-21.Mesoprionus besikanus
CBGPC2260-21.Mesoprionus besikanus
GBCLC436-13.Molorchus minor
GBCLC435-13.Molorchus minor
CBGPC544-21.Molorchus minor
GBCOU1037-13.Molorchus minor
GBCOL716-12.Molorchus minor
CBGPC878-21.Molorchus minor
FBCOF026-12.Molorchus minor
GCOL3913-16.Molorchus minor
BFWCO012-24.Molorchus minor
CBGPC1127-21.Molorchus minor
CBGPC1918-21.Molorchus minor
CBGPC1873-21.Molorchus minor
CBGPC545-21.Molorchus minor
GBMNB25540-20.Molorchus minor
CBGPC378-21.Molorchus minor
GBMNB25550-20.Molorchus minor
GBMNB25556-20.Molorchus minor
GCOL3919-16.Molorchus minor
GBCOD863-13.Molorchus minor
GBCLC434-13.Molorchus minor
GBMNB26834-20.Molorchus minor
CBGPC1827-21.Molorchus minor
CBGPC1826-21.Molorchus minor
GCOL9311-16.Molorchus minor
GCOL418-16.Molorchus minor
CBGPC1697-21.Gracilia minuta
CBGPC1695-21.Gracilia minuta
PHDLV144-22.Gracilia minuta
CBGPC2284-21.Gracilia minuta
CBGPC2285-21.Gracilia minuta
CBGPC151-21.Hylotrupes bajulus
CBGPC152-21.Hylotrupes bajulus
PHDLV197-22.Hylotrupes bajulus
CICRP247-15.Hylotrupes bajulus
COLFH1023-15.Hylotrupes bajulus

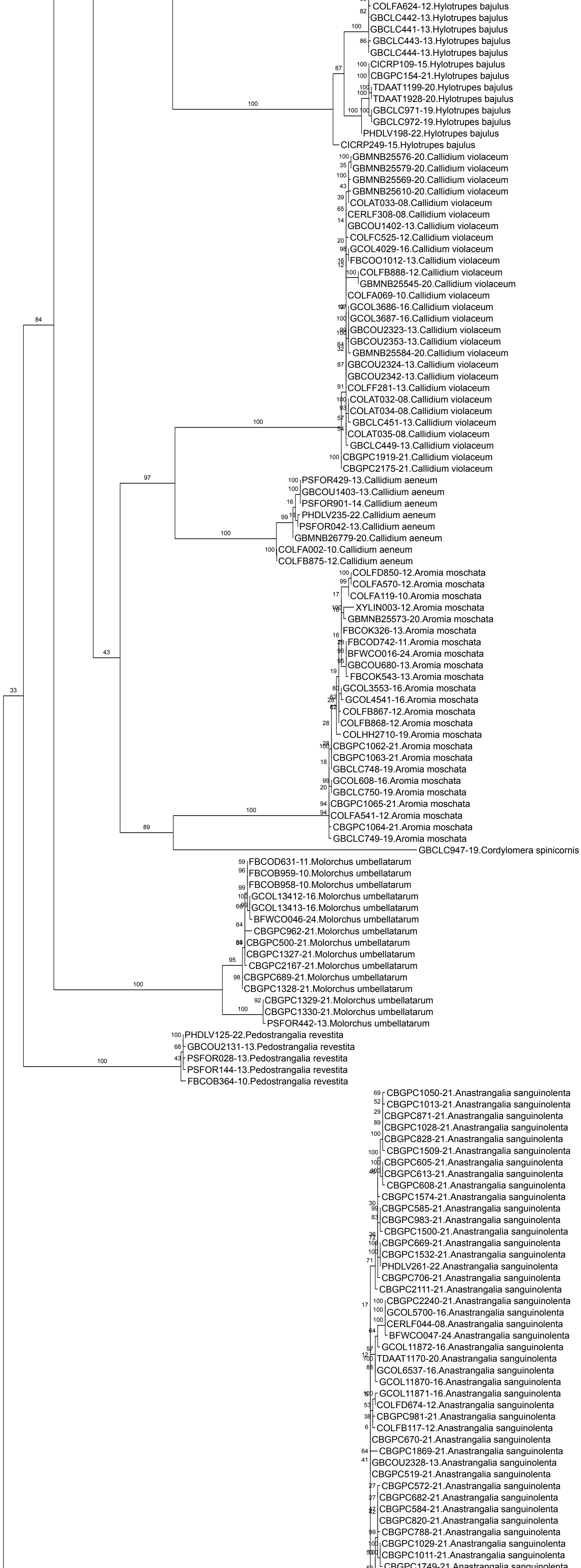

COLFA624-12.Hylotrupes bajulus
GBCLC442-13.Hylotrupes bajulus
GBCLC441-13.Hylotrupes bajulus
GBCLC443-13.Hylotrupes bajulus
GBCLC444-13.Hylotrupes bajulus
CICRP109-15.Hylotrupes bajulus
CBGPC154-21.Hylotrupes bajulus
TDAAT1199-20.Hylotrupes bajulus
TDAAT1928-20.Hylotrupes bajulus
GBCLC971-19.Hylotrupes bajulus
GBCLC972-19.Hylotrupes bajulus
PHDLV198-22.Hylotrupes bajulus
CICRP249-15.Hylotrupes bajulus
GBMNB25576-20.Callidium violaceum
GBMNB25579-20.Callidium violaceum
GBMNB25569-20.Callidium violaceum
GBMNB25610-20.Callidium violaceum
COLAT033-08.Callidium violaceum
CERLF308-08.Callidium violaceum
GBCOU1402-13.Callidium violaceum
COLFC525-12.Callidium violaceum
GCOL4029-16.Callidium violaceum
FBCOO1012-13.Callidium violaceum
COLFB888-12.Callidium violaceum
GBMNB25545-20.Callidium violaceum
COLFA069-10.Callidium violaceum
GCOL3686-16.Callidium violaceum
GCOL3687-16.Callidium violaceum
GBCOU2323-13.Callidium violaceum
GBCOU2353-13.Callidium violaceum
GBMNB25584-20.Callidium violaceum
GBCOU2324-13.Callidium violaceum
GBCOU2342-13.Callidium violaceum
COLFF281-13.Callidium violaceum
COLAT032-08.Callidium violaceum
COLAT034-08.Callidium violaceum
GBCLC451-13.Callidium violaceum
COLAT035-08.Callidium violaceum
GBCLC449-13.Callidium violaceum
CBGPC1919-21.Callidium violaceum
CBGPC2175-21.Callidium violaceum
PSFOR429-13.Callidium aeneum
GBCOU1403-13.Callidium aeneum
PSFOR901-14.Callidium aeneum
PHDLV235-22.Callidium aeneum
PSFOR042-13.Callidium aeneum
GBMNB26779-20.Callidium aeneum
COLFA002-10.Callidium aeneum
COLFB875-12.Callidium aeneum
COLFD850-12.Aromia moschata
COLFA570-12.Aromia moschata
COLFA119-10.Aromia moschata
XYLIN003-12.Aromia moschata
GBMNB25573-20.Aromia moschata
FBCOK326-13.Aromia moschata
FBCOD742-11.Aromia moschata
BFWCO016-24.Aromia moschata
GBCOU680-13.Aromia moschata
FBCOK543-13.Aromia moschata
GCOL3553-16.Aromia moschata
GCOL4541-16.Aromia moschata
COLFB867-12.Aromia moschata
COLFB868-12.Aromia moschata
COLHH2710-19.Aromia moschata
CBGPC1062-21.Aromia moschata
CBGPC1063-21.Aromia moschata
GBCLC748-19.Aromia moschata
GCOL608-16.Aromia moschata
GBCLC750-19.Aromia moschata
CBGPC1065-21.Aromia moschata
COLFA541-12.Aromia moschata
CBGPC1064-21.Aromia moschata
GBCLC749-19.Aromia moschata
GBCLC947-19.Cordylomera spinicornis
FBCOD631-11.Molorchus umbellatarum
FBCOB959-10.Molorchus umbellatarum
FBCOB958-10.Molorchus umbellatarum
GCOL13412-16.Molorchus umbellatarum
GCOL13413-16.Molorchus umbellatarum
BFWCO046-24.Molorchus umbellatarum
CBGPC962-21.Molorchus umbellatarum
CBGPC500-21.Molorchus umbellatarum
CBGPC1327-21.Molorchus umbellatarum
CBGPC2167-21.Molorchus umbellatarum
CBGPC689-21.Molorchus umbellatarum
CBGPC1328-21.Molorchus umbellatarum
CBGPC1329-21.Molorchus umbellatarum
CBGPC1330-21.Molorchus umbellatarum
PSFOR442-13.Molorchus umbellatarum
PHDLV125-22.Pedostrangalia revestita
GBCOU2131-13.Pedostrangalia revestita
PSFOR028-13.Pedostrangalia revestita
PSFOR144-13.Pedostrangalia revestita
FBCOB364-10.Pedostrangalia revestita
CBGPC1050-21.Anastrangalia sanguinolenta
CBGPC1013-21.Anastrangalia sanguinolenta
CBGPC871-21.Anastrangalia sanguinolenta
CBGPC1028-21.Anastrangalia sanguinolenta
CBGPC828-21.Anastrangalia sanguinolenta
CBGPC1509-21.Anastrangalia sanguinolenta
CBGPC605-21.Anastrangalia sanguinolenta
CBGPC613-21.Anastrangalia sanguinolenta
CBGPC608-21.Anastrangalia sanguinolenta
CBGPC1574-21.Anastrangalia sanguinolenta
CBGPC585-21.Anastrangalia sanguinolenta
CBGPC983-21.Anastrangalia sanguinolenta
CBGPC1500-21.Anastrangalia sanguinolenta
CBGPC669-21.Anastrangalia sanguinolenta
CBGPC1532-21.Anastrangalia sanguinolenta
PHDLV261-22.Anastrangalia sanguinolenta
CBGPC706-21.Anastrangalia sanguinolenta
CBGPC2111-21.Anastrangalia sanguinolenta
CBGPC2240-21.Anastrangalia sanguinolenta
GCOL5700-16.Anastrangalia sanguinolenta
CERLF044-08.Anastrangalia sanguinolenta
BFWCO047-24.Anastrangalia sanguinolenta
GCOL11872-16.Anastrangalia sanguinolenta
TDAAT1170-20.Anastrangalia sanguinolenta
GCOL6537-16.Anastrangalia sanguinolenta
GCOL11870-16.Anastrangalia sanguinolenta
GCOL11871-16.Anastrangalia sanguinolenta
COLFD674-12.Anastrangalia sanguinolenta
CBGPC981-21.Anastrangalia sanguinolenta
COLFB117-12.Anastrangalia sanguinolenta
CBGPC670-21.Anastrangalia sanguinolenta
CBGPC1869-21.Anastrangalia sanguinolenta
GBCOU2328-13.Anastrangalia sanguinolenta
CBGPC519-21.Anastrangalia sanguinolenta
CBGPC572-21.Anastrangalia sanguinolenta
CBGPC682-21.Anastrangalia sanguinolenta
CBGPC584-21.Anastrangalia sanguinolenta
CBGPC820-21.Anastrangalia sanguinolenta
CBGPC788-21.Anastrangalia sanguinolenta
CBGPC1029-21.Anastrangalia sanguinolenta
CBGPC1011-21.Anastrangalia sanguinolenta
84
33
43
97
89
100

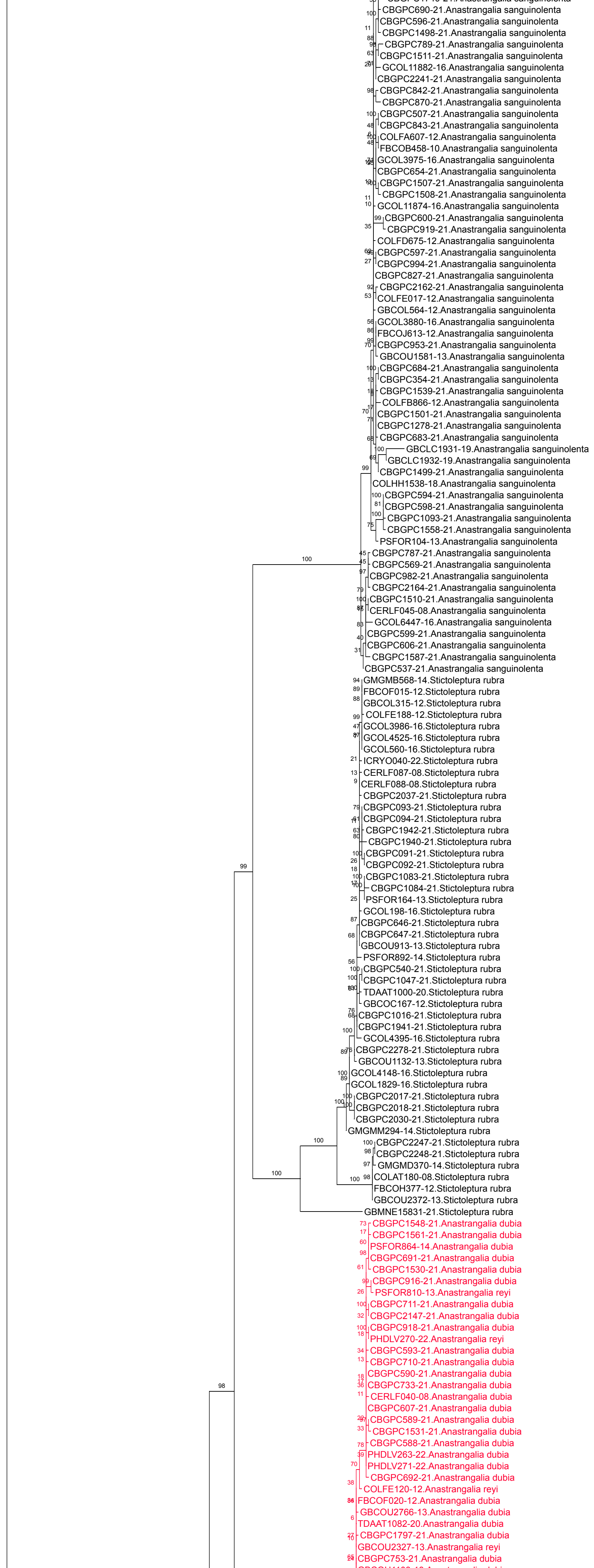
CBGPC690-21.Anastrangalia sanguinolenta
CBGPC596-21.Anastrangalia sanguinolenta
CBGPC1498-21.Anastrangalia sanguinolenta
CBGPC789-21.Anastrangalia sanguinolenta
CBGPC1511-21.Anastrangalia sanguinolenta
GCOL11882-16.Anastrangalia sanguinolenta
CBGPC2241-21.Anastrangalia sanguinolenta
CBGPC842-21.Anastrangalia sanguinolenta
CBGPC870-21.Anastrangalia sanguinolenta
CBGPC507-21.Anastrangalia sanguinolenta
CBGPC843-21.Anastrangalia sanguinolenta
COLFA607-12.Anastrangalia sanguinolenta
FBCOB458-10.Anastrangalia sanguinolenta
GCOL3975-16.Anastrangalia sanguinolenta
CBGPC654-21.Anastrangalia sanguinolenta
CBGPC1507-21.Anastrangalia sanguinolenta
CBGPC1508-21.Anastrangalia sanguinolenta
GCOL11874-16.Anastrangalia sanguinolenta
CBGPC600-21.Anastrangalia sanguinolenta
CBGPC919-21.Anastrangalia sanguinolenta
COLFD675-12.Anastrangalia sanguinolenta
CBGPC597-21.Anastrangalia sanguinolenta
CBGPC994-21.Anastrangalia sanguinolenta
CBGPC827-21.Anastrangalia sanguinolenta
CBGPC2162-21.Anastrangalia sanguinolenta
COLFE017-12.Anastrangalia sanguinolenta
GBCOL564-12.Anastrangalia sanguinolenta
GCOL3880-16.Anastrangalia sanguinolenta
FBCOJ613-12.Anastrangalia sanguinolenta
CBGPC953-21.Anastrangalia sanguinolenta
GBCOU1581-13.Anastrangalia sanguinolenta
CBGPC684-21.Anastrangalia sanguinolenta
CBGPC354-21.Anastrangalia sanguinolenta
CBGPC1539-21.Anastrangalia sanguinolenta
COLFB866-12.Anastrangalia sanguinolenta
CBGPC1501-21.Anastrangalia sanguinolenta
CBGPC1278-21.Anastrangalia sanguinolenta
CBGPC683-21.Anastrangalia sanguinolenta
GBCLC1931-19.Anastrangalia sanguinolenta
GBCLC1932-19.Anastrangalia sanguinolenta
CBGPC1499-21.Anastrangalia sanguinolenta
COLHH1538-18.Anastrangalia sanguinolenta
CBGPC594-21.Anastrangalia sanguinolenta
CBGPC598-21.Anastrangalia sanguinolenta
CBGPC1093-21.Anastrangalia sanguinolenta
CBGPC1558-21.Anastrangalia sanguinolenta
PSFOR104-13.Anastrangalia sanguinolenta
CBGPC787-21.Anastrangalia sanguinolenta
CBGPC569-21.Anastrangalia sanguinolenta
CBGPC982-21.Anastrangalia sanguinolenta
CBGPC2164-21.Anastrangalia sanguinolenta
CBGPC1510-21.Anastrangalia sanguinolenta
CERLF045-08.Anastrangalia sanguinolenta
GCOL6447-16.Anastrangalia sanguinolenta
CBGPC599-21.Anastrangalia sanguinolenta
CBGPC606-21.Anastrangalia sanguinolenta
CBGPC1587-21.Anastrangalia sanguinolenta
CBGPC537-21.Anastrangalia sanguinolenta
GMGMB568-14.Stictoleptura rubra
FBCOF015-12.Stictoleptura rubra
GBCOL315-12.Stictoleptura rubra
COLFE188-12.Stictoleptura rubra
GCOL3986-16.Stictoleptura rubra
GCOL4525-16.Stictoleptura rubra
GCOL560-16.Stictoleptura rubra
ICRYO040-22.Stictoleptura rubra
CERLF087-08.Stictoleptura rubra
CERLF088-08.Stictoleptura rubra
CBGPC2037-21.Stictoleptura rubra
CBGPC093-21.Stictoleptura rubra
CBGPC094-21.Stictoleptura rubra
CBGPC1942-21.Stictoleptura rubra
CBGPC1940-21.Stictoleptura rubra
CBGPC091-21.Stictoleptura rubra
CBGPC092-21.Stictoleptura rubra
CBGPC1083-21.Stictoleptura rubra
CBGPC1084-21.Stictoleptura rubra
PSFOR164-13.Stictoleptura rubra
GCOL198-16.Stictoleptura rubra
CBGPC646-21.Stictoleptura rubra
CBGPC647-21.Stictoleptura rubra
GBCOU913-13.Stictoleptura rubra
PSFOR892-14.Stictoleptura rubra
CBGPC540-21.Stictoleptura rubra
CBGPC1047-21.Stictoleptura rubra
TDAAT1000-20.Stictoleptura rubra
GBCOC167-12.Stictoleptura rubra
CBGPC1016-21.Stictoleptura rubra
CBGPC1941-21.Stictoleptura rubra
GCOL4395-16.Stictoleptura rubra
CBGPC2278-21.Stictoleptura rubra
GBCOU1132-13.Stictoleptura rubra
GCOL4148-16.Stictoleptura rubra
GCOL1829-16.Stictoleptura rubra
CBGPC2017-21.Stictoleptura rubra
CBGPC2018-21.Stictoleptura rubra
CBGPC2030-21.Stictoleptura rubra
GMGMM294-14.Stictoleptura rubra
CBGPC2247-21.Stictoleptura rubra
CBGPC2248-21.Stictoleptura rubra
GMGMD370-14.Stictoleptura rubra
COLAT180-08.Stictoleptura rubra
FBCOH377-12.Stictoleptura rubra
GBCOU2372-13.Stictoleptura rubra
GBMNE15831-21.Stictoleptura rubra
CBGPC1548-21.Anastrangalia dubia
CBGPC1561-21.Anastrangalia dubia
PSFOR864-14.Anastrangalia dubia
CBGPC691-21.Anastrangalia dubia
CBGPC1530-21.Anastrangalia dubia
CBGPC916-21.Anastrangalia dubia
PSFOR810-13.Anastrangalia reyi
CBGPC711-21.Anastrangalia dubia
CBGPC2147-21.Anastrangalia dubia
CBGPC918-21.Anastrangalia dubia
PHDLV270-22.Anastrangalia reyi
CBGPC593-21.Anastrangalia dubia
CBGPC710-21.Anastrangalia dubia
CBGPC590-21.Anastrangalia dubia
CBGPC733-21.Anastrangalia dubia
CERLF040-08.Anastrangalia dubia
CBGPC607-21.Anastrangalia dubia
CBGPC589-21.Anastrangalia dubia
CBGPC1531-21.Anastrangalia dubia
CBGPC588-21.Anastrangalia dubia
PHDLV263-22.Anastrangalia dubia
PHDLV271-22.Anastrangalia dubia
CBGPC692-21.Anastrangalia dubia
COLFE120-12.Anastrangalia reyi
FBCOF020-12.Anastrangalia dubia
GBCOU2766-13.Anastrangalia dubia
TDAAT1082-20.Anastrangalia dubia
CBGPC1797-21.Anastrangalia dubia
GBCOU2327-13.Anastrangalia reyi
CBGPC753-21.Anastrangalia dubia

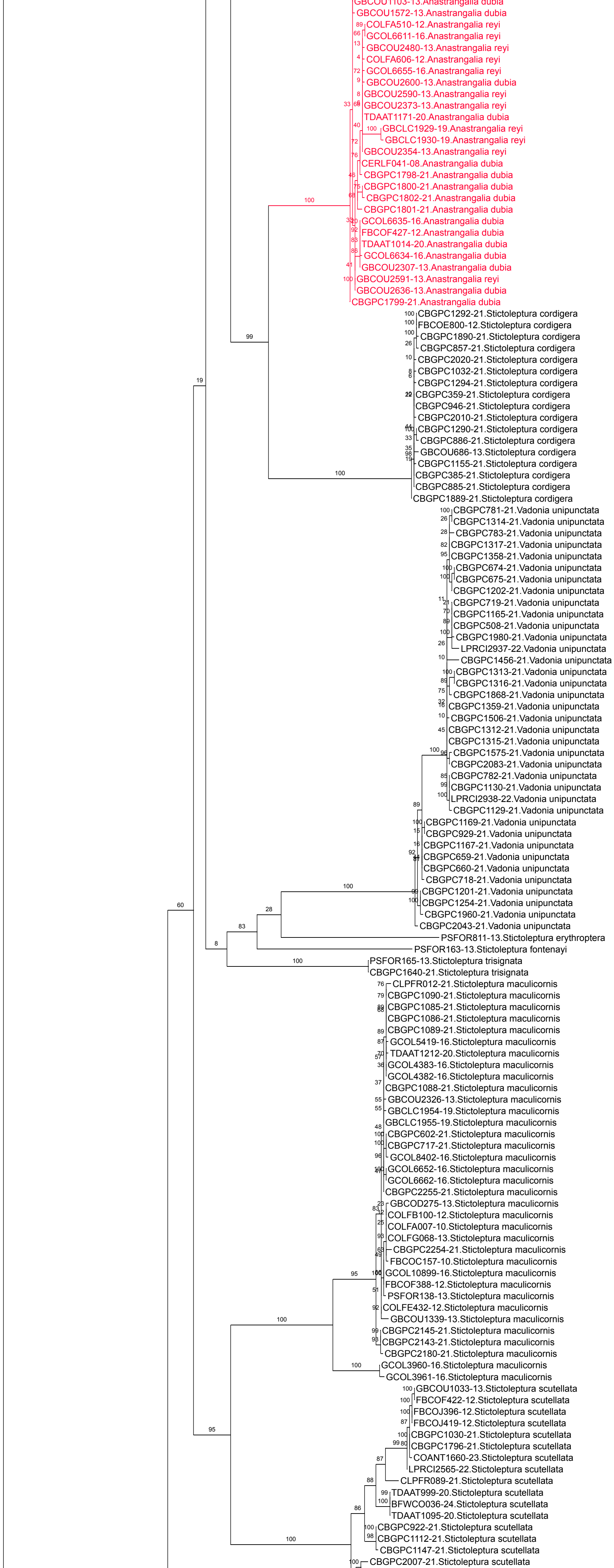

GBCOU1103-13.Anastrangalia dubia
GBCOU1572-13.Anastrangalia dubia
COLFA510-12.Anastrangalia reyi
GCOL6611-16.Anastrangalia reyi
GBCOU2480-13.Anastrangalia reyi
COLFA606-12.Anastrangalia reyi
GCOL6655-16.Anastrangalia reyi
GBCOU2600-13.Anastrangalia dubia
GBCOU2590-13.Anastrangalia reyi
GBCOU2373-13.Anastrangalia reyi
TDAAT1171-20.Anastrangalia dubia
GBCLC1929-19.Anastrangalia reyi
GBCLC1930-19.Anastrangalia reyi
GBCOU2354-13.Anastrangalia reyi
CERLF041-08.Anastrangalia dubia
CBGPC1798-21.Anastrangalia dubia
CBGPC1800-21.Anastrangalia dubia
CBGPC1802-21.Anastrangalia dubia
CBGPC1801-21.Anastrangalia dubia
GCOL6635-16.Anastrangalia dubia
FBCOF427-12.Anastrangalia dubia
TDAAT1014-20.Anastrangalia dubia
GCOL6634-16.Anastrangalia dubia
GBCOU2307-13.Anastrangalia dubia
GBCOU2591-13.Anastrangalia reyi
GBCOU2636-13.Anastrangalia dubia
CBGPC1799-21.Anastrangalia dubia
CBGPC1292-21.Stictoleptura cordigera
FBCOE800-12.Stictoleptura cordigera
CBGPC1890-21.Stictoleptura cordigera
CBGPC857-21.Stictoleptura cordigera
CBGPC2020-21.Stictoleptura cordigera
CBGPC1032-21.Stictoleptura cordigera
CBGPC1294-21.Stictoleptura cordigera
CBGPC359-21.Stictoleptura cordigera
CBGPC946-21.Stictoleptura cordigera
CBGPC2010-21.Stictoleptura cordigera
CBGPC1290-21.Stictoleptura cordigera
CBGPC886-21.Stictoleptura cordigera
GBCOU686-13.Stictoleptura cordigera
CBGPC1155-21.Stictoleptura cordigera
CBGPC385-21.Stictoleptura cordigera
CBGPC885-21.Stictoleptura cordigera
CBGPC1889-21.Stictoleptura cordigera
CBGPC781-21.Vadonia unipunctata
CBGPC1314-21.Vadonia unipunctata
CBGPC783-21.Vadonia unipunctata
CBGPC1317-21.Vadonia unipunctata
CBGPC1358-21.Vadonia unipunctata
CBGPC674-21.Vadonia unipunctata
CBGPC675-21.Vadonia unipunctata
CBGPC1202-21.Vadonia unipunctata
CBGPC719-21.Vadonia unipunctata
CBGPC1165-21.Vadonia unipunctata
CBGPC508-21.Vadonia unipunctata
CBGPC1980-21.Vadonia unipunctata
LPRCI2937-22.Vadonia unipunctata
CBGPC1456-21.Vadonia unipunctata
CBGPC1313-21.Vadonia unipunctata
CBGPC1316-21.Vadonia unipunctata
CBGPC1868-21.Vadonia unipunctata
CBGPC1359-21.Vadonia unipunctata
CBGPC1506-21.Vadonia unipunctata
CBGPC1312-21.Vadonia unipunctata
CBGPC1315-21.Vadonia unipunctata
CBGPC1575-21.Vadonia unipunctata
CBGPC2083-21.Vadonia unipunctata
CBGPC782-21.Vadonia unipunctata
CBGPC1130-21.Vadonia unipunctata
LPRCI2938-22.Vadonia unipunctata
CBGPC1129-21.Vadonia unipunctata
CBGPC1169-21.Vadonia unipunctata
CBGPC929-21.Vadonia unipunctata
CBGPC1167-21.Vadonia unipunctata
CBGPC659-21.Vadonia unipunctata
CBGPC660-21.Vadonia unipunctata
CBGPC718-21.Vadonia unipunctata
CBGPC1201-21.Vadonia unipunctata
CBGPC1254-21.Vadonia unipunctata
CBGPC1960-21.Vadonia unipunctata
CBGPC2043-21.Vadonia unipunctata
PSFOR811-13.Stictoleptura erythroptera
PSFOR163-13.Stictoleptura fontenayi
PSFOR165-13.Stictoleptura trisignata
CBGPC1640-21.Stictoleptura trisignata
CLPFR012-21.Stictoleptura maculicornis
CBGPC1090-21.Stictoleptura maculicornis
CBGPC1085-21.Stictoleptura maculicornis
CBGPC1086-21.Stictoleptura maculicornis
CBGPC1089-21.Stictoleptura maculicornis
GCOL5419-16.Stictoleptura maculicornis
TDAAT1212-20.Stictoleptura maculicornis
GCOL4383-16.Stictoleptura maculicornis
GCOL4382-16.Stictoleptura maculicornis
CBGPC1088-21.Stictoleptura maculicornis
GBCOU2326-13.Stictoleptura maculicornis
GBCLC1954-19.Stictoleptura maculicornis
GBCLC1955-19.Stictoleptura maculicornis
CBGPC602-21.Stictoleptura maculicornis
CBGPC717-21.Stictoleptura maculicornis
GCOL8402-16.Stictoleptura maculicornis
GCOL6652-16.Stictoleptura maculicornis
GCOL6662-16.Stictoleptura maculicornis
CBGPC2255-21.Stictoleptura maculicornis
GBCOD275-13.Stictoleptura maculicornis
COLFB100-12.Stictoleptura maculicornis
COLFA007-10.Stictoleptura maculicornis
COLFG068-13.Stictoleptura maculicornis
CBGPC2254-21.Stictoleptura maculicornis
FBCOC157-10.Stictoleptura maculicornis
GCOL10899-16.Stictoleptura maculicornis
FBCOF388-12.Stictoleptura maculicornis
PSFOR138-13.Stictoleptura maculicornis
COLFE432-12.Stictoleptura maculicornis
GBCOU1339-13.Stictoleptura maculicornis
CBGPC2145-21.Stictoleptura maculicornis
CBGPC2143-21.Stictoleptura maculicornis
CBGPC2180-21.Stictoleptura maculicornis
GCOL3960-16.Stictoleptura maculicornis
GCOL3961-16.Stictoleptura maculicornis
GBCOU1033-13.Stictoleptura scutellata
FBCOF422-12.Stictoleptura scutellata
FBCOJ396-12.Stictoleptura scutellata
FBCOJ419-12.Stictoleptura scutellata
CBGPC1030-21.Stictoleptura scutellata
CBGPC1796-21.Stictoleptura scutellata
COANT1660-23.Stictoleptura scutellata
LPRCI2565-22.Stictoleptura scutellata
CLPFR089-21.Stictoleptura scutellata
TDAAT999-20.Stictoleptura scutellata
BFWCO036-24.Stictoleptura scutellata
TDAAT1095-20.Stictoleptura scutellata
CBGPC922-21.Stictoleptura scutellata
CBGPC1112-21.Stictoleptura scutellata
CBGPC1147-21.Stictoleptura scutellata
CBGPC2007-21.Stictoleptura scutellata

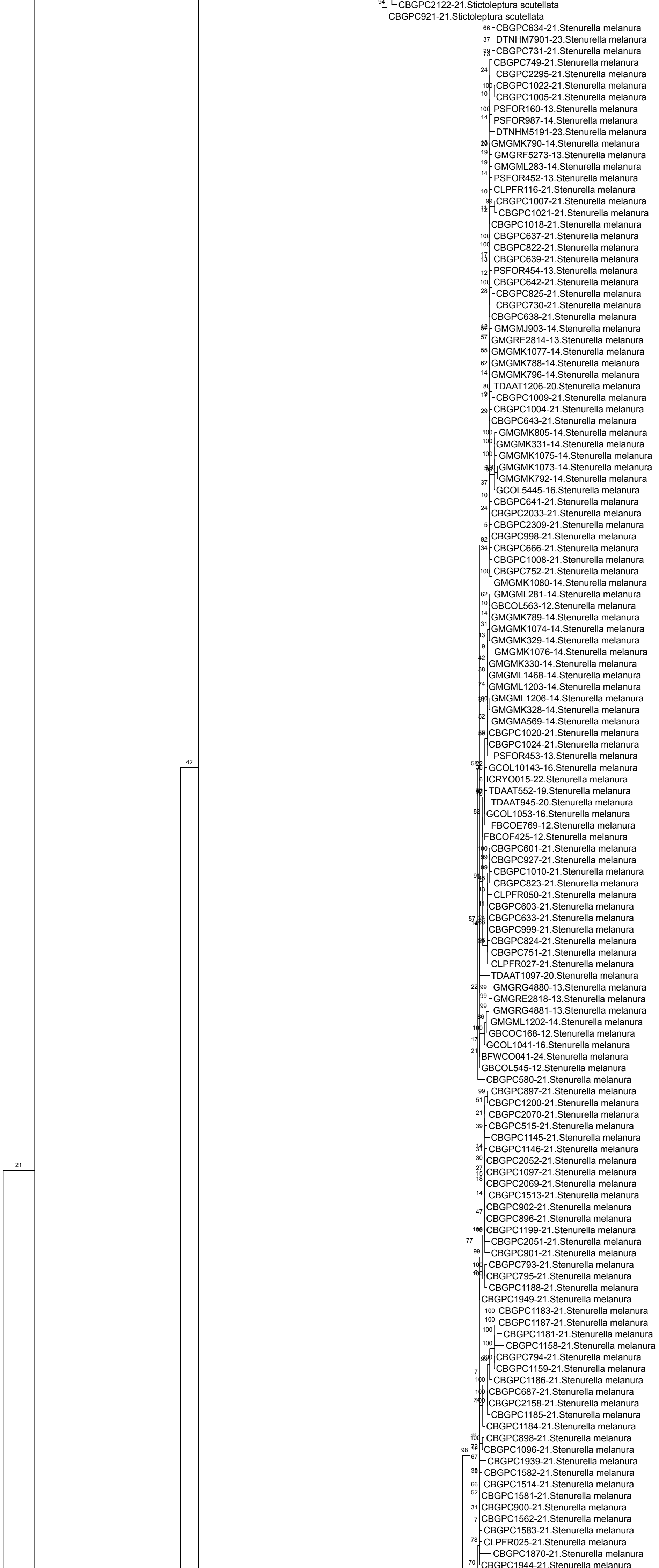
CBGPC2122-21.Stictoleptura scutellata
CBGPC921-21.Stictoleptura scutellata
CBGPC634-21.Stenurella melanura
DTNHM7901-23.Stenurella melanura
CBGPC731-21.Stenurella melanura
CBGPC749-21.Stenurella melanura
CBGPC2295-21.Stenurella melanura
CBGPC1022-21.Stenurella melanura
CBGPC1005-21.Stenurella melanura
PSFOR160-13.Stenurella melanura
PSFOR987-14.Stenurella melanura
DTNHM5191-23.Stenurella melanura
GMGMK790-14.Stenurella melanura
GMGRF5273-13.Stenurella melanura
GMGML283-14.Stenurella melanura
PSFOR452-13.Stenurella melanura
CLPFR116-21.Stenurella melanura
CBGPC1007-21.Stenurella melanura
CBGPC1021-21.Stenurella melanura
CBGPC1018-21.Stenurella melanura
CBGPC637-21.Stenurella melanura
CBGPC822-21.Stenurella melanura
CBGPC639-21.Stenurella melanura
PSFOR454-13.Stenurella melanura
CBGPC642-21.Stenurella melanura
CBGPC825-21.Stenurella melanura
CBGPC730-21.Stenurella melanura
CBGPC638-21.Stenurella melanura
GMGMJ903-14.Stenurella melanura
GMGRE2814-13.Stenurella melanura
GMGMK1077-14.Stenurella melanura
GMGMK788-14.Stenurella melanura
GMGMK796-14.Stenurella melanura
TDAAT1206-20.Stenurella melanura
CBGPC1009-21.Stenurella melanura
CBGPC1004-21.Stenurella melanura
CBGPC643-21.Stenurella melanura
GMGMK805-14.Stenurella melanura
GMGMK331-14.Stenurella melanura
GMGMK1075-14.Stenurella melanura
GMGMK1073-14.Stenurella melanura
GMGMK792-14.Stenurella melanura
GCOL5445-16.Stenurella melanura
CBGPC641-21.Stenurella melanura
CBGPC2033-21.Stenurella melanura
CBGPC2309-21.Stenurella melanura
CBGPC998-21.Stenurella melanura
CBGPC666-21.Stenurella melanura
CBGPC1008-21.Stenurella melanura
CBGPC752-21.Stenurella melanura
GMGMK1080-14.Stenurella melanura
GMGML281-14.Stenurella melanura
GBCOL563-12.Stenurella melanura
GMGMK789-14.Stenurella melanura
GMGMK1074-14.Stenurella melanura
GMGMK329-14.Stenurella melanura
GMGMK1076-14.Stenurella melanura
GMGMK330-14.Stenurella melanura
GMGML1468-14.Stenurella melanura
GMGML1203-14.Stenurella melanura
GMGML1206-14.Stenurella melanura
GMGMK328-14.Stenurella melanura
GMGMA569-14.Stenurella melanura
CBGPC1020-21.Stenurella melanura
CBGPC1024-21.Stenurella melanura
PSFOR453-13.Stenurella melanura
GCOL10143-16.Stenurella melanura
ICRYO015-22.Stenurella melanura
TDAAT552-19.Stenurella melanura
TDAAT945-20.Stenurella melanura
GCOL1053-16.Stenurella melanura
FBCOE769-12.Stenurella melanura
FBCOF425-12.Stenurella melanura
CBGPC601-21.Stenurella melanura
CBGPC927-21.Stenurella melanura
CBGPC1010-21.Stenurella melanura
CBGPC823-21.Stenurella melanura
CLPFR050-21.Stenurella melanura
CBGPC603-21.Stenurella melanura
CBGPC633-21.Stenurella melanura
CBGPC999-21.Stenurella melanura
CBGPC824-21.Stenurella melanura
CBGPC751-21.Stenurella melanura
CLPFR027-21.Stenurella melanura
TDAAT1097-20.Stenurella melanura
GMGRG4880-13.Stenurella melanura
GMGRE2818-13.Stenurella melanura
GMGRG4881-13.Stenurella melanura
GMGML1202-14.Stenurella melanura
GBCOC168-12.Stenurella melanura
GCOL1041-16.Stenurella melanura
BFWCO041-24.Stenurella melanura
GBCOL545-12.Stenurella melanura
CBGPC580-21.Stenurella melanura
CBGPC897-21.Stenurella melanura
CBGPC1200-21.Stenurella melanura
CBGPC2070-21.Stenurella melanura
CBGPC515-21.Stenurella melanura
CBGPC1145-21.Stenurella melanura
CBGPC1146-21.Stenurella melanura
CBGPC2052-21.Stenurella melanura
CBGPC1097-21.Stenurella melanura
CBGPC2069-21.Stenurella melanura
CBGPC1513-21.Stenurella melanura
CBGPC902-21.Stenurella melanura
CBGPC896-21.Stenurella melanura
CBGPC1199-21.Stenurella melanura
CBGPC2051-21.Stenurella melanura
CBGPC901-21.Stenurella melanura
CBGPC793-21.Stenurella melanura
CBGPC795-21.Stenurella melanura
CBGPC1188-21.Stenurella melanura
CBGPC1949-21.Stenurella melanura
CBGPC1183-21.Stenurella melanura
CBGPC1187-21.Stenurella melanura
CBGPC1181-21.Stenurella melanura
CBGPC1158-21.Stenurella melanura
CBGPC794-21.Stenurella melanura
CBGPC1159-21.Stenurella melanura
CBGPC1186-21.Stenurella melanura
CBGPC687-21.Stenurella melanura
CBGPC2158-21.Stenurella melanura
CBGPC1185-21.Stenurella melanura
CBGPC1184-21.Stenurella melanura
CBGPC898-21.Stenurella melanura
CBGPC1096-21.Stenurella melanura
CBGPC1939-21.Stenurella melanura
CBGPC1582-21.Stenurella melanura
CBGPC1514-21.Stenurella melanura
CBGPC1581-21.Stenurella melanura
CBGPC900-21.Stenurella melanura
CBGPC1562-21.Stenurella melanura
CBGPC1583-21.Stenurella melanura
CLPFR025-21.Stenurella melanura
CBGPC1870-21.Stenurella melanura
CBGPC1944-21.Stenurella melanura

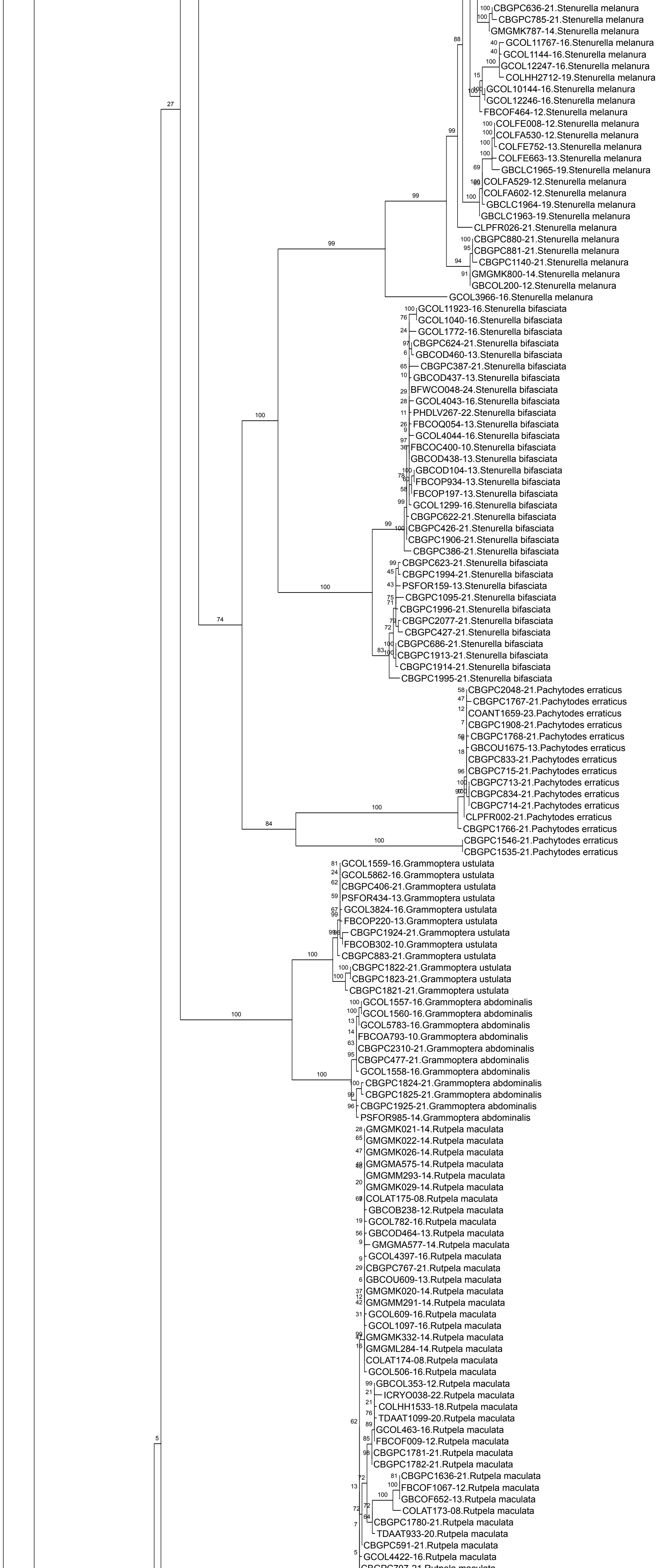
CBGPC636-21.Stenurella melanura
CBGPC785-21.Stenurella melanura
GMGMK787-14.Stenurella melanura
GCOL11767-16.Stenurella melanura
GCOL1144-16.Stenurella melanura
GCOL12247-16.Stenurella melanura
COLHH2712-19.Stenurella melanura
GCOL10144-16.Stenurella melanura
GCOL12246-16.Stenurella melanura
FBCOF464-12.Stenurella melanura
COLFE008-12.Stenurella melanura
COLFA530-12.Stenurella melanura
COLFE752-13.Stenurella melanura
COLFE663-13.Stenurella melanura
GBCLC1965-19.Stenurella melanura
COLFA529-12.Stenurella melanura
COLFA602-12.Stenurella melanura
GBCLC1964-19.Stenurella melanura
GBCLC1963-19.Stenurella melanura
CLPFR026-21.Stenurella melanura
CBGPC880-21.Stenurella melanura
CBGPC881-21.Stenurella melanura
CBGPC1140-21.Stenurella melanura
GMGMK800-14.Stenurella melanura
GBCOL200-12.Stenurella melanura
GCOL3966-16.Stenurella melanura
GCOL11923-16.Stenurella bifasciata
GCOL1040-16.Stenurella bifasciata
GCOL1772-16.Stenurella bifasciata
CBGPC624-21.Stenurella bifasciata
GBCOD460-13.Stenurella bifasciata
CBGPC387-21.Stenurella bifasciata
GBCOD437-13.Stenurella bifasciata
BFWCO048-24.Stenurella bifasciata
GCOL4043-16.Stenurella bifasciata
PHDLV267-22.Stenurella bifasciata
FBCOQ054-13.Stenurella bifasciata
GCOL4044-16.Stenurella bifasciata
FBCOC400-10.Stenurella bifasciata
GBCOD438-13.Stenurella bifasciata
GBCOD104-13.Stenurella bifasciata
FBCOP934-13.Stenurella bifasciata
FBCOP197-13.Stenurella bifasciata
GCOL1299-16.Stenurella bifasciata
CBGPC622-21.Stenurella bifasciata
CBGPC426-21.Stenurella bifasciata
CBGPC1906-21.Stenurella bifasciata
CBGPC386-21.Stenurella bifasciata
CBGPC623-21.Stenurella bifasciata
CBGPC1994-21.Stenurella bifasciata
PSFOR159-13.Stenurella bifasciata
CBGPC1095-21.Stenurella bifasciata
CBGPC1996-21.Stenurella bifasciata
CBGPC2077-21.Stenurella bifasciata
CBGPC427-21.Stenurella bifasciata
CBGPC686-21.Stenurella bifasciata
CBGPC1913-21.Stenurella bifasciata
CBGPC1914-21.Stenurella bifasciata
CBGPC1995-21.Stenurella bifasciata
CBGPC2048-21.Pachytodes erraticus
CBGPC1767-21.Pachytodes erraticus
COANT1659-23.Pachytodes erraticus
CBGPC1908-21.Pachytodes erraticus
CBGPC1768-21.Pachytodes erraticus
GBCOU1675-13.Pachytodes erraticus
CBGPC833-21.Pachytodes erraticus
CBGPC715-21.Pachytodes erraticus
CBGPC713-21.Pachytodes erraticus
CBGPC834-21.Pachytodes erraticus
CBGPC714-21.Pachytodes erraticus
CLPFR002-21.Pachytodes erraticus
CBGPC1766-21.Pachytodes erraticus
CBGPC1546-21.Pachytodes erraticus
CBGPC1535-21.Pachytodes erraticus
GCOL1559-16.Grammoptera ustulata
GCOL5862-16.Grammoptera ustulata
CBGPC406-21.Grammoptera ustulata
PSFOR434-13.Grammoptera ustulata
GCOL3824-16.Grammoptera ustulata
FBCOP220-13.Grammoptera ustulata
CBGPC1924-21.Grammoptera ustulata
FBCOB302-10.Grammoptera ustulata
CBGPC883-21.Grammoptera ustulata
CBGPC1822-21.Grammoptera ustulata
CBGPC1823-21.Grammoptera ustulata
CBGPC1821-21.Grammoptera ustulata
GCOL1557-16.Grammoptera abdominalis
GCOL1560-16.Grammoptera abdominalis
GCOL5783-16.Grammoptera abdominalis
FBCOA793-10.Grammoptera abdominalis
CBGPC2310-21.Grammoptera abdominalis
CBGPC477-21.Grammoptera abdominalis
GCOL1558-16.Grammoptera abdominalis
CBGPC1824-21.Grammoptera abdominalis
CBGPC1825-21.Grammoptera abdominalis
CBGPC1925-21.Grammoptera abdominalis
PSFOR985-14.Grammoptera abdominalis
GMGMK021-14.Rutpela maculata
GMGMK022-14.Rutpela maculata
GMGMK026-14.Rutpela maculata
GMGMA575-14.Rutpela maculata
GMGMM293-14.Rutpela maculata
GMGMK029-14.Rutpela maculata
COLAT175-08.Rutpela maculata
GBCOB238-12.Rutpela maculata
GCOL782-16.Rutpela maculata
GBCOD464-13.Rutpela maculata
GMGMA577-14.Rutpela maculata
GCOL4397-16.Rutpela maculata
CBGPC767-21.Rutpela maculata
GBCOU609-13.Rutpela maculata
GMGMK020-14.Rutpela maculata
GMGMM291-14.Rutpela maculata
GCOL609-16.Rutpela maculata
GCOL1097-16.Rutpela maculata
GMGMK332-14.Rutpela maculata
GMGML284-14.Rutpela maculata
COLAT174-08.Rutpela maculata
GCOL506-16.Rutpela maculata
GBCOL353-12.Rutpela maculata
ICRYO038-22.Rutpela maculata
COLHH1533-18.Rutpela maculata
TDAAT1099-20.Rutpela maculata
GCOL463-16.Rutpela maculata
FBCOF009-12.Rutpela maculata
CBGPC1781-21.Rutpela maculata
CBGPC1782-21.Rutpela maculata
CBGPC1636-21.Rutpela maculata
FBCOF1067-12.Rutpela maculata
GBCOF652-13.Rutpela maculata
COLAT173-08.Rutpela maculata
CBGPC1780-21.Rutpela maculata
TDAAT933-20.Rutpela maculata
CBGPC591-21.Rutpela maculata
GCOL4422-16.Rutpela maculata

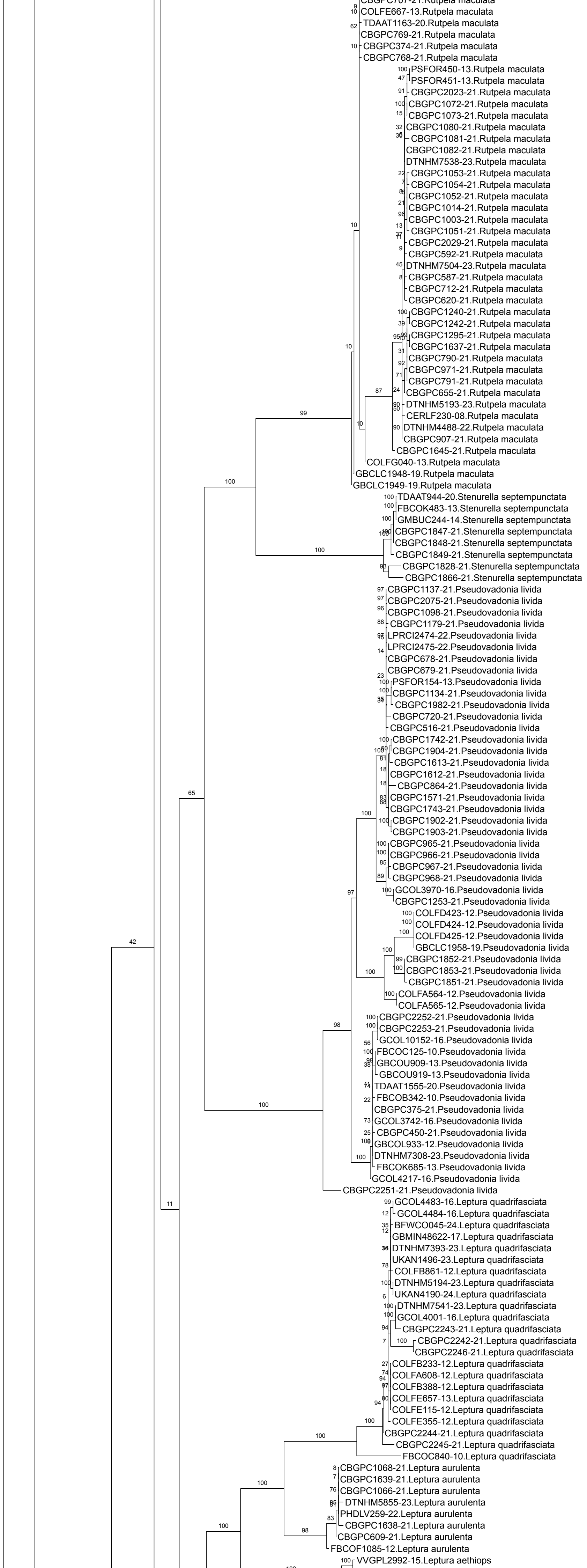
COLFE667-13.Rutpela maculata
TDAAT1163-20.Rutpela maculata
CBGPC769-21.Rutpela maculata
CBGPC374-21.Rutpela maculata
CBGPC768-21.Rutpela maculata
PSFOR450-13.Rutpela maculata
PSFOR451-13.Rutpela maculata
CBGPC2023-21.Rutpela maculata
CBGPC1072-21.Rutpela maculata
CBGPC1073-21.Rutpela maculata
CBGPC1080-21.Rutpela maculata
CBGPC1081-21.Rutpela maculata
CBGPC1082-21.Rutpela maculata
DTNHM7538-23.Rutpela maculata
CBGPC1053-21.Rutpela maculata
CBGPC1054-21.Rutpela maculata
CBGPC1052-21.Rutpela maculata
CBGPC1014-21.Rutpela maculata
CBGPC1003-21.Rutpela maculata
CBGPC1051-21.Rutpela maculata
CBGPC2029-21.Rutpela maculata
CBGPC592-21.Rutpela maculata
DTNHM7504-23.Rutpela maculata
CBGPC587-21.Rutpela maculata
CBGPC712-21.Rutpela maculata
CBGPC620-21.Rutpela maculata
CBGPC1240-21.Rutpela maculata
CBGPC1242-21.Rutpela maculata
CBGPC1295-21.Rutpela maculata
CBGPC1637-21.Rutpela maculata
CBGPC790-21.Rutpela maculata
CBGPC971-21.Rutpela maculata
CBGPC791-21.Rutpela maculata
CBGPC655-21.Rutpela maculata
DTNHM5193-23.Rutpela maculata
CERLF230-08.Rutpela maculata
DTNHM4488-22.Rutpela maculata
CBGPC907-21.Rutpela maculata
CBGPC1645-21.Rutpela maculata
COLFG040-13.Rutpela maculata
GBCLC1948-19.Rutpela maculata
GBCLC1949-19.Rutpela maculata
TDAAT944-20.Stenurella septempunctata
FBCOK483-13.Stenurella septempunctata
GMBUC244-14.Stenurella septempunctata
CBGPC1847-21.Stenurella septempunctata
CBGPC1848-21.Stenurella septempunctata
CBGPC1849-21.Stenurella septempunctata
CBGPC1828-21.Stenurella septempunctata
CBGPC1866-21.Stenurella septempunctata
CBGPC1137-21.Pseudovadonia livida
CBGPC2075-21.Pseudovadonia livida
CBGPC1098-21.Pseudovadonia livida
CBGPC1179-21.Pseudovadonia livida
LPRCI2474-22.Pseudovadonia livida
LPRCI2475-22.Pseudovadonia livida
CBGPC678-21.Pseudovadonia livida
CBGPC679-21.Pseudovadonia livida
PSFOR154-13.Pseudovadonia livida
CBGPC1134-21.Pseudovadonia livida
CBGPC1982-21.Pseudovadonia livida
CBGPC720-21.Pseudovadonia livida
CBGPC516-21.Pseudovadonia livida
CBGPC1742-21.Pseudovadonia livida
CBGPC1904-21.Pseudovadonia livida
CBGPC1613-21.Pseudovadonia livida
CBGPC1612-21.Pseudovadonia livida
CBGPC864-21.Pseudovadonia livida
CBGPC1571-21.Pseudovadonia livida
CBGPC1743-21.Pseudovadonia livida
CBGPC1902-21.Pseudovadonia livida
CBGPC1903-21.Pseudovadonia livida
CBGPC965-21.Pseudovadonia livida
CBGPC966-21.Pseudovadonia livida
CBGPC967-21.Pseudovadonia livida
CBGPC968-21.Pseudovadonia livida
GCOL3970-16.Pseudovadonia livida
CBGPC1253-21.Pseudovadonia livida
COLFD423-12.Pseudovadonia livida
COLFD424-12.Pseudovadonia livida
COLFD425-12.Pseudovadonia livida
GBCLC1958-19.Pseudovadonia livida
CBGPC1852-21.Pseudovadonia livida
CBGPC1853-21.Pseudovadonia livida
CBGPC1851-21.Pseudovadonia livida
COLFA564-12.Pseudovadonia livida
COLFA565-12.Pseudovadonia livida
CBGPC2252-21.Pseudovadonia livida
CBGPC2253-21.Pseudovadonia livida
GCOL10152-16.Pseudovadonia livida
FBCOC125-10.Pseudovadonia livida
GBCOU909-13.Pseudovadonia livida
GBCOU919-13.Pseudovadonia livida
TDAAT1555-20.Pseudovadonia livida
FBCOB342-10.Pseudovadonia livida
CBGPC375-21.Pseudovadonia livida
GCOL3742-16.Pseudovadonia livida
CBGPC450-21.Pseudovadonia livida
GBCOL933-12.Pseudovadonia livida
DTNHM7308-23.Pseudovadonia livida
FBCOK685-13.Pseudovadonia livida
GCOL4217-16.Pseudovadonia livida
CBGPC2251-21.Pseudovadonia livida
GCOL4483-16.Leptura quadrifasciata
GCOL4484-16.Leptura quadrifasciata
BFWCO045-24.Leptura quadrifasciata
GBMIN48622-17.Leptura quadrifasciata
DTNHM7393-23.Leptura quadrifasciata
UKAN1496-23.Leptura quadrifasciata
COLFB861-12.Leptura quadrifasciata
DTNHM5194-23.Leptura quadrifasciata
UKAN4190-24.Leptura quadrifasciata
DTNHM7541-23.Leptura quadrifasciata
GCOL4001-16.Leptura quadrifasciata
CBGPC2243-21.Leptura quadrifasciata
CBGPC2242-21.Leptura quadrifasciata
CBGPC2246-21.Leptura quadrifasciata
COLFB233-12.Leptura quadrifasciata
COLFA608-12.Leptura quadrifasciata
COLFB388-12.Leptura quadrifasciata
COLFE657-13.Leptura quadrifasciata
COLFE115-12.Leptura quadrifasciata
COLFE355-12.Leptura quadrifasciata
CBGPC2244-21.Leptura quadrifasciata
CBGPC2245-21.Leptura quadrifasciata
FBCOC840-10.Leptura quadrifasciata
CBGPC1068-21.Leptura aurulenta
CBGPC1639-21.Leptura aurulenta
CBGPC1066-21.Leptura aurulenta
DTNHM5855-23.Leptura aurulenta
PHDLV259-22.Leptura aurulenta
CBGPC1638-21.Leptura aurulenta
CBGPC609-21.Leptura aurulenta
FBCOF1085-12.Leptura aurulenta
VVGPL2992-15.Leptura aethiops

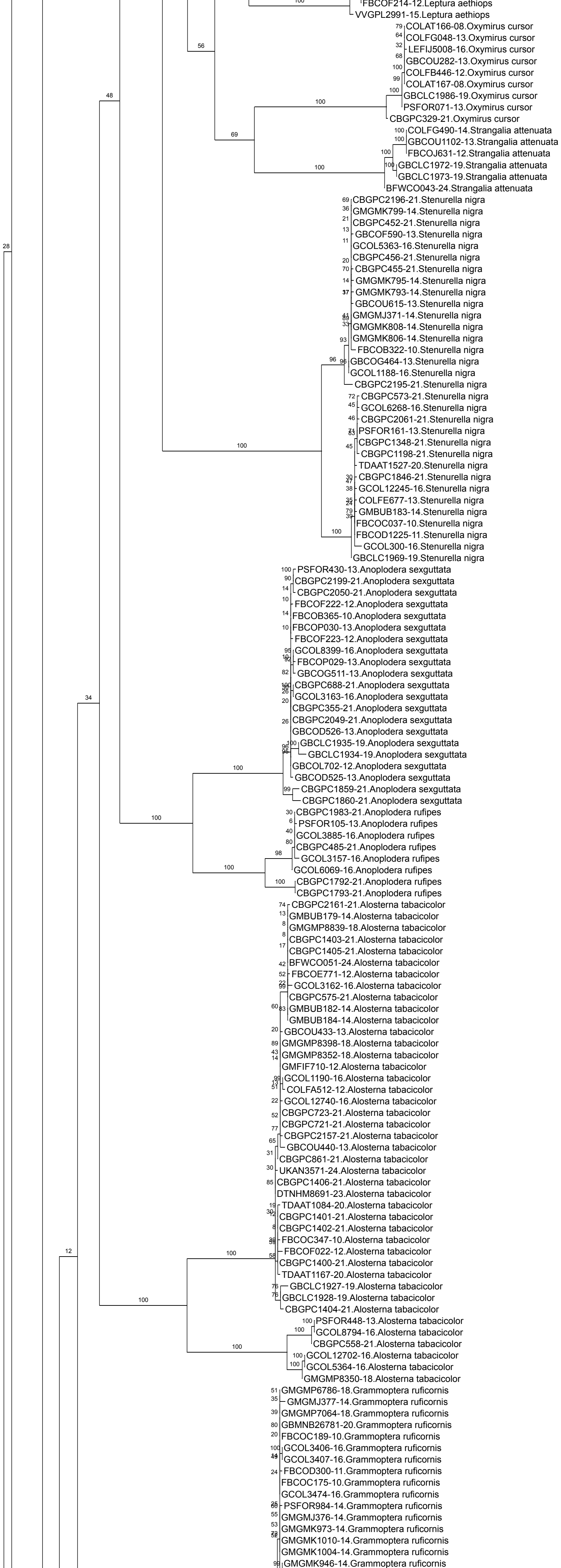
FBCOF214-12.Leptura aethiops
VVGPL2991-15.Leptura aethiops
COLAT166-08.Oxymirus cursor
COLFG048-13.Oxymirus cursor
LEFIJ5008-16.Oxymirus cursor
GBCOU282-13.Oxymirus cursor
COLFB446-12.Oxymirus cursor
COLAT167-08.Oxymirus cursor
GBCLC1986-19.Oxymirus cursor
PSFOR071-13.Oxymirus cursor
CBGPC329-21.Oxymirus cursor
COLFG490-14.Strangalia attenuata
GBCOU1102-13.Strangalia attenuata
FBCOJ631-12.Strangalia attenuata
GBCLC1972-19.Strangalia attenuata
GBCLC1973-19.Strangalia attenuata
BFWCO043-24.Strangalia attenuata
CBGPC2196-21.Stenurella nigra
GMGMK799-14.Stenurella nigra
CBGPC452-21.Stenurella nigra
GBCOF590-13.Stenurella nigra
GCOL5363-16.Stenurella nigra
CBGPC456-21.Stenurella nigra
CBGPC455-21.Stenurella nigra
GMGMK795-14.Stenurella nigra
GMGMK793-14.Stenurella nigra
GBCOU615-13.Stenurella nigra
GMGMJ371-14.Stenurella nigra
GMGMK808-14.Stenurella nigra
GMGMK806-14.Stenurella nigra
FBCOB322-10.Stenurella nigra
GBCOG464-13.Stenurella nigra
GCOL1188-16.Stenurella nigra
CBGPC2195-21.Stenurella nigra
CBGPC573-21.Stenurella nigra
GCOL6268-16.Stenurella nigra
CBGPC2061-21.Stenurella nigra
PSFOR161-13.Stenurella nigra
CBGPC1348-21.Stenurella nigra
CBGPC1198-21.Stenurella nigra
TDAAT1527-20.Stenurella nigra
CBGPC1846-21.Stenurella nigra
GCOL12245-16.Stenurella nigra
COLFE677-13.Stenurella nigra
GMBUB183-14.Stenurella nigra
FBCOC037-10.Stenurella nigra
FBCOD1225-11.Stenurella nigra
GCOL300-16.Stenurella nigra
GBCLC1969-19.Stenurella nigra
PSFOR430-13.Anoplodera sexguttata
CBGPC2199-21.Anoplodera sexguttata
CBGPC2050-21.Anoplodera sexguttata
FBCOF222-12.Anoplodera sexguttata
FBCOB365-10.Anoplodera sexguttata
FBCOP030-13.Anoplodera sexguttata
FBCOF223-12.Anoplodera sexguttata
GCOL8399-16.Anoplodera sexguttata
FBCOP029-13.Anoplodera sexguttata
GBCOG511-13.Anoplodera sexguttata
CBGPC688-21.Anoplodera sexguttata
GCOL3163-16.Anoplodera sexguttata
CBGPC355-21.Anoplodera sexguttata
CBGPC2049-21.Anoplodera sexguttata
GBCOD526-13.Anoplodera sexguttata
GBCLC1935-19.Anoplodera sexguttata
GBCLC1934-19.Anoplodera sexguttata
GBCOL702-12.Anoplodera sexguttata
GBCOD525-13.Anoplodera sexguttata
CBGPC1859-21.Anoplodera sexguttata
CBGPC1860-21.Anoplodera sexguttata
CBGPC1983-21.Anoplodera rufipes
PSFOR105-13.Anoplodera rufipes
GCOL3885-16.Anoplodera rufipes
CBGPC485-21.Anoplodera rufipes
GCOL3157-16.Anoplodera rufipes
GCOL6069-16.Anoplodera rufipes
CBGPC1792-21.Anoplodera rufipes
CBGPC1793-21.Anoplodera rufipes
CBGPC2161-21.Alosterna tabacicolor
GMBUB179-14.Alosterna tabacicolor
GMGMP8839-18.Alosterna tabacicolor
CBGPC1403-21.Alosterna tabacicolor
CBGPC1405-21.Alosterna tabacicolor
BFWCO051-24.Alosterna tabacicolor
FBCOE771-12.Alosterna tabacicolor
GCOL3162-16.Alosterna tabacicolor
CBGPC575-21.Alosterna tabacicolor
GMBUB182-14.Alosterna tabacicolor
GMBUB184-14.Alosterna tabacicolor
GBCOU433-13.Alosterna tabacicolor
GMGMP8398-18.Alosterna tabacicolor
GMGMP8352-18.Alosterna tabacicolor
GMFIF710-12.Alosterna tabacicolor
GCOL1190-16.Alosterna tabacicolor
COLFA512-12.Alosterna tabacicolor
GCOL12740-16.Alosterna tabacicolor
CBGPC723-21.Alosterna tabacicolor
CBGPC721-21.Alosterna tabacicolor
CBGPC2157-21.Alosterna tabacicolor
GBCOU440-13.Alosterna tabacicolor
CBGPC861-21.Alosterna tabacicolor
UKAN3571-24.Alosterna tabacicolor
CBGPC1406-21.Alosterna tabacicolor
DTNHM8691-23.Alosterna tabacicolor
TDAAT1084-20.Alosterna tabacicolor
CBGPC1401-21.Alosterna tabacicolor
CBGPC1402-21.Alosterna tabacicolor
FBCOC347-10.Alosterna tabacicolor
FBCOF022-12.Alosterna tabacicolor
CBGPC1400-21.Alosterna tabacicolor
TDAAT1167-20.Alosterna tabacicolor
GBCLC1927-19.Alosterna tabacicolor
GBCLC1928-19.Alosterna tabacicolor
CBGPC1404-21.Alosterna tabacicolor
PSFOR448-13.Alosterna tabacicolor
GCOL8794-16.Alosterna tabacicolor
CBGPC558-21.Alosterna tabacicolor
GCOL12702-16.Alosterna tabacicolor
GCOL5364-16.Alosterna tabacicolor
GMGMP8350-18.Alosterna tabacicolor
GMGMP6786-18.Grammoptera ruficornis
GMGMJ377-14.Grammoptera ruficornis
GMGMP7064-18.Grammoptera ruficornis
GBMNB26781-20.Grammoptera ruficornis
FBCOC189-10.Grammoptera ruficornis
GCOL3406-16.Grammoptera ruficornis
GCOL3407-16.Grammoptera ruficornis
FBCOD300-11.Grammoptera ruficornis
FBCOC175-10.Grammoptera ruficornis
GCOL3474-16.Grammoptera ruficornis
PSFOR984-14.Grammoptera ruficornis
GMGMJ376-14.Grammoptera ruficornis
GMGMK973-14.Grammoptera ruficornis
GMGMK1010-14.Grammoptera ruficornis
GMGMK1004-14.Grammoptera ruficornis
GMGMK946-14.Grammoptera ruficornis

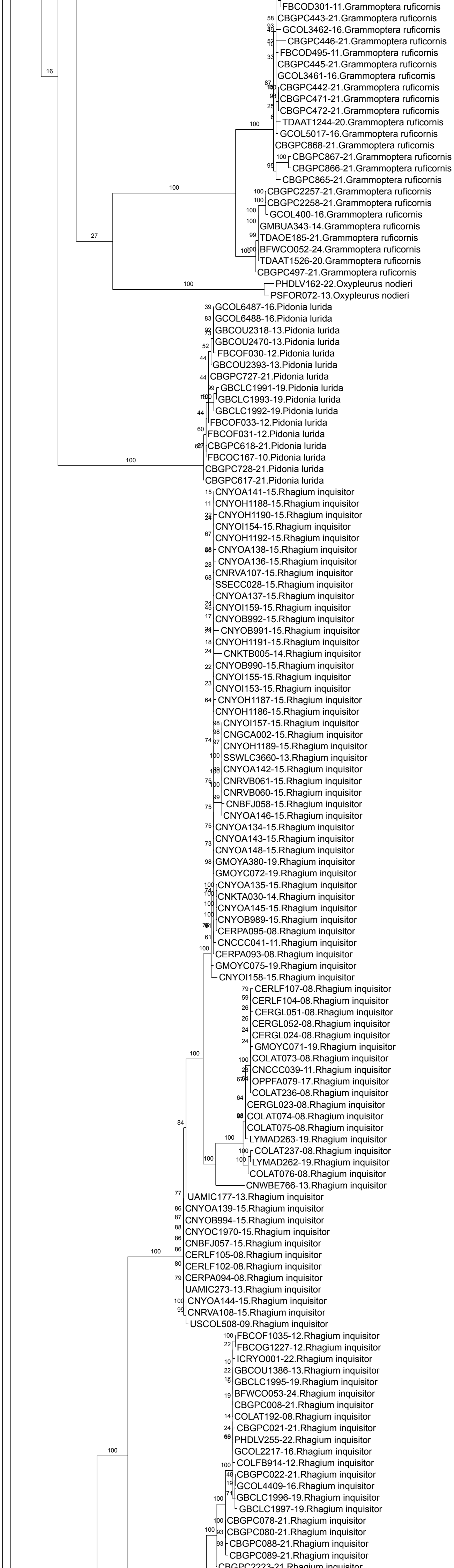
FBCOD301-11.Grammoptera ruficornis
CBGPC443-21.Grammoptera ruficornis
GCOL3462-16.Grammoptera ruficornis
CBGPC446-21.Grammoptera ruficornis
FBCOD495-11.Grammoptera ruficornis
CBGPC445-21.Grammoptera ruficornis
GCOL3461-16.Grammoptera ruficornis
CBGPC442-21.Grammoptera ruficornis
CBGPC471-21.Grammoptera ruficornis
CBGPC472-21.Grammoptera ruficornis
TDAAT1244-20.Grammoptera ruficornis
GCOL5017-16.Grammoptera ruficornis
CBGPC868-21.Grammoptera ruficornis
CBGPC867-21.Grammoptera ruficornis
CBGPC866-21.Grammoptera ruficornis
CBGPC865-21.Grammoptera ruficornis
CBGPC2257-21.Grammoptera ruficornis
CBGPC2258-21.Grammoptera ruficornis
GCOL400-16.Grammoptera ruficornis
GMBUA343-14.Grammoptera ruficornis
TDAOE185-21.Grammoptera ruficornis
BFWCO052-24.Grammoptera ruficornis
TDAAT1526-20.Grammoptera ruficornis
CBGPC497-21.Grammoptera ruficornis
PHDLV162-22.Oxypleurus nodieri
PSFOR072-13.Oxypleurus nodieri
GCOL6487-16.Pidonia lurida
GCOL6488-16.Pidonia lurida
GBCOU2318-13.Pidonia lurida
GBCOU2470-13.Pidonia lurida
FBCOF030-12.Pidonia lurida
GBCOU2393-13.Pidonia lurida
CBGPC727-21.Pidonia lurida
GBCLC1991-19.Pidonia lurida
GBCLC1993-19.Pidonia lurida
GBCLC1992-19.Pidonia lurida
FBCOF033-12.Pidonia lurida
FBCOF031-12.Pidonia lurida
CBGPC618-21.Pidonia lurida
FBCOC167-10.Pidonia lurida
CBGPC728-21.Pidonia lurida
CBGPC617-21.Pidonia lurida
CNYOA141-15.Rhagium inquisitor
CNYOH1188-15.Rhagium inquisitor
CNYOH1190-15.Rhagium inquisitor
CNYOI154-15.Rhagium inquisitor
CNYOH1192-15.Rhagium inquisitor
CNYOA138-15.Rhagium inquisitor
CNYOA136-15.Rhagium inquisitor
CNRVA107-15.Rhagium inquisitor
SSECC028-15.Rhagium inquisitor
CNYOA137-15.Rhagium inquisitor
CNYOI159-15.Rhagium inquisitor
CNYOB992-15.Rhagium inquisitor
CNYOB991-15.Rhagium inquisitor
CNYOH1191-15.Rhagium inquisitor
CNKTB005-14.Rhagium inquisitor
CNYOB990-15.Rhagium inquisitor
CNYOI155-15.Rhagium inquisitor
CNYOI153-15.Rhagium inquisitor
CNYOH1187-15.Rhagium inquisitor
CNYOH1186-15.Rhagium inquisitor
CNYOI157-15.Rhagium inquisitor
CNGCA002-15.Rhagium inquisitor
CNYOH1189-15.Rhagium inquisitor
SSWLC3660-13.Rhagium inquisitor
CNYOA142-15.Rhagium inquisitor
CNRVB061-15.Rhagium inquisitor
CNRVB060-15.Rhagium inquisitor
CNBFJ058-15.Rhagium inquisitor
CNYOA146-15.Rhagium inquisitor
CNYOA134-15.Rhagium inquisitor
CNYOA143-15.Rhagium inquisitor
CNYOA148-15.Rhagium inquisitor
GMOYA380-19.Rhagium inquisitor
GMOYC072-19.Rhagium inquisitor
CNYOA135-15.Rhagium inquisitor
CNKTA030-14.Rhagium inquisitor
CNYOA145-15.Rhagium inquisitor
CNYOB989-15.Rhagium inquisitor
CERPA095-08.Rhagium inquisitor
CNCCC041-11.Rhagium inquisitor
CERPA093-08.Rhagium inquisitor
GMOYC075-19.Rhagium inquisitor
CNYOI158-15.Rhagium inquisitor
CERLF107-08.Rhagium inquisitor
CERLF104-08.Rhagium inquisitor
CERGL051-08.Rhagium inquisitor
CERGL052-08.Rhagium inquisitor
CERGL024-08.Rhagium inquisitor
GMOYC071-19.Rhagium inquisitor
COLAT073-08.Rhagium inquisitor
CNCCC039-11.Rhagium inquisitor
OPPFA079-17.Rhagium inquisitor
COLAT236-08.Rhagium inquisitor
CERGL023-08.Rhagium inquisitor
COLAT074-08.Rhagium inquisitor
COLAT075-08.Rhagium inquisitor
LYMAD263-19.Rhagium inquisitor
COLAT237-08.Rhagium inquisitor
LYMAD262-19.Rhagium inquisitor
COLAT076-08.Rhagium inquisitor
CNWBE766-13.Rhagium inquisitor
UAMIC177-13.Rhagium inquisitor
CNYOA139-15.Rhagium inquisitor
CNYOB994-15.Rhagium inquisitor
CNYOC1970-15.Rhagium inquisitor
CNBFJ057-15.Rhagium inquisitor
CERLF105-08.Rhagium inquisitor
CERLF102-08.Rhagium inquisitor
CERPA094-08.Rhagium inquisitor
UAMIC273-13.Rhagium inquisitor
CNYOA144-15.Rhagium inquisitor
CNRVA108-15.Rhagium inquisitor
USCOL508-09.Rhagium inquisitor
FBCOF1035-12.Rhagium inquisitor
FBCOG1227-12.Rhagium inquisitor
ICRYO001-22.Rhagium inquisitor
GBCOU1386-13.Rhagium inquisitor
GBCLC1995-19.Rhagium inquisitor
BFWCO053-24.Rhagium inquisitor
CBGPC008-21.Rhagium inquisitor
COLAT192-08.Rhagium inquisitor
CBGPC021-21.Rhagium inquisitor
PHDLV255-22.Rhagium inquisitor
GCOL2217-16.Rhagium inquisitor
COLFB914-12.Rhagium inquisitor
CBGPC022-21.Rhagium inquisitor
GCOL4409-16.Rhagium inquisitor
GBCLC1996-19.Rhagium inquisitor
GBCLC1997-19.Rhagium inquisitor
CBGPC078-21.Rhagium inquisitor
CBGPC080-21.Rhagium inquisitor
CBGPC088-21.Rhagium inquisitor
CBGPC089-21.Rhagium inquisitor
CBGPC2223-21.Rhagium inquisitor

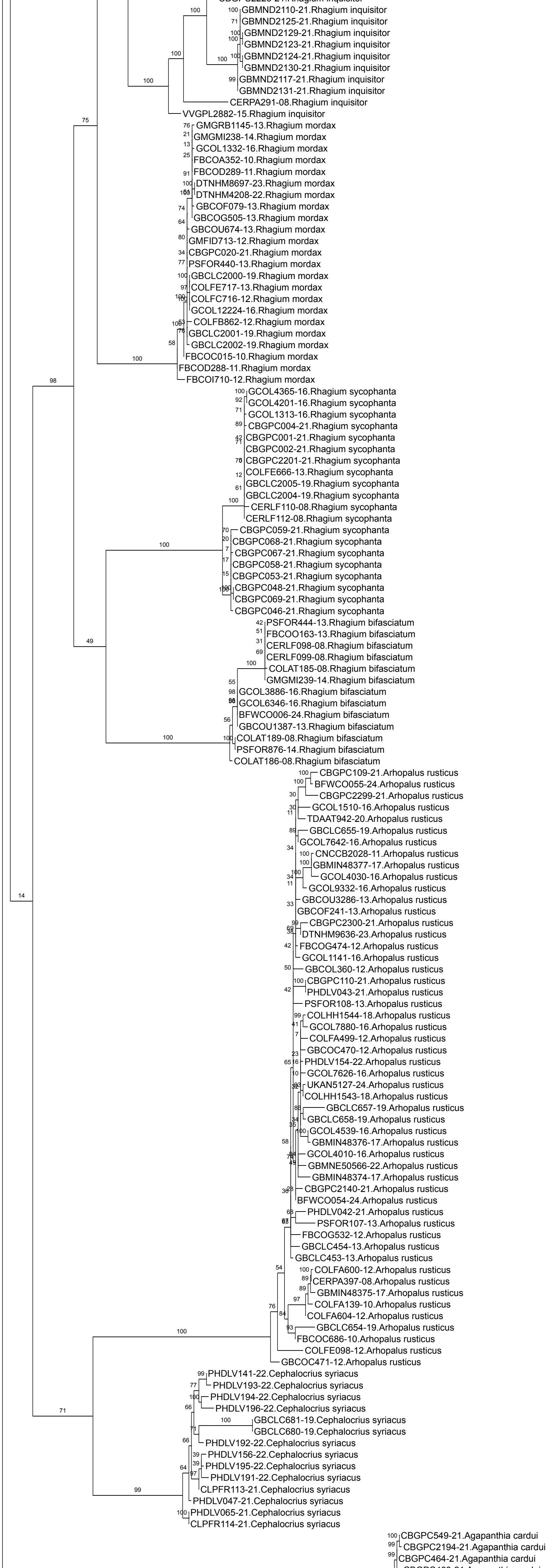
GBMND2110-21.Rhagium inquisitor
GBMND2125-21.Rhagium inquisitor
GBMND2129-21.Rhagium inquisitor
GBMND2123-21.Rhagium inquisitor
GBMND2124-21.Rhagium inquisitor
GBMND2130-21.Rhagium inquisitor
GBMND2117-21.Rhagium inquisitor
GBMND2131-21.Rhagium inquisitor
CERPA291-08.Rhagium inquisitor
VVGPL2882-15.Rhagium inquisitor
GMGRB1145-13.Rhagium mordax
GMGMI238-14.Rhagium mordax
GCOL1332-16.Rhagium mordax
FBCOA352-10.Rhagium mordax
FBCOD289-11.Rhagium mordax
DTNHM8697-23.Rhagium mordax
DTNHM4208-22.Rhagium mordax
GBCOF079-13.Rhagium mordax
GBCOG505-13.Rhagium mordax
GBCOU674-13.Rhagium mordax
GMFID713-12.Rhagium mordax
CBGPC020-21.Rhagium mordax
PSFOR440-13.Rhagium mordax
GBCLC2000-19.Rhagium mordax
COLFE717-13.Rhagium mordax
COLFC716-12.Rhagium mordax
GCOL12224-16.Rhagium mordax
COLFB862-12.Rhagium mordax
GBCLC2001-19.Rhagium mordax
GBCLC2002-19.Rhagium mordax
FBCOC015-10.Rhagium mordax
FBCOD288-11.Rhagium mordax
FBCOI710-12.Rhagium mordax
GCOL4365-16.Rhagium sycophanta
GCOL4201-16.Rhagium sycophanta
GCOL1313-16.Rhagium sycophanta
CBGPC004-21.Rhagium sycophanta
CBGPC001-21.Rhagium sycophanta
CBGPC002-21.Rhagium sycophanta
CBGPC2201-21.Rhagium sycophanta
COLFE666-13.Rhagium sycophanta
GBCLC2005-19.Rhagium sycophanta
GBCLC2004-19.Rhagium sycophanta
CERLF110-08.Rhagium sycophanta
CERLF112-08.Rhagium sycophanta
CBGPC059-21.Rhagium sycophanta
CBGPC068-21.Rhagium sycophanta
CBGPC067-21.Rhagium sycophanta
CBGPC058-21.Rhagium sycophanta
CBGPC053-21.Rhagium sycophanta
CBGPC048-21.Rhagium sycophanta
CBGPC069-21.Rhagium sycophanta
CBGPC046-21.Rhagium sycophanta
PSFOR444-13.Rhagium bifasciatum
FBCOO163-13.Rhagium bifasciatum
CERLF098-08.Rhagium bifasciatum
CERLF099-08.Rhagium bifasciatum
COLAT185-08.Rhagium bifasciatum
GMGMI239-14.Rhagium bifasciatum
GCOL3886-16.Rhagium bifasciatum
GCOL6346-16.Rhagium bifasciatum
BFWCO006-24.Rhagium bifasciatum
GBCOU1387-13.Rhagium bifasciatum
COLAT189-08.Rhagium bifasciatum
PSFOR876-14.Rhagium bifasciatum
COLAT186-08.Rhagium bifasciatum
CBGPC109-21.Arhopalus rusticus
BFWCO055-24.Arhopalus rusticus
CBGPC2299-21.Arhopalus rusticus
GCOL1510-16.Arhopalus rusticus
TDAAT942-20.Arhopalus rusticus
GBCLC655-19.Arhopalus rusticus
GCOL7642-16.Arhopalus rusticus
CNCCB2028-11.Arhopalus rusticus
GBMIN48377-17.Arhopalus rusticus
GCOL4030-16.Arhopalus rusticus
GCOL9332-16.Arhopalus rusticus
GBCOU3286-13.Arhopalus rusticus
GBCOF241-13.Arhopalus rusticus
CBGPC2300-21.Arhopalus rusticus
DTNHM9636-23.Arhopalus rusticus
FBCOG474-12.Arhopalus rusticus
GCOL1141-16.Arhopalus rusticus
GBCOL360-12.Arhopalus rusticus
CBGPC110-21.Arhopalus rusticus
PHDLV043-21.Arhopalus rusticus
PSFOR108-13.Arhopalus rusticus
COLHH1544-18.Arhopalus rusticus
GCOL7880-16.Arhopalus rusticus
COLFA499-12.Arhopalus rusticus
GBCOC470-12.Arhopalus rusticus
PHDLV154-22.Arhopalus rusticus
GCOL7626-16.Arhopalus rusticus
UKAN5127-24.Arhopalus rusticus
COLHH1543-18.Arhopalus rusticus
GBCLC657-19.Arhopalus rusticus
GBCLC658-19.Arhopalus rusticus
GCOL4539-16.Arhopalus rusticus
GBMIN48376-17.Arhopalus rusticus
GCOL4010-16.Arhopalus rusticus
GBMNE50566-22.Arhopalus rusticus
GBMIN48374-17.Arhopalus rusticus
CBGPC2140-21.Arhopalus rusticus
BFWCO054-24.Arhopalus rusticus
PHDLV042-21.Arhopalus rusticus
PSFOR107-13.Arhopalus rusticus
FBCOG532-12.Arhopalus rusticus
GBCLC454-13.Arhopalus rusticus
GBCLC453-13.Arhopalus rusticus
COLFA600-12.Arhopalus rusticus
CERPA397-08.Arhopalus rusticus
GBMIN48375-17.Arhopalus rusticus
COLFA139-10.Arhopalus rusticus
COLFA604-12.Arhopalus rusticus
GBCLC654-19.Arhopalus rusticus
FBCOC686-10.Arhopalus rusticus
COLFE098-12.Arhopalus rusticus
GBCOC471-12.Arhopalus rusticus
PHDLV141-22.Cephalocrius syriacus
PHDLV193-22.Cephalocrius syriacus
PHDLV194-22.Cephalocrius syriacus
PHDLV196-22.Cephalocrius syriacus
GBCLC681-19.Cephalocrius syriacus
GBCLC680-19.Cephalocrius syriacus
PHDLV192-22.Cephalocrius syriacus
PHDLV156-22.Cephalocrius syriacus
PHDLV195-22.Cephalocrius syriacus
PHDLV191-22.Cephalocrius syriacus
CLPFR113-21.Cephalocrius syriacus
PHDLV047-21.Cephalocrius syriacus
PHDLV065-21.Cephalocrius syriacus
CLPFR114-21.Cephalocrius syriacus
CBGPC549-21.Agapanthia cardui
CBGPC2194-21.Agapanthia cardui
CBGPC464-21.Agapanthia cardui

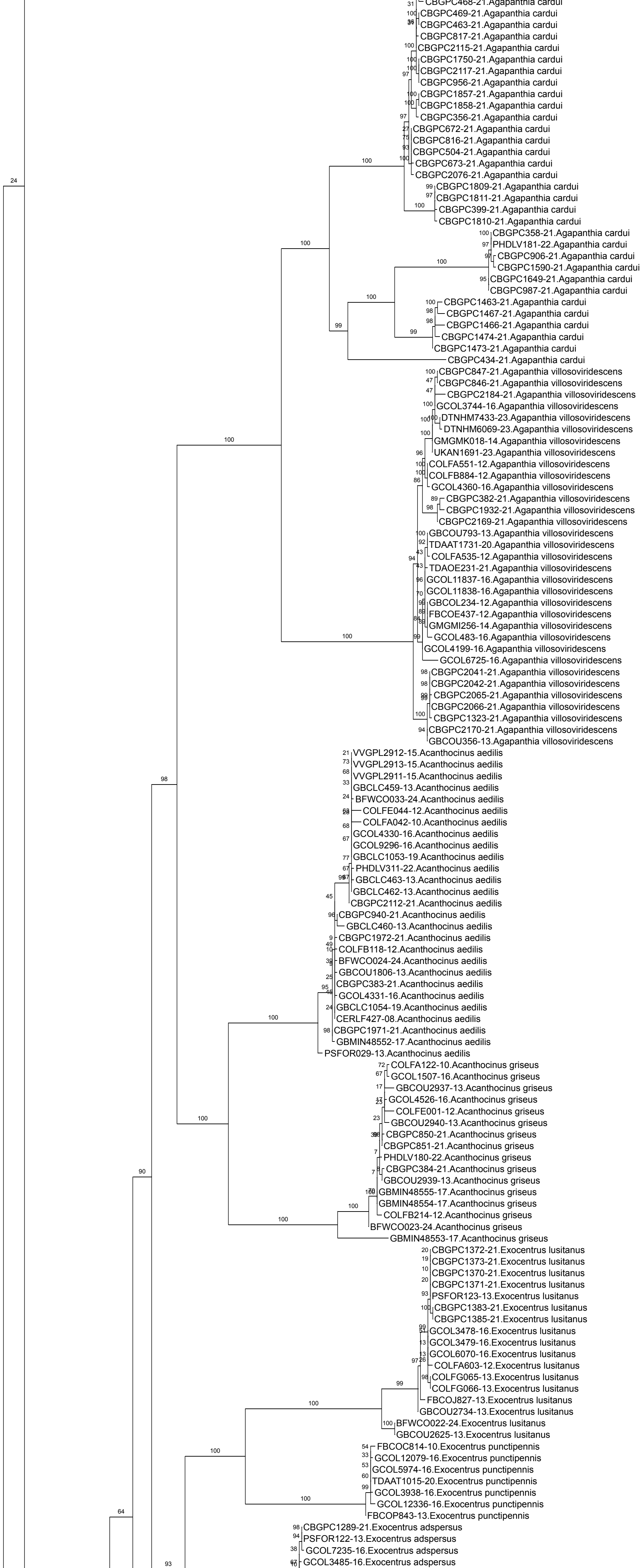
CBGPC468-21.Agapanthia cardui
CBGPC469-21.Agapanthia cardui
CBGPC463-21.Agapanthia cardui
CBGPC817-21.Agapanthia cardui
CBGPC2115-21.Agapanthia cardui
CBGPC1750-21.Agapanthia cardui
CBGPC2117-21.Agapanthia cardui
CBGPC956-21.Agapanthia cardui
CBGPC1857-21.Agapanthia cardui
CBGPC1858-21.Agapanthia cardui
CBGPC356-21.Agapanthia cardui
CBGPC672-21.Agapanthia cardui
CBGPC816-21.Agapanthia cardui
CBGPC504-21.Agapanthia cardui
CBGPC673-21.Agapanthia cardui
CBGPC2076-21.Agapanthia cardui
CBGPC1809-21.Agapanthia cardui
CBGPC1811-21.Agapanthia cardui
CBGPC399-21.Agapanthia cardui
CBGPC1810-21.Agapanthia cardui
CBGPC358-21.Agapanthia cardui
PHDLV181-22.Agapanthia cardui
CBGPC906-21.Agapanthia cardui
CBGPC1590-21.Agapanthia cardui
CBGPC1649-21.Agapanthia cardui
CBGPC987-21.Agapanthia cardui
CBGPC1463-21.Agapanthia cardui
CBGPC1467-21.Agapanthia cardui
CBGPC1466-21.Agapanthia cardui
CBGPC1474-21.Agapanthia cardui
CBGPC1473-21.Agapanthia cardui
CBGPC434-21.Agapanthia cardui
CBGPC847-21.Agapanthia villosoviridescens
CBGPC846-21.Agapanthia villosoviridescens
CBGPC2184-21.Agapanthia villosoviridescens
GCOL3744-16.Agapanthia villosoviridescens
DTNHM7433-23.Agapanthia villosoviridescens
DTNHM6069-23.Agapanthia villosoviridescens
GMGMK018-14.Agapanthia villosoviridescens
UKAN1691-23.Agapanthia villosoviridescens
COLFA551-12.Agapanthia villosoviridescens
COLFB884-12.Agapanthia villosoviridescens
GCOL4360-16.Agapanthia villosoviridescens
CBGPC382-21.Agapanthia villosoviridescens
CBGPC1932-21.Agapanthia villosoviridescens
CBGPC2169-21.Agapanthia villosoviridescens
GBCOU793-13.Agapanthia villosoviridescens
TDAAT1731-20.Agapanthia villosoviridescens
COLFA535-12.Agapanthia villosoviridescens
TDAOE231-21.Agapanthia villosoviridescens
GCOL11837-16.Agapanthia villosoviridescens
GCOL11838-16.Agapanthia villosoviridescens
GBCOL234-12.Agapanthia villosoviridescens
FBCOE437-12.Agapanthia villosoviridescens
GMGMI256-14.Agapanthia villosoviridescens
GCOL483-16.Agapanthia villosoviridescens
GCOL4199-16.Agapanthia villosoviridescens
GCOL6725-16.Agapanthia villosoviridescens
CBGPC2041-21.Agapanthia villosoviridescens
CBGPC2042-21.Agapanthia villosoviridescens
CBGPC2065-21.Agapanthia villosoviridescens
CBGPC2066-21.Agapanthia villosoviridescens
CBGPC1323-21.Agapanthia villosoviridescens
CBGPC2170-21.Agapanthia villosoviridescens
GBCOU356-13.Agapanthia villosoviridescens
VVGPL2912-15.Acanthocinus aedilis
VVGPL2913-15.Acanthocinus aedilis
VVGPL2911-15.Acanthocinus aedilis
GBCLC459-13.Acanthocinus aedilis
BFWCO033-24.Acanthocinus aedilis
COLFE044-12.Acanthocinus aedilis
COLFA042-10.Acanthocinus aedilis
GCOL4330-16.Acanthocinus aedilis
GCOL9296-16.Acanthocinus aedilis
GBCLC1053-19.Acanthocinus aedilis
PHDLV311-22.Acanthocinus aedilis
GBCLC463-13.Acanthocinus aedilis
GBCLC462-13.Acanthocinus aedilis
CBGPC2112-21.Acanthocinus aedilis
CBGPC940-21.Acanthocinus aedilis
GBCLC460-13.Acanthocinus aedilis
CBGPC1972-21.Acanthocinus aedilis
COLFB118-12.Acanthocinus aedilis
BFWCO024-24.Acanthocinus aedilis
GBCOU1806-13.Acanthocinus aedilis
CBGPC383-21.Acanthocinus aedilis
GCOL4331-16.Acanthocinus aedilis
GBCLC1054-19.Acanthocinus aedilis
CERLF427-08.Acanthocinus aedilis
CBGPC1971-21.Acanthocinus aedilis
GBMIN48552-17.Acanthocinus aedilis
PSFOR029-13.Acanthocinus aedilis
COLFA122-10.Acanthocinus griseus
GCOL1507-16.Acanthocinus griseus
GBCOU2937-13.Acanthocinus griseus
GCOL4526-16.Acanthocinus griseus
COLFE001-12.Acanthocinus griseus
GBCOU2940-13.Acanthocinus griseus
CBGPC850-21.Acanthocinus griseus
CBGPC851-21.Acanthocinus griseus
PHDLV180-22.Acanthocinus griseus
CBGPC384-21.Acanthocinus griseus
GBCOU2939-13.Acanthocinus griseus
GBMIN48555-17.Acanthocinus griseus
GBMIN48554-17.Acanthocinus griseus
COLFB214-12.Acanthocinus griseus
BFWCO023-24.Acanthocinus griseus
GBMIN48553-17.Acanthocinus griseus
CBGPC1372-21.Exocentrus lusitanus
CBGPC1373-21.Exocentrus lusitanus
CBGPC1370-21.Exocentrus lusitanus
CBGPC1371-21.Exocentrus lusitanus
PSFOR123-13.Exocentrus lusitanus
CBGPC1383-21.Exocentrus lusitanus
CBGPC1385-21.Exocentrus lusitanus
GCOL3478-16.Exocentrus lusitanus
GCOL3479-16.Exocentrus lusitanus
GCOL6070-16.Exocentrus lusitanus
COLFA603-12.Exocentrus lusitanus
COLFG065-13.Exocentrus lusitanus
COLFG066-13.Exocentrus lusitanus
FBCOJ827-13.Exocentrus lusitanus
GBCOU2734-13.Exocentrus lusitanus
BFWCO022-24.Exocentrus lusitanus
GBCOU2625-13.Exocentrus lusitanus
FBCOC814-10.Exocentrus punctipennis
GCOL12079-16.Exocentrus punctipennis
GCOL5974-16.Exocentrus punctipennis
TDAAT1015-20.Exocentrus punctipennis
GCOL3938-16.Exocentrus punctipennis
GCOL12336-16.Exocentrus punctipennis
FBCOP843-13.Exocentrus punctipennis
CBGPC1289-21.Exocentrus adspersus
PSFOR122-13.Exocentrus adspersus
GCOL7235-16.Exocentrus adspersus
GCOL3485-16.Exocentrus adspersus

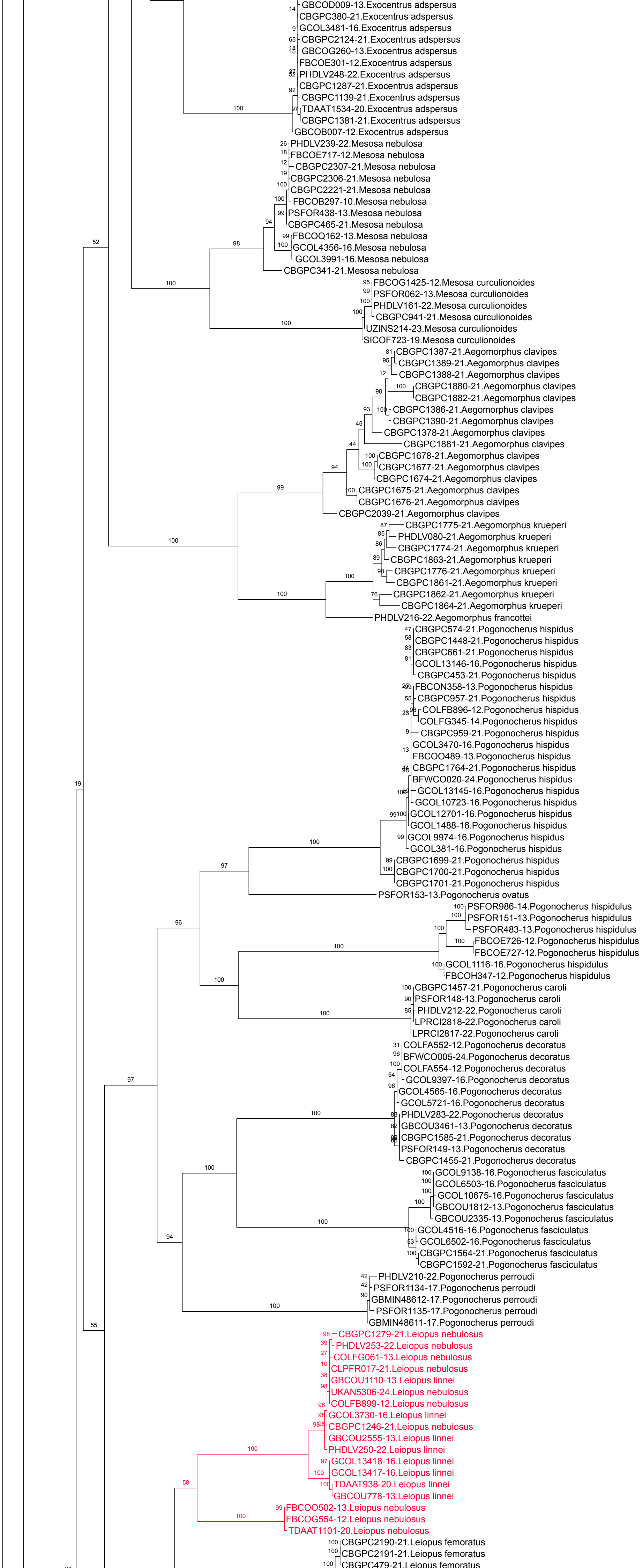
GBCOD009-13.Exocentrus adspersus
CBGPC380-21.Exocentrus adspersus
GCOL3481-16.Exocentrus adspersus
CBGPC2124-21.Exocentrus adspersus
GBCOG260-13.Exocentrus adspersus
FBCOE301-12.Exocentrus adspersus
PHDLV248-22.Exocentrus adspersus
CBGPC1287-21.Exocentrus adspersus
CBGPC1139-21.Exocentrus adspersus
TDAAT1534-20.Exocentrus adspersus
CBGPC1381-21.Exocentrus adspersus
GBCOB007-12.Exocentrus adspersus
PHDLV239-22.Mesosa nebulosa
FBCOE717-12.Mesosa nebulosa
CBGPC2307-21.Mesosa nebulosa
CBGPC2306-21.Mesosa nebulosa
CBGPC2221-21.Mesosa nebulosa
FBCOB297-10.Mesosa nebulosa
PSFOR438-13.Mesosa nebulosa
CBGPC465-21.Mesosa nebulosa
FBCOQ162-13.Mesosa nebulosa
GCOL4356-16.Mesosa nebulosa
GCOL3991-16.Mesosa nebulosa
CBGPC341-21.Mesosa nebulosa
FBCOG1425-12.Mesosa curculionoides
PSFOR062-13.Mesosa curculionoides
PHDLV161-22.Mesosa curculionoides
CBGPC941-21.Mesosa curculionoides
UZINS214-23.Mesosa curculionoides
SICOF723-19.Mesosa curculionoides
CBGPC1387-21.Aegomorphus clavipes
CBGPC1389-21.Aegomorphus clavipes
CBGPC1388-21.Aegomorphus clavipes
CBGPC1880-21.Aegomorphus clavipes
CBGPC1882-21.Aegomorphus clavipes
CBGPC1386-21.Aegomorphus clavipes
CBGPC1390-21.Aegomorphus clavipes
CBGPC1378-21.Aegomorphus clavipes
CBGPC1881-21.Aegomorphus clavipes
CBGPC1678-21.Aegomorphus clavipes
CBGPC1677-21.Aegomorphus clavipes
CBGPC1674-21.Aegomorphus clavipes
CBGPC1675-21.Aegomorphus clavipes
CBGPC1676-21.Aegomorphus clavipes
CBGPC2039-21.Aegomorphus clavipes
CBGPC1775-21.Aegomorphus krueperi
PHDLV080-21.Aegomorphus krueperi
CBGPC1774-21.Aegomorphus krueperi
CBGPC1863-21.Aegomorphus krueperi
CBGPC1776-21.Aegomorphus krueperi
CBGPC1861-21.Aegomorphus krueperi
CBGPC1862-21.Aegomorphus krueperi
CBGPC1864-21.Aegomorphus krueperi
PHDLV216-22.Aegomorphus francottei
CBGPC574-21.Pogonocherus hispidus
CBGPC1448-21.Pogonocherus hispidus
CBGPC661-21.Pogonocherus hispidus
GCOL13146-16.Pogonocherus hispidus
CBGPC453-21.Pogonocherus hispidus
FBCON358-13.Pogonocherus hispidus
CBGPC957-21.Pogonocherus hispidus
COLFB896-12.Pogonocherus hispidus
COLFG345-14.Pogonocherus hispidus
CBGPC959-21.Pogonocherus hispidus
GCOL3470-16.Pogonocherus hispidus
FBCOO489-13.Pogonocherus hispidus
CBGPC1764-21.Pogonocherus hispidus
BFWCO020-24.Pogonocherus hispidus
GCOL13145-16.Pogonocherus hispidus
GCOL10723-16.Pogonocherus hispidus
GCOL12701-16.Pogonocherus hispidus
GCOL1488-16.Pogonocherus hispidus
GCOL9974-16.Pogonocherus hispidus
GCOL381-16.Pogonocherus hispidus
CBGPC1699-21.Pogonocherus hispidus
CBGPC1700-21.Pogonocherus hispidus
CBGPC1701-21.Pogonocherus hispidus
PSFOR153-13.Pogonocherus ovatus
PSFOR986-14.Pogonocherus hispidulus
PSFOR151-13.Pogonocherus hispidulus
PSFOR483-13.Pogonocherus hispidulus
FBCOE726-12.Pogonocherus hispidulus
FBCOE727-12.Pogonocherus hispidulus
GCOL1116-16.Pogonocherus hispidulus
FBCOH347-12.Pogonocherus hispidulus
CBGPC1457-21.Pogonocherus caroli
PSFOR148-13.Pogonocherus caroli
PHDLV212-22.Pogonocherus caroli
LPRCI2818-22.Pogonocherus caroli
LPRCI2817-22.Pogonocherus caroli
COLFA552-12.Pogonocherus decoratus
BFWCO005-24.Pogonocherus decoratus
COLFA554-12.Pogonocherus decoratus
GCOL9397-16.Pogonocherus decoratus
GCOL4565-16.Pogonocherus decoratus
GCOL5721-16.Pogonocherus decoratus
PHDLV283-22.Pogonocherus decoratus
GBCOU3461-13.Pogonocherus decoratus
CBGPC1585-21.Pogonocherus decoratus
PSFOR149-13.Pogonocherus decoratus
CBGPC1455-21.Pogonocherus decoratus
GCOL9138-16.Pogonocherus fasciculatus
GCOL6503-16.Pogonocherus fasciculatus
GCOL10675-16.Pogonocherus fasciculatus
GBCOU1812-13.Pogonocherus fasciculatus
GBCOU2335-13.Pogonocherus fasciculatus
GCOL4516-16.Pogonocherus fasciculatus
GCOL6502-16.Pogonocherus fasciculatus
CBGPC1564-21.Pogonocherus fasciculatus
CBGPC1592-21.Pogonocherus fasciculatus
PHDLV210-22.Pogonocherus perroudi
PSFOR1134-17.Pogonocherus perroudi
GBMIN48612-17.Pogonocherus perroudi
PSFOR1135-17.Pogonocherus perroudi
GBMIN48611-17.Pogonocherus perroudi
CBGPC1279-21.Leiopus nebulosus
PHDLV253-22.Leiopus nebulosus
COLFG061-13.Leiopus nebulosus
CLPFR017-21.Leiopus nebulosus
GBCOU1110-13.Leiopus linnei
UKAN5306-24.Leiopus nebulosus
COLFB899-12.Leiopus nebulosus
GCOL3730-16.Leiopus linnei
CBGPC1246-21.Leiopus nebulosus
GBCOU2555-13.Leiopus linnei
PHDLV250-22.Leiopus linnei
GCOL13418-16.Leiopus linnei
GCOL13417-16.Leiopus linnei
TDAAT938-20.Leiopus linnei
GBCOU778-13.Leiopus linnei
FBCOO502-13.Leiopus nebulosus
FBCOG554-12.Leiopus nebulosus
TDAAT1101-20.Leiopus nebulosus
CBGPC2190-21.Leiopus femoratus
CBGPC2191-21.Leiopus femoratus
CBGPC479-21.Leiopus femoratus

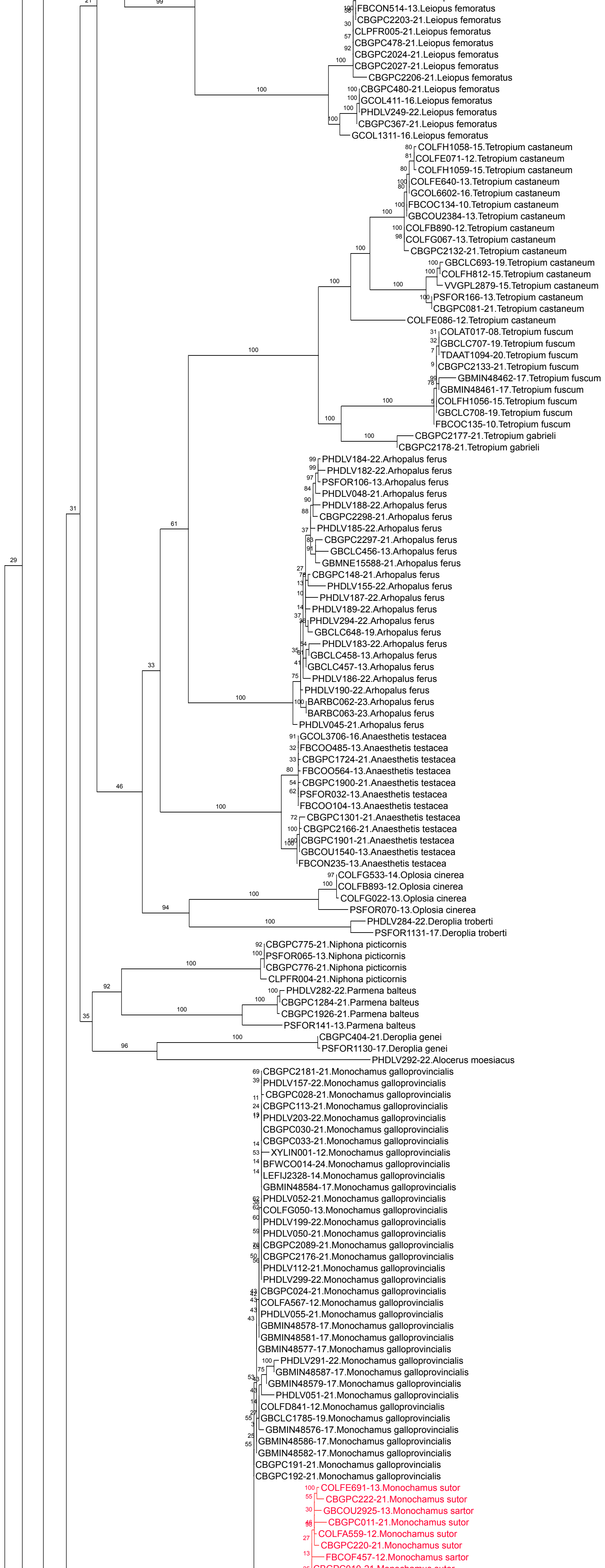
FBCON514-13.Leiopus femoratus
CBGPC2203-21.Leiopus femoratus
CLPFR005-21.Leiopus femoratus
CBGPC478-21.Leiopus femoratus
CBGPC2024-21.Leiopus femoratus
CBGPC2027-21.Leiopus femoratus
CBGPC2206-21.Leiopus femoratus
CBGPC480-21.Leiopus femoratus
GCOL411-16.Leiopus femoratus
PHDLV249-22.Leiopus femoratus
CBGPC367-21.Leiopus femoratus
GCOL1311-16.Leiopus femoratus
COLFH1058-15.Tetropium castaneum
COLFE071-12.Tetropium castaneum
COLFH1059-15.Tetropium castaneum
COLFE640-13.Tetropium castaneum
GCOL6602-16.Tetropium castaneum
FBCOC134-10.Tetropium castaneum
GBCOU2384-13.Tetropium castaneum
COLFB890-12.Tetropium castaneum
COLFG067-13.Tetropium castaneum
CBGPC2132-21.Tetropium castaneum
GBCLC693-19.Tetropium castaneum
COLFH812-15.Tetropium castaneum
VVGPL2879-15.Tetropium castaneum
PSFOR166-13.Tetropium castaneum
CBGPC081-21.Tetropium castaneum
COLFE086-12.Tetropium castaneum
COLAT017-08.Tetropium fuscum
GBCLC707-19.Tetropium fuscum
TDAAT1094-20.Tetropium fuscum
CBGPC2133-21.Tetropium fuscum
GBMIN48462-17.Tetropium fuscum
GBMIN48461-17.Tetropium fuscum
COLFH1056-15.Tetropium fuscum
GBCLC708-19.Tetropium fuscum
FBCOC135-10.Tetropium fuscum
CBGPC2177-21.Tetropium gabrieli
CBGPC2178-21.Tetropium gabrieli
PHDLV184-22.Arhopalus ferus
PHDLV182-22.Arhopalus ferus
PSFOR106-13.Arhopalus ferus
PHDLV048-21.Arhopalus ferus
PHDLV188-22.Arhopalus ferus
CBGPC2298-21.Arhopalus ferus
PHDLV185-22.Arhopalus ferus
CBGPC2297-21.Arhopalus ferus
GBCLC456-13.Arhopalus ferus
GBMNE15588-21.Arhopalus ferus
CBGPC148-21.Arhopalus ferus
PHDLV155-22.Arhopalus ferus
PHDLV187-22.Arhopalus ferus
PHDLV189-22.Arhopalus ferus
PHDLV294-22.Arhopalus ferus
GBCLC648-19.Arhopalus ferus
PHDLV183-22.Arhopalus ferus
GBCLC458-13.Arhopalus ferus
GBCLC457-13.Arhopalus ferus
PHDLV186-22.Arhopalus ferus
PHDLV190-22.Arhopalus ferus
BARBC062-23.Arhopalus ferus
BARBC063-23.Arhopalus ferus
PHDLV045-21.Arhopalus ferus
GCOL3706-16.Anaesthetis testacea
FBCOO485-13.Anaesthetis testacea
CBGPC1724-21.Anaesthetis testacea
FBCOO564-13.Anaesthetis testacea
CBGPC1900-21.Anaesthetis testacea
PSFOR032-13.Anaesthetis testacea
FBCOO104-13.Anaesthetis testacea
CBGPC1301-21.Anaesthetis testacea
CBGPC2166-21.Anaesthetis testacea
CBGPC1901-21.Anaesthetis testacea
GBCOU1540-13.Anaesthetis testacea
FBCON235-13.Anaesthetis testacea
COLFG533-14.Oplosia cinerea
COLFB893-12.Oplosia cinerea
COLFG022-13.Oplosia cinerea
PSFOR070-13.Oplosia cinerea
PHDLV284-22.Deroplia troberti
PSFOR1131-17.Deroplia troberti
CBGPC775-21.Niphona picticornis
PSFOR065-13.Niphona picticornis
CBGPC776-21.Niphona picticornis
CLPFR004-21.Niphona picticornis
PHDLV282-22.Parmena balteus
CBGPC1284-21.Parmena balteus
CBGPC1926-21.Parmena balteus
PSFOR141-13.Parmena balteus
CBGPC404-21.Deroplia genei
PSFOR1130-17.Deroplia genei
PHDLV292-22.Alocerus moesiacus
CBGPC2181-21.Monochamus galloprovincialis
PHDLV157-22.Monochamus galloprovincialis
CBGPC028-21.Monochamus galloprovincialis
CBGPC113-21.Monochamus galloprovincialis
PHDLV203-22.Monochamus galloprovincialis
CBGPC030-21.Monochamus galloprovincialis
CBGPC033-21.Monochamus galloprovincialis
XYLIN001-12.Monochamus galloprovincialis
BFWCO014-24.Monochamus galloprovincialis
LEFIJ2328-14.Monochamus galloprovincialis
GBMIN48584-17.Monochamus galloprovincialis
PHDLV052-21.Monochamus galloprovincialis
COLFG050-13.Monochamus galloprovincialis
PHDLV199-22.Monochamus galloprovincialis
PHDLV050-21.Monochamus galloprovincialis
CBGPC2089-21.Monochamus galloprovincialis
CBGPC2176-21.Monochamus galloprovincialis
PHDLV112-21.Monochamus galloprovincialis
PHDLV299-22.Monochamus galloprovincialis
CBGPC024-21.Monochamus galloprovincialis
COLFA567-12.Monochamus galloprovincialis
PHDLV055-21.Monochamus galloprovincialis
GBMIN48578-17.Monochamus galloprovincialis
GBMIN48581-17.Monochamus galloprovincialis
GBMIN48577-17.Monochamus galloprovincialis
PHDLV291-22.Monochamus galloprovincialis
GBMIN48587-17.Monochamus galloprovincialis
GBMIN48579-17.Monochamus galloprovincialis
PHDLV051-21.Monochamus galloprovincialis
COLFD841-12.Monochamus galloprovincialis
GBCLC1785-19.Monochamus galloprovincialis
GBMIN48576-17.Monochamus galloprovincialis
GBMIN48586-17.Monochamus galloprovincialis
GBMIN48582-17.Monochamus galloprovincialis
CBGPC191-21.Monochamus galloprovincialis
CBGPC192-21.Monochamus galloprovincialis
COLFE691-13.Monochamus sutor
CBGPC222-21.Monochamus sutor
GBCOU2925-13.Monochamus sartor
CBGPC011-21.Monochamus sutor
COLFA559-12.Monochamus sutor
CBGPC220-21.Monochamus sutor
FBCOF457-12.Monochamus sartor

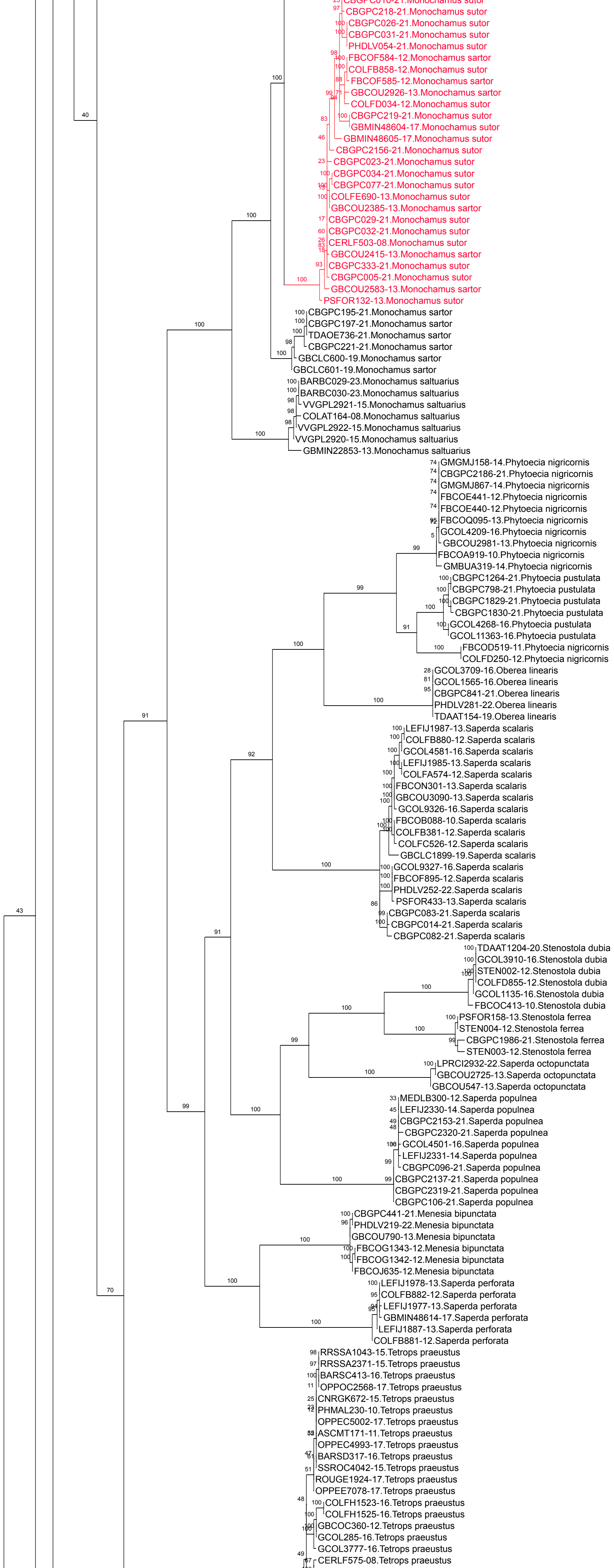
CBGPC218-21.Monochamus sutor
CBGPC026-21.Monochamus sutor
CBGPC031-21.Monochamus sutor
PHDLV054-21.Monochamus sutor
FBCOF584-12.Monochamus sartor
COLFB858-12.Monochamus sutor
FBCOF585-12.Monochamus sartor
GBCOU2926-13.Monochamus sartor
COLFD034-12.Monochamus sutor
CBGPC219-21.Monochamus sutor
GBMIN48604-17.Monochamus sutor
GBMIN48605-17.Monochamus sutor
CBGPC2156-21.Monochamus sutor
CBGPC023-21.Monochamus sutor
CBGPC034-21.Monochamus sutor
CBGPC077-21.Monochamus sutor
COLFE690-13.Monochamus sutor
GBCOU2385-13.Monochamus sartor
CBGPC029-21.Monochamus sutor
CBGPC032-21.Monochamus sutor
CERLF503-08.Monochamus sutor
GBCOU2415-13.Monochamus sartor
CBGPC333-21.Monochamus sutor
CBGPC005-21.Monochamus sutor
GBCOU2583-13.Monochamus sartor
PSFOR132-13.Monochamus sutor
CBGPC195-21.Monochamus sartor
CBGPC197-21.Monochamus sartor
TDAOE736-21.Monochamus sartor
CBGPC221-21.Monochamus sartor
GBCLC600-19.Monochamus sartor
GBCLC601-19.Monochamus sartor
BARBC029-23.Monochamus saltuarius
BARBC030-23.Monochamus saltuarius
VVGPL2921-15.Monochamus saltuarius
COLAT164-08.Monochamus saltuarius
VVGPL2922-15.Monochamus saltuarius
VVGPL2920-15.Monochamus saltuarius
GBMIN22853-13.Monochamus saltuarius
GMGMJ158-14.Phytoecia nigricornis
CBGPC2186-21.Phytoecia nigricornis
GMGMJ867-14.Phytoecia nigricornis
FBCOE441-12.Phytoecia nigricornis
FBCOE440-12.Phytoecia nigricornis
FBCOQ095-13.Phytoecia nigricornis
GCOL4209-16.Phytoecia nigricornis
GBCOU2981-13.Phytoecia nigricornis
FBCOA919-10.Phytoecia nigricornis
GMBUA319-14.Phytoecia nigricornis
CBGPC1264-21.Phytoecia pustulata
CBGPC798-21.Phytoecia pustulata
CBGPC1829-21.Phytoecia pustulata
CBGPC1830-21.Phytoecia pustulata
GCOL4268-16.Phytoecia pustulata
GCOL11363-16.Phytoecia pustulata
FBCOD519-11.Phytoecia nigricornis
COLFD250-12.Phytoecia nigricornis
GCOL3709-16.Oberea linearis
GCOL1565-16.Oberea linearis
CBGPC841-21.Oberea linearis
PHDLV281-22.Oberea linearis
TDAAT154-19.Oberea linearis
LEFIJ1987-13.Saperda scalaris
COLFB880-12.Saperda scalaris
GCOL4581-16.Saperda scalaris
LEFIJ1985-13.Saperda scalaris
COLFA574-12.Saperda scalaris
FBCON301-13.Saperda scalaris
GBCOU3090-13.Saperda scalaris
GCOL9326-16.Saperda scalaris
FBCOB088-10.Saperda scalaris
COLFB381-12.Saperda scalaris
COLFC526-12.Saperda scalaris
GBCLC1899-19.Saperda scalaris
GCOL9327-16.Saperda scalaris
FBCOF895-12.Saperda scalaris
PHDLV252-22.Saperda scalaris
PSFOR433-13.Saperda scalaris
CBGPC083-21.Saperda scalaris
CBGPC014-21.Saperda scalaris
CBGPC082-21.Saperda scalaris
TDAAT1204-20.Stenostola dubia
GCOL3910-16.Stenostola dubia
STEN002-12.Stenostola dubia
COLFD855-12.Stenostola dubia
GCOL1135-16.Stenostola dubia
FBCOC413-10.Stenostola dubia
PSFOR158-13.Stenostola ferrea
STEN004-12.Stenostola ferrea
CBGPC1986-21.Stenostola ferrea
STEN003-12.Stenostola ferrea
LPRCI2932-22.Saperda octopunctata
GBCOU2725-13.Saperda octopunctata
GBCOU547-13.Saperda octopunctata
MEDLB300-12.Saperda populnea
LEFIJ2330-14.Saperda populnea
CBGPC2153-21.Saperda populnea
CBGPC2320-21.Saperda populnea
GCOL4501-16.Saperda populnea
LEFIJ2331-14.Saperda populnea
CBGPC096-21.Saperda populnea
CBGPC2137-21.Saperda populnea
CBGPC2319-21.Saperda populnea
CBGPC106-21.Saperda populnea
CBGPC441-21.Menesia bipunctata
PHDLV219-22.Menesia bipunctata
GBCOU790-13.Menesia bipunctata
FBCOG1343-12.Menesia bipunctata
FBCOG1342-12.Menesia bipunctata
FBCOJ635-12.Menesia bipunctata
LEFIJ1978-13.Saperda perforata
COLFB882-12.Saperda perforata
LEFIJ1977-13.Saperda perforata
GBMIN48614-17.Saperda perforata
LEFIJ1887-13.Saperda perforata
COLFB881-12.Saperda perforata
RRSSA1043-15.Tetrops praeustus
RRSSA2371-15.Tetrops praeustus
BARSC413-16.Tetrops praeustus
OPPOC2568-17.Tetrops praeustus
CNRGK672-15.Tetrops praeustus
PHMAL230-10.Tetrops praeustus
OPPEC5002-17.Tetrops praeustus
ASCMT171-11.Tetrops praeustus
OPPEC4993-17.Tetrops praeustus
BARSD317-16.Tetrops praeustus
SSROC4042-15.Tetrops praeustus
ROUGE1924-17.Tetrops praeustus
OPPEE7078-17.Tetrops praeustus
COLFH1523-16.Tetrops praeustus
COLFH1525-16.Tetrops praeustus
GBCOC360-12.Tetrops praeustus
GCOL285-16.Tetrops praeustus
GCOL3777-16.Tetrops praeustus
CERLF575-08.Tetrops praeustus

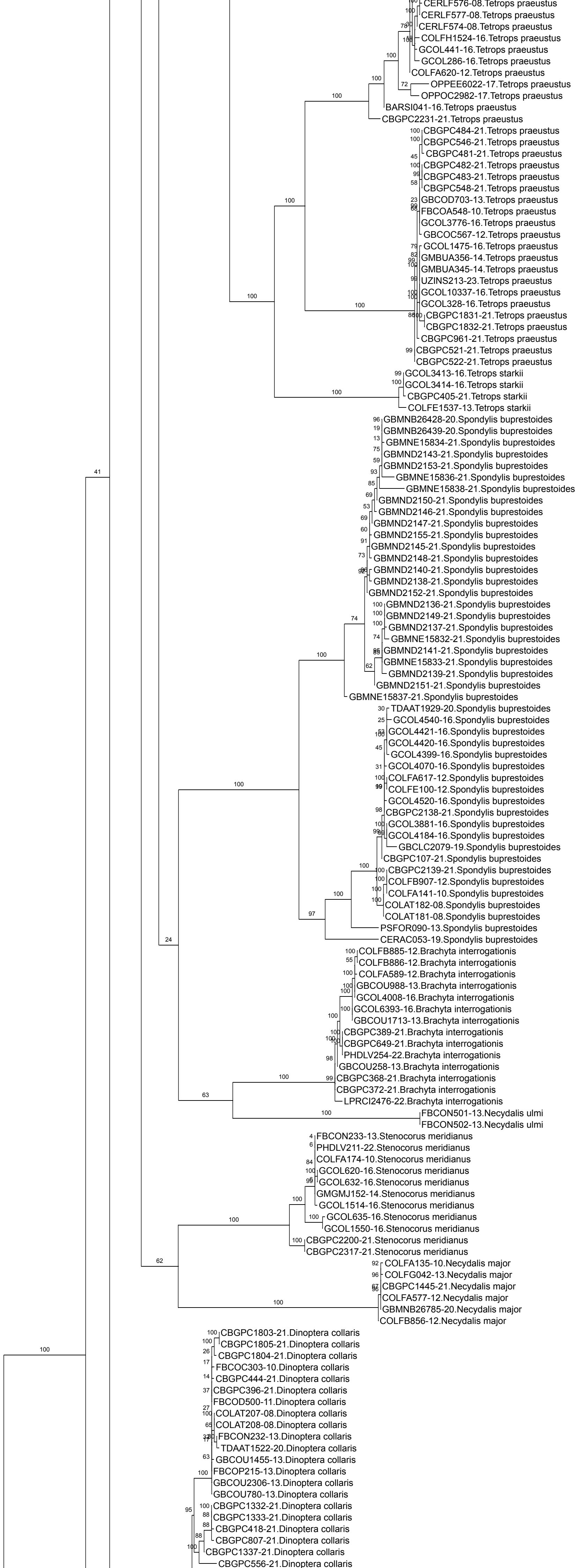
CERLF576-08.Tetrops praeustus
CERLF577-08.Tetrops praeustus
CERLF574-08.Tetrops praeustus
COLFH1524-16.Tetrops praeustus
GCOL441-16.Tetrops praeustus
GCOL286-16.Tetrops praeustus
COLFA620-12.Tetrops praeustus
OPPEE6022-17.Tetrops praeustus
OPPOC2982-17.Tetrops praeustus
BARSI041-16.Tetrops praeustus
CBGPC2231-21.Tetrops praeustus
CBGPC484-21.Tetrops praeustus
CBGPC546-21.Tetrops praeustus
CBGPC481-21.Tetrops praeustus
CBGPC482-21.Tetrops praeustus
CBGPC483-21.Tetrops praeustus
CBGPC548-21.Tetrops praeustus
GBCOD703-13.Tetrops praeustus
FBCOA548-10.Tetrops praeustus
GCOL3776-16.Tetrops praeustus
GBCOC567-12.Tetrops praeustus
GCOL1475-16.Tetrops praeustus
GMBUA356-14.Tetrops praeustus
GMBUA345-14.Tetrops praeustus
UZINS213-23.Tetrops praeustus
GCOL10337-16.Tetrops praeustus
GCOL328-16.Tetrops praeustus
CBGPC1831-21.Tetrops praeustus
CBGPC1832-21.Tetrops praeustus
CBGPC961-21.Tetrops praeustus
CBGPC521-21.Tetrops praeustus
CBGPC522-21.Tetrops praeustus
GCOL3413-16.Tetrops starkii
GCOL3414-16.Tetrops starkii
CBGPC405-21.Tetrops starkii
COLFE1537-13.Tetrops starkii
GBMNB26428-20.Spondylis buprestoides
GBMNB26439-20.Spondylis buprestoides
GBMNE15834-21.Spondylis buprestoides
GBMND2143-21.Spondylis buprestoides
GBMND2153-21.Spondylis buprestoides
GBMNE15836-21.Spondylis buprestoides
GBMNE15838-21.Spondylis buprestoides
GBMND2150-21.Spondylis buprestoides
GBMND2146-21.Spondylis buprestoides
GBMND2147-21.Spondylis buprestoides
GBMND2155-21.Spondylis buprestoides
GBMND2145-21.Spondylis buprestoides
GBMND2148-21.Spondylis buprestoides
GBMND2140-21.Spondylis buprestoides
GBMND2138-21.Spondylis buprestoides
GBMND2152-21.Spondylis buprestoides
GBMND2136-21.Spondylis buprestoides
GBMND2149-21.Spondylis buprestoides
GBMND2137-21.Spondylis buprestoides
GBMNE15832-21.Spondylis buprestoides
GBMND2141-21.Spondylis buprestoides
GBMNE15833-21.Spondylis buprestoides
GBMND2139-21.Spondylis buprestoides
GBMND2151-21.Spondylis buprestoides
GBMNE15837-21.Spondylis buprestoides
TDAAT1929-20.Spondylis buprestoides
GCOL4540-16.Spondylis buprestoides
GCOL4421-16.Spondylis buprestoides
GCOL4420-16.Spondylis buprestoides
GCOL4399-16.Spondylis buprestoides
GCOL4070-16.Spondylis buprestoides
COLFA617-12.Spondylis buprestoides
COLFE100-12.Spondylis buprestoides
GCOL4520-16.Spondylis buprestoides
CBGPC2138-21.Spondylis buprestoides
GCOL3881-16.Spondylis buprestoides
GCOL4184-16.Spondylis buprestoides
GBCLC2079-19.Spondylis buprestoides
CBGPC107-21.Spondylis buprestoides
CBGPC2139-21.Spondylis buprestoides
COLFB907-12.Spondylis buprestoides
COLFA141-10.Spondylis buprestoides
COLAT182-08.Spondylis buprestoides
COLAT181-08.Spondylis buprestoides
PSFOR090-13.Spondylis buprestoides
CERAC053-19.Spondylis buprestoides
COLFB885-12.Brachyta interrogationis
COLFB886-12.Brachyta interrogationis
COLFA589-12.Brachyta interrogationis
GBCOU988-13.Brachyta interrogationis
GCOL4008-16.Brachyta interrogationis
GCOL6393-16.Brachyta interrogationis
GBCOU1713-13.Brachyta interrogationis
CBGPC389-21.Brachyta interrogationis
CBGPC649-21.Brachyta interrogationis
PHDLV254-22.Brachyta interrogationis
GBCOU258-13.Brachyta interrogationis
CBGPC368-21.Brachyta interrogationis
CBGPC372-21.Brachyta interrogationis
LPRCI2476-22.Brachyta interrogationis
FBCON501-13.Necydalis ulmi
FBCON502-13.Necydalis ulmi
FBCON233-13.Stenocorus meridianus
PHDLV211-22.Stenocorus meridianus
COLFA174-10.Stenocorus meridianus
GCOL620-16.Stenocorus meridianus
GCOL632-16.Stenocorus meridianus
GMGMJ152-14.Stenocorus meridianus
GCOL1514-16.Stenocorus meridianus
GCOL635-16.Stenocorus meridianus
GCOL1550-16.Stenocorus meridianus
CBGPC2200-21.Stenocorus meridianus
CBGPC2317-21.Stenocorus meridianus
COLFA135-10.Necydalis major
COLFG042-13.Necydalis major
CBGPC1445-21.Necydalis major
COLFA577-12.Necydalis major
GBMNB26785-20.Necydalis major
COLFB856-12.Necydalis major
CBGPC1803-21.Dinoptera collaris
CBGPC1805-21.Dinoptera collaris
CBGPC1804-21.Dinoptera collaris
FBCOC303-10.Dinoptera collaris
CBGPC444-21.Dinoptera collaris
CBGPC396-21.Dinoptera collaris
FBCOD500-11.Dinoptera collaris
COLAT207-08.Dinoptera collaris
COLAT208-08.Dinoptera collaris
FBCON232-13.Dinoptera collaris
TDAAT1522-20.Dinoptera collaris
GBCOU1455-13.Dinoptera collaris
FBCOP215-13.Dinoptera collaris
GBCOU2306-13.Dinoptera collaris
GBCOU780-13.Dinoptera collaris
CBGPC1332-21.Dinoptera collaris
CBGPC1333-21.Dinoptera collaris
CBGPC418-21.Dinoptera collaris
CBGPC807-21.Dinoptera collaris
CBGPC1337-21.Dinoptera collaris
CBGPC556-21.Dinoptera collaris

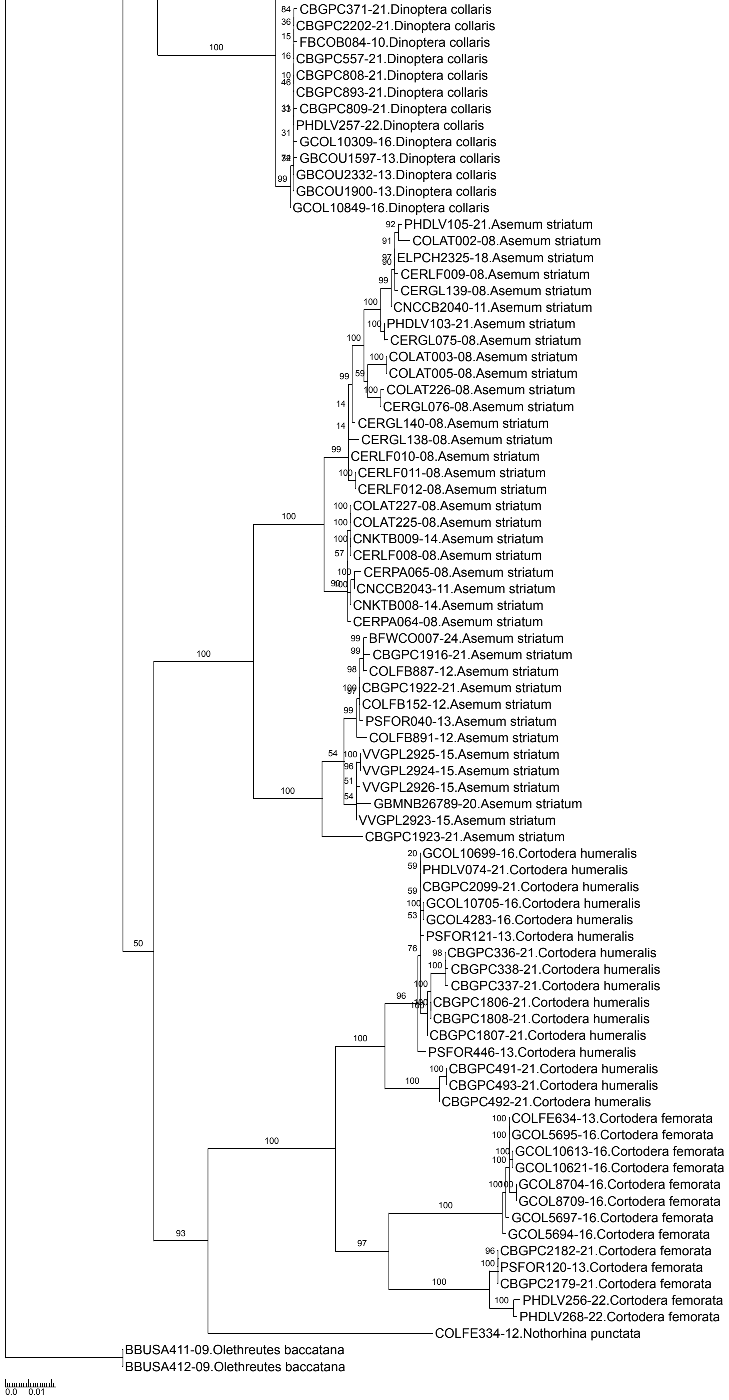
CBGPC371-21.Dinoptera collaris
CBGPC2202-21.Dinoptera collaris
FBCOB084-10.Dinoptera collaris
CBGPC557-21.Dinoptera collaris
CBGPC808-21.Dinoptera collaris
CBGPC893-21.Dinoptera collaris
CBGPC809-21.Dinoptera collaris
PHDLV257-22.Dinoptera collaris
GCOL10309-16.Dinoptera collaris
GBCOU1597-13.Dinoptera collaris
GBCOU2332-13.Dinoptera collaris
GBCOU1900-13.Dinoptera collaris
GCOL10849-16.Dinoptera collaris
PHDLV105-21.Asemum striatum
COLAT002-08.Asemum striatum
ELPCH2325-18.Asemum striatum
CERLF009-08.Asemum striatum
CERGL139-08.Asemum striatum
CNCCB2040-11.Asemum striatum
PHDLV103-21.Asemum striatum
CERGL075-08.Asemum striatum
COLAT003-08.Asemum striatum
COLAT005-08.Asemum striatum
COLAT226-08.Asemum striatum
CERGL076-08.Asemum striatum
CERGL140-08.Asemum striatum
CERGL138-08.Asemum striatum
CERLF010-08.Asemum striatum
CERLF011-08.Asemum striatum
CERLF012-08.Asemum striatum
COLAT227-08.Asemum striatum
COLAT225-08.Asemum striatum
CNKTB009-14.Asemum striatum
CERLF008-08.Asemum striatum
CERPA065-08.Asemum striatum
CNCCB2043-11.Asemum striatum
CNKTB008-14.Asemum striatum
CERPA064-08.Asemum striatum
BFWCO007-24.Asemum striatum
CBGPC1916-21.Asemum striatum
COLFB887-12.Asemum striatum
CBGPC1922-21.Asemum striatum
COLFB152-12.Asemum striatum
PSFOR040-13.Asemum striatum
COLFB891-12.Asemum striatum
VVGPL2925-15.Asemum striatum
VVGPL2924-15.Asemum striatum
VVGPL2926-15.Asemum striatum
GBMNB26789-20.Asemum striatum
VVGPL2923-15.Asemum striatum
CBGPC1923-21.Asemum striatum
GCOL10699-16.Cortodera humeralis
PHDLV074-21.Cortodera humeralis
CBGPC2099-21.Cortodera humeralis
GCOL10705-16.Cortodera humeralis
GCOL4283-16.Cortodera humeralis
PSFOR121-13.Cortodera humeralis
CBGPC336-21.Cortodera humeralis
CBGPC338-21.Cortodera humeralis
CBGPC337-21.Cortodera humeralis
CBGPC1806-21.Cortodera humeralis
CBGPC1808-21.Cortodera humeralis
CBGPC1807-21.Cortodera humeralis
PSFOR446-13.Cortodera humeralis
CBGPC491-21.Cortodera humeralis
CBGPC493-21.Cortodera humeralis
CBGPC492-21.Cortodera humeralis
COLFE634-13.Cortodera femorata
GCOL5695-16.Cortodera femorata
GCOL10613-16.Cortodera femorata
GCOL10621-16.Cortodera femorata
GCOL8704-16.Cortodera femorata
GCOL8709-16.Cortodera femorata
GCOL5697-16.Cortodera femorata
GCOL5694-16.Cortodera femorata
CBGPC2182-21.Cortodera femorata
PSFOR120-13.Cortodera femorata
CBGPC2179-21.Cortodera femorata
PHDLV256-22.Cortodera femorata
PHDLV268-22.Cortodera femorata
COLFE334-12.Nothorhina punctata
BBUSA411-09.Olethreutes baccatana
BBUSA412-09.Olethreutes baccatana
0.0
0.01
